# Mapping the effects of pregnancy on resting state brain activity, white matter microstructure, neural metabolite concentrations and grey matter architecture

**DOI:** 10.1038/s41467-022-33884-8

**Published:** 2022-11-22

**Authors:** Elseline Hoekzema, Henk van Steenbergen, Milou Straathof, Arlette Beekmans, Inga Marie Freund, Petra J. W. Pouwels, Eveline A. Crone

**Affiliations:** 1 Hoekzema Lab, Amsterdam University Medical Center (UMC), location University of Amsterdam, Department of Psychiatry, Amsterdam Neuroscience, Amsterdam Reproduction and Development, Amsterdam, the Netherlands; 2 Brain and Development Research Center, Leiden Institute for Brain and Cognition, Leiden University, Leiden, the Netherlands; 3 Cognitive Psychology Unit and Leiden Institute for Brain and Cognition, Leiden University, Leiden, the Netherlands; 4 Amsterdam University Medical Center (UMC), location Vrije Universiteit Amsterdam, Department of Radiology and Nuclear Medicine, Amsterdam Neuroscience, Amsterdam, the Netherlands

**Keywords:** Social neuroscience, Neuroscience

## Abstract

While animal studies have demonstrated a unique reproduction-related neuroplasticity, little is known on the effects of pregnancy on the human brain. Here we investigated whether pregnancy is associated with changes to resting state brain activity, white matter microstructure, neural metabolite concentrations and grey matter architecture using a comprehensive pre-conception cohort study. We show that pregnancy leads to selective and robust changes in neural architecture and neural network organization, which are most pronounced in the Default Mode Network. These neural changes correlated with pregnancy hormones, primarily third-trimester estradiol, while no associations were found with other factors such as osmotic effects, stress and sleep. Furthermore, the changes related to measures of maternal-fetal bonding, nesting behavior and the physiological responsiveness to infant cues, and predicted measures of mother-infant bonding and bonding impairments. These findings suggest there are selective pregnancy-related modifications in brain structure and function that may facilitate peripartum maternal processes of key relevance to the mother-infant dyad.

## Abstract (web summary)

Animal studies have shown that pregnancy is associated with unique changes in the mammalian brain and behaviour, although pregnancy-associated changes in the human brain are less well studied. Here the authors show that pregnancy is associated with changes in resting state brain activity and brain anatomy which are most pronounced in the default mode network.

## Introduction

Pregnancy is a highly extreme biological process that marks a monumental transition in a woman’s life. This period involves an intricate cascade of unparalleled endocrine changes, which orchestrate numerous adaptations in a woman’s body. Practically all bodily systems are affected during pregnancy, involving long-lasting changes in a woman’s physiology that remain for decades after giving birth^1^. However, in line with the general shortage of research on women’s health and biology^2–4^, the impact of this unique process on the human brain has long remained a virtually unexplored territory.

A converging body of evidence has demonstrated that reproduction is associated with a unique and dramatic brain plasticity in various species of non-human animals, involving pronounced changes in the mammalian brain and behaviour^5–9^. We have previously shown that pregnancy renders changes in the grey matter structure of the human brain^10^. In the current study, we set out to investigate how becoming a mother changes a woman’s white matter microstructure, neural metabolite concentrations and neural network organization, using a comprehensive prospective cohort study in which women were followed from pre-conception until the late postpartum period and took part in four longitudinal experimental sessions. This project involved anatomical magnetic resonance imaging (MRI), diffusion-weighted imaging, 1H-nuclear magnetic resonance spectroscopy and resting state functional MRI acquisitions, allowing us to comprehensively investigate the impact of pregnancy on the human brain. Furthermore, to investigate the mechanisms driving pregnancy-related neuroplasticity, we acquired a profile of hormonal changes based on biological samples collected every four weeks of pregnancy in combination with experiential factors such as sleep and stress. Finally, we aimed to pinpoint potential functional implications of pregnancy-related neuroplasticity by examining the relation to key gestational and postpartum maternal processes.

Using these data, we (i) examined whether pregnancy renders changes in grey matter architecture, diffusion metrics, neural metabolite concentrations and the temporal coherence within and between neural networks by comparing the pre-pregnancy and post-pregnancy brain scans of the women who became pregnant during this study in comparison to the women who remained nulliparous, (ii) investigated the long-term persistence of these brain changes with a 1-year late postpartum follow-up session, (iii) tested the contribution of various potential biological and experiential factors, (iv) examined the relation to gestational maternal physiological and psychological measures, and (v) tested whether these neural changes related to measures of mother-infant bonding and impairments in the mother-infant relationship.

In this study, we comprehensively chart the changes manifesting in a woman’s brain across this unique transitional period. Our data reveal a pronounced and selective structural and functional brain plasticity, which may confer adaptive advantages for a mother’s gestational and maternal behaviour and the establishment of the new mother-child relationship.

## Results

### Grey matter structure

This prospective cohort study involved four sessions (pre-conception (‘Pre’), late pregnancy (‘Prg’), post-pregnancy (‘Post’), late postpartum (‘Post+1 y’)). Except for the session during pregnancy, each of these sessions involved MRI acquisitions, which included high-resolution anatomical brain scans. To examine the effects of becoming a mother on the brain’s grey matter (GM) structure, each individual’s post-pregnancy brain scan was analyzed in relation to their pre-pregnancy scan, allowing us to reliably extract the changes in brain structure relative to each person’s pre-conception baseline. Longitudinal data were also acquired at a comparable time interval from nulliparous control women. Complete Pre-Post longitudinal datasets were acquired from 40 nulliparous women who were pregnant between sessions (the PRG group) and 40 nulliparous women who did not become pregnant during this study (the CTR group).

**Fig. 1:**
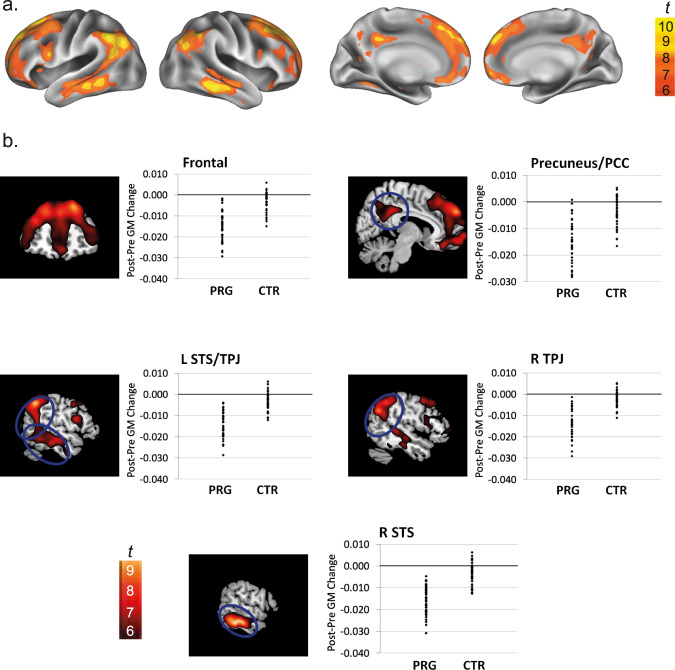
Grey matter volume changes across pregnancy (PRE to POST). Grey matter volume changes in the women who were pregnant between sessions (PRG) in comparison to the women who were not (CTR). **a** Surface maps of the grey matter volume changes, displayed at a whole-brain peak voxel FWE-corrected threshold of *p* < 0.05. **b** Coronal and sagittal slice overlays and plots displaying individual grey matter volume changes for each woman in the PRG (*N* = 40) and the CTR (*N* = 40) group extracted from the smoothed normalized Jacobian difference images for each cluster. Due to the large number of clusters resulting from these analyses, only the most significant clusters (T > 8) are displayed. Source data are provided as a Source Data file. GM grey matter, L left, R right, PCC posterior cingulate cortex, STS superior temporal sulcus, TPJ temporo-parietal junction.

The longitudinal diffeomorphic modeling pipeline implemented in SPM12 was applied to extract changes in GM volume between the subsequent brain scans on an individual level, and the maps of GM volume change of the primiparous women were compared to those of the nulliparous control women. Baseline comparisons indicated that there were no pre-existing differences in GM volume between the groups. When investigating the GM volume changes across sessions, we observed a symmetrical pattern of highly significant group differences (Fig. 1, Supplementary Table 1), and post hoc analyses revealed that each of these clusters reflected reductions in regional GM in the women who were pregnant between the time points (Supplementary Table 2). Computations of the effect size indicate that these correspond to very large effects (Supplementary Fig. 1).

The GM volume reductions were primarily centred on the bilateral superior temporal sulcus and temporo-parietal junction as well as on the anterior and posterior midline of the brain, including clusters located in the precuneus and posterior cingulate cortex and the medial prefrontal cortex extending to various lateral frontal areas. These findings are remarkably similar to the results obtained in our previous independent sample^10^, replicating GM volume reductions in all of the previously observed clusters, although the current study demonstrates even more significant and larger clusters that extend to other regions of the brain such as the bilateral temporo-parietal junction.

For completeness, to further examine the robustness of these findings and account for potential confounds, we also repeated the main model while taking into account various confounding factors. Additional models were performed in which for instance the women who became pregnant by means of fertility treatment or who delivered twins were excluded, the participants’ medical history was accounted for, or the women with a long Pre-Post time interval were excluded from the analysis (see Methods section, Supplementary Tables 3–6). All these models rendered nearly identical results, indicating that these potential confounds did not play a significant role in the observed findings. Finally, we also repeated our analysis corrected for total brain volume (Supplementary Tables 7 and 8), which indicated that the observed changes in regional grey matter volume are also evident when correcting for global changes in brain volume (Supplementary Table 9).

To objectively measure the localization of the observed changes in brain structure, we quantified the spatial correspondence of our changes with the brain’s cognitive components and the resting state neural networks defined by Yeo et al.^11,12^. Spatial overlap quantifications with the cognitive components of the human association cortex indicated the strongest overlap with component 10—the component most recruited by theory of mind tasks and during rest—and component 9, which is recruited by various higher-level cognitive tasks targeting for instance cognitive flexibility (Supplementary Table 10).

Furthermore, we performed computations of the spatial correspondence with the networks of intrinsic functional connectivity defined by Yeo et al.^12^. A visual inspection of our structural changes in relation to a map of their intrinsic neural network ontology suggests a strong similarity between the DMN and the pattern of structural brain changes observed in our study (Supplementary Fig. 2). This was confirmed by the analyses of overlap quantification, revealing the strongest overlap with the Default Mode Network (DMN) (Supplementary Table 11). For completeness, we also computed the spatial similarity with the networks of Smith et al.^13^, which rendered the strongest overlap with the fronto-parietal network and the DMN (Supplementary Table 12).

### Neural network organization

**Fig. 2:**
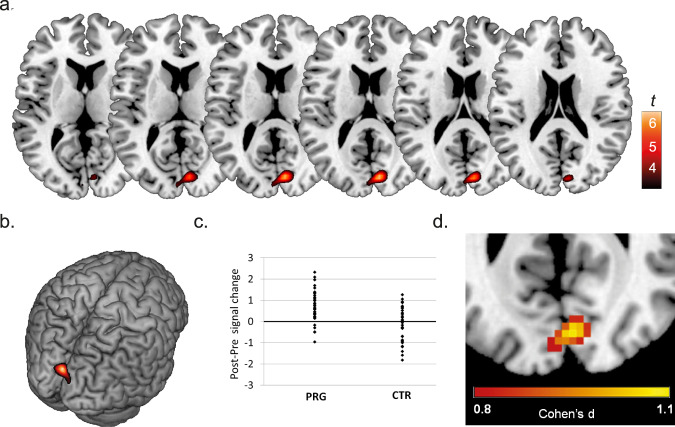
Changes in DMN coherence across pregnancy (PRE to POST). **a** Increases in DMN within-network connectivity in the women who became pregnant during the study, overlaid on axial slices. **b** Surface maps depicting changes in DMN coherence. For illustrative purposes, changes are shown at *p* < 0.0001 uncorrected. **c** Scatter plots depicting individual changes in DMN coherence per group. Source data are provided as a Source Data file. **d** Illustration of effect sizes (Cohen’s d) for the changes in within-network coherence of the Default Mode Network between sessions in the women who became pregnant (*N* = 40) in comparison to the women who did not (*N* = 36). Depicted effect sizes correspond to large effect sizes (Cohen’s d > 0.8). PRG = women who became pregnant during this study, CTR = women who remained nulliparous during this study. Pre = pre-pregnancy session, Post = post-pregnancy session.

Considering the strong structural changes observed in the DMN across pregnancy, we were particularly interested whether pregnancy also renders changes in the intrinsic functional activity within this network. Resting state fMRI data were acquired during each of the MRI sessions and analyzed using Independent Components Analyses, rendering networks of intrinsic functional connectivity. When investigating the changes in temporal coherence within the neural networks in the women who were pregnant between sessions in comparison to the women who remained nulliparous, we observed a significant cluster for the DMN comparisons located in the bilateral cuneus (Fig. 2, Supplementary Table 13). This represents a large effect, as illustrated by the effect size figure in (Fig. 2d). No significant differences were observed between the PRG and CTR groups in changes in within-network connectivity for any of the other neural networks.

Further analyses involving the DMN maps demonstrated that the observed effect reflected an increase in functional connectivity in the women who were pregnant between sessions (Fig. 2, Supplementary Table 14), indicating that becoming a mother renders increases in the DMN’s temporal coherence. For completeness, paired samples *t* tests were also performed for all other resting state networks. Apart from the observed increase in DMN coherence, these analyses only rendered an increased coherence in one of the visual networks (Supplementary Table 14). However, as indicated, this effect did not surface in an interaction contrast examining the Pre-to-Post differences between the PRG and CTR groups.

Analyses testing for baseline differences in DMN connectivity revealed no group differences within the relevant cluster, although baseline differences were observed in two other areas (Supplementary Table 15). While these baseline differences are not located in the relevant cluster, it is a possibility that these pre-existing differences might be associated with the subsequent changes in DMN coherence. Therefore, we also performed correlation analyses between the baseline signal within these regions and the observed changes in DMN coherence across pregnancy, which did not point to an association (Supplementary Table 16).

Additional models testing for the impact of potential confounds such as fertility treatment, delivery of twins and medical history rendered highly similar results (Supplementary Tables 17–21). Complementary to the analyses of within-network coherence, we additionally investigated the connectivity between the different resting state networks. No significant changes were observed in between-network coherence in the women who became pregnant in comparison to the nulliparous control women (Supplementary Tables 22 and 23). These findings thus indicate that becoming a mother is associated with selective changes in the temporal coherence of the default mode network of the brain.

### White matter microstructure

The microstructure of white matter tracts was investigated by means of Diffusion-Weighted Imaging and Tract-Based Spatial Statistics, allowing us to investigate whether becoming a mother is associated with focal changes in the diffusion metrics. Based on the acquired Diffusion-Weighted MRI scans, individual maps of change in fractional anisotropy (FA), mean diffusivity (MD), axial diffusivity (AD) and radial diffusivity (RD) between the pre-pregnancy and post-pregnancy sessions were extracted. Comparisons between the women who were pregnant between the sessions and the women who remained nulliparous revealed no significant differences in any of these measures, suggesting that becoming a mother does not render pronounced changes in white matter microstructure.

### Neural metabolites

To examine whether pregnancy results in changes in neural metabolite concentrations, we analyzed metabolite concentrations of tNAA (N-acetylaspartate including contributions from N-acetylaspartylglutamate), tCr (creatine and phosphocreatine), Cho (phosphorylcholine and glycerophosphorylcholine), Glu (Glutamate), and Ins (myo-Inositol) within one of the clusters of strongest volume reductions across pregnancy^10^, the precuneus/posterior cingulate cortex. Baseline comparisons revealed no differences between the groups on any of these measures (Supplementary Table 24). When examining the changes in neural metabolites between the pre-pregnancy and post-pregnancy sessions, we observed no differences between the groups for tNAA and Glu (Supplementary Table 25), but we observed increases for Cho, Ins and tCr (Supplementary Table 25). However, it should be noted that these represent relatively weak effects (Supplementary Table 26) and they do not survive a correction for multiple comparisons. Accordingly, these effects are no longer evident when correcting for various potential confounds such as exposure to fertility treatment, delivery of twins or a relatively long time interval between sessions (Supplementary Tables 27–30).

As a supplementary analysis, correlation analyses were performed to test for associations between the modalities in which any effects were observed (i.e. the observed changes in grey matter, resting state fMRI data and neural metabolites), which showed an association between changes in grey matter and myo-inositol (Supplementary Table 31).

### Persistence of effects across postpartum period

To investigate the persistence of the observed effects in brain structure and function across the postpartum period, we examined the neuroimaging data acquired from a late postpartum follow-up session around 1 year after giving birth (the Post+1 y session). Full longitudinal datasets of women investigated from pre-conception until the late postpartum period were available for twenty-eight women.

**Fig. 3:**
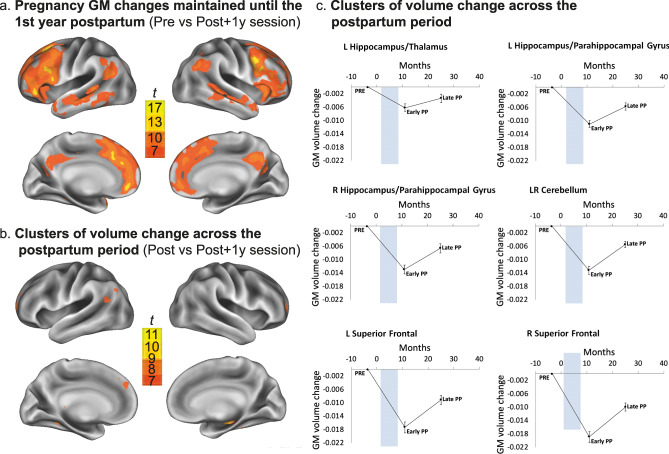
Late postpartum follow-up session (Post + 1 y). Grey Matter Volume Changes in in first-time mothers from pre-conception until the late postpartum period. Complete Pre, Post, and Post+1 y datasets were available of 28 primiparous women. **a** Surface maps of grey matter volume reductions in the late postpartum (Post + 1 y) session compared to the Pre-pregnancy baseline (Pre), displayed at a peak voxel whole-brain FWE-corrected threshold of *p* < 0.05. **b** Surface maps of the grey matter volume increases at the late postpartum session (Post + 1 y) compared to the early postpartum session (Post), displayed at a peak voxel whole-brain FWE-corrected threshold of *p* < 0.05. **c** Mean (±SEM) grey matter volume changes at each Post session (Post and Post+1 y) relative to the Pre-pregnancy baseline of the most significant clusters (i.e., T > 8), extracted from the smoothed normalized Jacobian difference images for each cluster. Source data are provided as a Source Data file. The control group is plotted in Supplementary Fig. 3. Blue bar represents pregnancy. GM grey matter, L left, R right, PP postpartum.

When examining the changes in the women’s grey matter structure between this Post+1 y session and the pre-pregnancy baseline, we observed a pattern of GM volume reductions that was highly similar to the pattern observed when comparing the early postpartum session to the pre-pregnancy baseline, indicating that pregnancy-related brain changes are still evident at 1 year postpartum (Fig. 3a, Supplementary Table 32). However, when comparing the early postpartum session to the late postpartum session, we observed several clusters of GM volume change, indicating volume increases across the postpartum period where volume partially reversed to pre-pregnancy levels (Fig. 3b, c, Supplementary Fig. 3, Supplementary Table 33). In agreement with our previous results^10^, the most significant volume increases were observed in the hippocampal complex (Fig. 3c, Supplementary Table 33), although in the current study postpartum volume increases were also found in various other brain regions.

To examine the persistence of the observed changes in neural network organization, analyses involving the resting state functional MRI data acquired during the Post+1 y follow-up session were performed. When comparing the within-network connectivity maps of the resting state networks between the early and late postpartum sessions, no significant changes were observed. However, when applying a region of interest of the cluster of increased DMN coherence to the analyses of postpartum DMN changes, a significant effect surfaced within this region (MNI coordinates(x y z)= 0 −84 12, T = 3.20, *p* = 0.028 FWE-corrected), indicating a partial reversal of this effect across the postpartum period (Supplementary Fig. 4).

### Functional implications

Periods of strong coordinated hormone influxes generally represent windows of pronounced functional change^14–16^. Based on animal studies showing that pregnancy-related neuroplasticity facilitates the induction of maternal preparatory behaviours and caregiving responses^5–9,17^, we were primarily interested in investigating whether pregnancy-related neural changes are associated with the stimulation of gestational maternal processes that facilitate the preparation for parturition and motherhood. Therefore, we investigated whether the changes in grey matter volume and DMN coherence related to the pregnant women’s (i) maternal-foetal attachment, (ii) physiological reactions to infant cues, and (iii) nesting behaviour. Finally, we additionally examined whether these neural changes were associated with postpartum measures of (i) mother-infant bonding and (ii) impairments in the mother-infant relationship.

One of the first expressions of maternal behaviour in non-human animals is nesting behaviour, which refers to a wide range of behaviours to prepare for parturition such as nest-site selection, nest building and nest defence. Women also display forms of nesting behaviour and urges, which peak in the third trimester of pregnancy and primarily concern processes of space preparation and social selectivity^18^. Our analyses involving measures of nesting behaviour showed no associations with changes in DMN coherence (Supplementary Table 34, Supplementary Fig. 5). However, the changes in grey matter structure across pregnancy were associated with both domains of nesting behaviour (the familiarity preference subscale of the social selectivity domain and the energy burst subscale of the space preparation domain), but only the first correlation survives a correction for multiple comparisons for the number of tests within the research question (Supplementary Figs. 5 and 6, Supplementary Table 35), thus suggesting that the observed grey matter changes across pregnancy are related to aspects of the pregnancy-specific behavioural urges and activities that facilitate preparing for parturition and the arrival of a woman’s baby. This effect also survives a correlation-adjusted Bonferroni correction across all applied prenatal measures.

During pregnancy, many mothers start to develop an attachment to their foetus. Here we investigated whether the changes in a woman’s brain during the transition to motherhood are related to measures of maternal-foetal bonding measured with the Prenatal Attachment Inventory and the Maternal Antenatal Attachment Scale. These analyses demonstrated that the changes in neural coherence of the default mode network are associated with the degree to which a pregnant woman differentiates her foetus from herself and thinks of him/her as an individual, with a stronger increase in DMN coherence being associated with a stronger differentiation (Supplementary Figs. 5 and 6, Supplementary Tables 36). However, it should be noted that, while this effect remained significant when correcting for multiple comparisons within the research question, it did not survive a Bonferroni correction across all prenatal measures. No correlations were observed with grey matter structure (Supplementary Table 37, Supplementary Fig. 5).

In addition to the women’s psychological reactions to their foetus, we investigated their physiological reactions to infant cues. We monitored the women’s interval between heart rate peaks and galvanic skin response to movies of crying and laughing babies. Correlation analyses involving these measures indicated that the women’s heart rate response to laughing infants was negatively correlated with DMN coherence (corrected for multiple comparisons across all prenatal measures), indicating a heart rate deceleration when exposed to these positive infant stimuli (Supplementary Figs. 5 and 6, Supplementary Tables 38 and 39). Together, these findings thus suggest that pregnancy-related brain changes are associated with a pregnant woman’s nesting behaviour, physiological reaction to infant cues and maternal-foetal attachment.

Finally, we also examined whether the observed brain changes across pregnancy were associated with a mother’s bonding to her infant in the postpartum period using the Maternal Postnatal Attachment Scale (MPAS). In addition, we tested for associations with problems in the postpartum mother-infant relationship using the Postpartum Bonding Questionnaire (PBQ). These analyses, which are described in more detail in the Supplementary Material (Supplementary Note 1, page 29 of the Supplementary Material), revealed no associations with mother-infant bonding or bonding impairments in the early postpartum period. However, changes in default mode activity across pregnancy significantly related to both mother-infant bonding and impairments in the mother-infant relationship in the late postpartum period (Supplementary Note 1, Supplementary Fig. 7, Supplementary Tables 40–45), with stronger DMN changes being associated with more mother-infant bonding and less bonding impairments.

Furthermore, supplementary analyses involving postpartum changes in bonding showed that pregnancy-related DMN changes related to subsequent developments in mother-infant bonding and bonding impairments across the postpartum period. More specifically, stronger brain changes were associated with relatively stronger increases in mother-infant bonding and the pleasure experienced by the mother in the interaction with her infant and less infant-directed hostility (Supplementary Figs. 8 and 9, Supplementary Tables 46–48). Accordingly, stronger DMN coherence changes across pregnancy were associated with a decreased risk for the postpartum development of impairments in the mother-infant relationship, infant rejection and pathological anger.

When applying a correlation-adjusted Bonferroni correction across all postnatal measures, the correlations between the changes in DMN coherence and the postpartum changes in mother-infant bonding and infant rejection and pathological anger remained significant. These findings thus reveal associations between pregnancy-related functional neuroplasticity and the development of mother-infant bonding and bonding impairments across the postpartum period, suggesting that these neurally-regulated pregnancy effects on maternal caregiving may play a role in the subsequent bonding of a mother to her infant across the postpartum period and can potentially have a long-term impact for the mother-infant dyad.

### Contributing factors

To investigate the mechanisms driving these pregnancy-related brain changes, we examined their relation with key biological and experiential factors. We hypothesized that pregnancy hormones represent the primary factors triggering and regulating pregnancy-related neuroplasticity. Therefore, urine samples were collected at 10 time points during pregnancy and were used to determine levels of estradiol, estriol, cortisol and progesterone and acquire an indication of the hormone levels the women were exposed to throughout their pregnancy. Correlation analyses involving these hormones revealed associations between the observed changes in grey matter volume and the estrogens estradiol and estriol, although only the correlation with estradiol survived a correction for multiple comparisons (Supplementary Tables 49 and 50).

The weight map associated with the regression model for estradiol—reflecting the relative contribution of the voxels across the brain—indicates that the regions of strongest neural changes also strongly contribute to this regression (Supplementary Fig. 10). In fact, the weight map shows a remarkable similarity to the map of observed brain changes across pregnancy, suggesting that the levels of estradiol a woman is exposed to during gestation may exert effects across the brain and are likely to contribute to the overall pattern of observed neural changes.

Supplementary analyses involving the different sampling points of estradiol measurements across pregnancy were then performed to examine the timing of these effects. These analyses indicated associations with estradiol levels sampled in week 12 as well as a trend for an association with week 24 estradiol levels. Furthermore, associations or trends were observed for estradiol levels extracted from every sampled week of the third trimester (Supplementary Table 51), indicating that especially third-trimester estradiol levels are associated with the observed brain changes.

In addition to these endocrine measures, various other factors were investigated to examine whether we could find indications of a potential contribution to the observed brain changes. More specifically, we tested for a potential impact of the following factors: (i) osmolality, (ii) sleep, (iii) stress, (iv) the duration of postpartum factors, (v) breastfeeding, (vi) the type of delivery.

To examine the impact of osmotic effects, we performed correlation analyses between the changes in brain structure and function and levels of urine osmolality in pregnancy. These analyses rendered no significant results (Supplementary Tables 52 and 53), suggesting that pregnancy-related osmotic changes do not play an important role in driving these neural modifications. In addition, since previous studies have found associations between gestational osmolality and several metabolites^19^, we also checked for a correlation between neural metabolite concentrations and osmolality levels in pregnancy. Indeed, we found indications for associations between Glu, Ins, tsar and Cho and third-trimester urine osmolality (Supplementary Tables 54–58), suggesting that changes in these metabolites across pregnancy may reflect an effect of pregnancy-induced osmotic processes.

To test for an association with sleep and stress, various measures of stress were acquired during pregnancy and the postpartum period as well as various measures of sleep involving the hours of sleep and the number of sleep disruptions during pregnancy and the postpartum period. In addition, potential relations were investigated with the number of days between delivery and the Post session, reflecting the duration of exposure to postpartum factors. Furthermore, we examined the potential contribution of breastfeeding and the type of delivery to the observed changes in brain structure and function. Analyses involving these measures rendered no significant results. For completeness, we also included each of these measures as confounding factors in the main analyses, which did not significantly alter the results. All of these results are discussed in more detail in Supplementary Note 2 (page 40 and 41 of the Supplementary Material) and reported in Supplementary Tables 59–92. Collectively, these data suggest that stress, sleep, the duration of exposure to postpartum factors, breastfeeding and the type of delivery do not represent major factors contributing to the observed brain changes.

Interestingly, a supplementary analysis examining the association between the total months of breastfeeding until the Post+1 y session with changes in grey matter volume and DMN coherence across the postpartum period revealed a positive correlation between the degree of reversal of DMN coherence across the postpartum period and the duration of breastfeeding (Supplementary Tables 93 and 94), suggesting that prolonged breastfeeding may play a role in stimulating the maintenance of these pregnancy-related neural changes across the postpartum period.

## Discussion

Pregnancy represents a monumental transition and one of the most extreme endocrine events of life. This study aimed to chart the impact of this unique neurobiological journey on a woman’s brain structure, metabolism and neural network organization and to acquire insights into the mechanisms driving these neural changes and their functional implications. When investigating the women’s GM structure, we observed a highly significant pattern of GM volume reductions in women who were pregnant between sessions in comparison to women who were not, which primarily affected the anterior and posterior cortical midline and specific sections of the bilateral lateral prefrontal and temporal cortex. These findings are in agreement with our previous observations of structural brain changes in an independent sample^10^, thus demonstrating the reliability of these findings and their generalizability across women from different countries.

Furthermore, the current study involved diffusion-weighted imaging, magnetic resonance spectroscopy and resting state functional MRI, allowing us to examine for the first time whether becoming a mother is associated with changes in neural network organization, neural metabolites and white matter structure. In contrast to the highly pronounced changes in grey matter structure, no significant changes were observed in white matter diffusion metrics or volume in the women in the pregnancy group in comparison to the nulliparous control women, suggesting that a woman’s white matter anatomy remains relatively stable throughout this period. Sex steroid hormones are known to act as important regulators of neuronal morphology and number^20^, and the current observations might reflect a particularly strong sensitivity of components in the brain’s grey matter relative to those residing in white matter structure to fluctuations in pregnancy hormones. Accordingly, in mammalian adulthood, sex hormones are thought to induce neuroplasticity in the central nervous system primarily by modulating dendritic spine and synapse density^21,22^. Although we cannot discern the cellular processes underlying the observed macroscopic changes using these techniques, our findings indicate that pregnancy-related changes represent a selective process that particularly strongly affects certain components of the brain.

When analyzing the magnetic resonance spectroscopy data, no robust changes were observed in neural metabolite concentrations across pregnancy. While we found indications for changes in myo-inositol, choline and creatine concentrations, these effects did not survive a correction for multiple comparisons nor did they surface in analyses correcting for various confounding factors. Further analyses involving these metabolites revealed associations with third-trimester osmolality levels, suggesting that these neural metabolite concentrations may relate to transient osmotic changes associated with pregnancy.

The women’s resting state functional MRI data, which were used to investigate neural network organization by means of analyses investigating the temporal coherence within and between neural networks, revealed a selective increase in coherence in the DMN in women who were pregnant between sessions in comparison to the control group. This concerned an increased DMN coherence in the cuneus. The cuneus plays a key role in visual processing and the integration of visual information with higher-order processes such as working memory, attention and reward expectation but is also known to be activated during rest and self-referential processing^23^.

In agreement with the observed functional changes in the DMN, quantification analyses of the spatial correspondence with resting state neural networks indicated that the structural brain changes are also most pronounced in the DMN, although fronto-parietal brain regions involved in higher-order cognitive tasks such as cognitive flexibility also seem to be relatively strongly affected. Taken together, our anatomical and resting state MRI data thus indicate that pregnancy is associated with a structural and functional plasticity within the DMN, suggesting that becoming a mother alters the baseline state of the brain.

The DMN represents a group of highly interconnected and coherently activated brain regions that is most active in the absence of a specific task, providing a reflection of the brain’s baseline activity^24,25^. The DMN is involved in self-referential processing, such as self-related mental explorations, autobiographical memory and self-perception^23,24^. Furthermore, the DMN is also strongly engaged by other-referential processing, including higher-order social processes such as social cognition, social evaluation and empathy^24–26^, postulated to reflect the predisposition of humans for social processing as the default mode of thought^26^.

New mothers may be required to focus on their infants and be in tune with his/her thoughts, needs and feelings, and becoming a mother has been associated with an identity transformation from being focused on the self to being focused on the infant. Accordingly, previous studies in mothers point to functional alterations in the DMN^27,28^, and indications have been found that resting state functional connectivity in mothers is associated with maternal behaviour^29,30^ and the number of children parented^31^. The DMN plays a key role in the differentiation between self and others, and the cuneus is known to be involved in the brain’s representation of the self^23^. Interestingly, when investigating the potential relation between the neural changes across pregnancy and maternal-foetal bonding, we found that the changes in coherence in the DMN were associated with the degree to which a pregnant woman differentiates her foetus from herself. Based on the observed findings, we can speculate that the observed structural and functional pregnancy-associated modifications in the DMN alter the neural basis of the self, hereby potentially contributing to the transformation in a woman’s identity and focus that often accompany new motherhood.

Periods of strong coordinated hormone influxes and neural changes generally mark windows of rapid and pronounced functional change^14–16^. In non-human animals, the most obvious behavioural change in the peripartum period concerns the expression of preparatory and caregiving behaviours. In this study, as discussed, associations were found between pregnancy-related functional changes in the DMN and facets of the antenatal bonding of a mother to her foetus, concerning the degree of differentiation of her foetus from herself. Furthermore, we tested whether the neural changes of pregnancy related to nesting behaviour and the women’s physiological responses to infant cues. Nesting behaviour represents one of the first expressions of maternal behaviour in non-human animals, and some forms of nesting behaviour have also been identified in humans^18^. We observed associations between the structural brain changes manifesting in a woman’s brain across pregnancy and aspects of nesting behaviour, suggesting that pregnancy-related neural restructuring may play a role in the stimulation of pregnancy-specific behavioural urges and activities that help a woman to prepare for the arrival of her baby.

Furthermore, animal studies have shown that pregnancy is associated with a change in the reaction to the young. For instance, pregnancy or pregnancy-mimicking hormone treatments have been found to render changes in the hedonic value allocated to young animals, with cues of the young becoming highly rewarding stimuli and potent reinforcers^5–9^. In our sample, the increases in DMN coherence were associated with a slowing of the women’s heart rate in response to laughing babies, which are highly rewarding for a mother and strongly activate the brain’s reward circuit^32,33^. Heart rate deceleration, which is a classic measure of orienting to novel or salient stimuli^34^, can reflect an increased attention to a stimulus, pointing to the possibility that the neural changes observed in women’s brains across pregnancy might facilitate a woman’s physiological reaction to this now highly rewarding and salient stimulus. Together, these findings suggest that pregnancy-related brain changes may stimulate maternal processes that help a pregnant woman prepare for the pending transition, wherein functional changes in the DMN could be particularly associated with infant-directed processes while structural brain changes may be more related with the stimulation of preparatory behaviours.

After giving birth, mammalian mothers display an extensive repertoire of maternal behaviours to take care of the young^5–9^. Hormonal priming of the brain during pregnancy and parturition plays a central role in the subsequent induction of maternal behaviours and the suppression of aversive reactions to the young. In our study, the observed neural changes in the DMN were found to relate to the degree of mother-infant bonding and impairments in the mother-infant relationship in the late postpartum period, pointing to a long-term impact for the mother and child. While these findings are in accordance with our previous results pointing to a relation between pregnancy-related brain changes and postpartum mother-infant bonding, it should be noted that the previously observed association concerned correlations between the MPAS and brain structure while in the current study all observed postpartum associations involve the functional changes in DMN coherence.

Interestingly, associations were also observed between the neural changes across pregnancy and the subsequent development of mother-infant bonding across the postpartum period, including correlations with further changes in measures of infant-directed hostility and the pleasure experienced by the mother in the interaction with her infant. Accordingly, pregnancy-related DMN changes related to postpartum changes in mother-infant bonding impairments and the risk of infant rejection and pathological anger. These findings suggest that the neural changes of pregnancy may render a blueprint that facilitates the subsequent development of the mother-infant relationship, which could then potentially be further reinforced by the interaction with the infant. Collectively, our results show that the changes occurring in a woman’s brain during this remarkable neurobiological transition are associated with various core peripartum maternal processes that benefit the mother-infant dyad. Similar to other mammals, reproduction-related brain changes in humans may thus be involved in the stimulation of maternal behaviours such as peripartum preparatory and caregiving behaviours and the suppression of negative reactions to the infant.

A session in the late postpartum period was included in the study to examine whether the observed neural changes are persistent across the postpartum period. Neuroimaging studies investigating older women have shown anatomical and functional differences between mothers and non-mothers in late life^35–37^, suggesting that motherhood is associated with long-lasting changes. Analyses involving the late postpartum follow-up session showed both changes that were maintained across the postpartum period as well as changes that partially reversed across this period. In agreement with our previous study^10^, the most significant increases in grey matter volume were observed in the hippocampal complex. However, in the current study these postpartum volume increases also involved other areas of the brain, such as the cerebellum and superior frontal cortex, possibly reflecting a higher sensitivity based on the larger subject sample. In accordance with the observed persistent and transient changes, animal studies have shown both long-lasting and short-term reproduction-related neural and behavioural changes^1^. It can be speculated that some neural modulations might be most essential in the early postpartum period, when the highly altricial child is most dependent on the mother and has the most limited communication abilities, while the postpartum brain both partially reverts to its previous state and continues to adapt to the child’s development. Longitudinal fMRI studies have shown alterations in brain activity across the postpartum period and associations with parental experience, possibly reflecting dynamic adaptations to the child’s continuous development^38,39^. It is interesting to note that in our study the total duration of breastfeeding positively correlated with the degree to which the increased DMN coherence reverted across the postpartum period, suggesting that prolonged breastfeeding may stimulate a prolonged maintenance of pregnancy-related neural changes.

To investigate the factors driving pregnancy-related brain plasticity, we tested the contribution of several key biological and experiential factors. These analyses point to a major contribution of pregnancy hormones to the changes in brain structure, particularly third-trimester estradiol levels, although no associations were observed with the changes in DMN coherence. No correlations were observed with any of the other investigated factors such as osmotic effects, sleep, stress, breastfeeding or the type of delivery. Pregnancy involves surges of sex steroid hormones that are unequalled by any period of life and much more subtle and gradual changes in sex steroid hormone levels than those associated with pregnancy are known to render changes in human brain structure and function^40–42^. The endocrine climate of pregnancy thus represents a likely candidate for anatomically shaping the female brain during this transitional period. The observed findings suggest that especially the unparalleled estrogen exposure in the final stages of pregnancy may play a primary role in the induction of pregnancy-related structural neuroplasticity.

However, endocrine influences likely do not represent the sole factors regulating reproduction-related brain changes. For instance, although pregnancy comprised by far the most prolonged and endocrinologically extreme part of the period between the two MRI scans, we cannot with certainty exclude a contribution to our results of early postpartum factors in the time between birth and the Post acquisition. However, since the neural transformations were found to relate to pregnancy-specific processes and third-trimester pregnancy hormones but not to any of the measured postpartum factors or the duration of exposure to postpartum factors, these data suggest that the observed neurostructural adaptations primarily relate to the biological process and endocrine influxes of pregnancy. Future research tracking a wider spectrum of potential regulatory factors such as exercise, nutrition, genetic markers and environmental changes in detail in larger samples might reveal other factors at play.

Various limitations of our study need to be considered. While our project included a relatively large group of women, especially given the longitudinal and logistically challenging nature of the study, larger samples would be beneficial to further examine the functional implications of pregnancy-related neuroplasticity. We aimed to perform a very comprehensive study involving many measures in order to allow a rich characterization of the neural changes as well as their potential implications. However, the number of variables intrinsic to this type of study makes it difficult to adequately correct for the number of variables included in the correlation analyses without being overly stringent, although these analyses have now been corrected for multiple comparisons using a correlation-adjusted Bonferroni correction for the tests within the research question as well as for all prenatal or postnatal tests respectively. Another consideration regarding the functional implications is that, although we primarily interpret these as consequences of the brain changes, these functional changes in behaviour or physiology could also be hypothesized to trigger the observed neural modifications, or they could both arise from a related phenomenon.

Furthermore, it is not possible to define the cellular processes underlying the observed macroscopic changes based on our data. Considering the observed reductions in GM volume, neurodegenerative processes could potentially be hypothesized to play a role. However, the observed functional associations, the lack of changes in metabolite N-acetylaspartate‒a typical marker of neuro-axonal health and function^43^‒and the fact that aging and neurodegeneration are generally associated with reductions rather than increases in the coherence of the DMN^44^ argue against a neurodegenerative process. We have previously demonstrated a remarkable similarity between the morphometric properties of neural GM changes in pregnancy and adolescence^45^‒another transitional period associated with strong GM reductions and a myriad of behavioural, cognitive and social adaptations‒pointing to similar hormone-driven neurobiological mechanisms. Interestingly, maternal brain plasticity in non-human animals is known to often involve processes of neural ‘fine-tuning’ that optimize maternal behaviours^46^.

The observed correlation between changes in grey matter volume and myo-inositol, which represents a glial cell marker^47^, suggests that the observed alterations in grey matter may at least partially reflect changes in glial cells. However, the significance of this observation in terms of the cellular processes underlying these changes in grey matter remains to be elucidated.

Another limitation of this study is that, while we have tried to match our groups as well as possible, some differences between the groups are inherent to this type of pre-conception longitudinal approach, which entails that one group constitutes of women with a desire to get pregnant while the other group of women do not want to become mothers yet. Although we have matched our participants based on various characteristics and have tested for baseline differences in all measures, we cannot exclude that pre-existing differences between these groups could potentially play a role.

Finally, given the highly significant and consistent findings and the strong agreement of the observed findings with our previous results acquired in women from another country, our results provide support for a remarkable relative uniformity of pregnancy-related brain changes that extends across different cultural contexts. However, it should be noted that the women in both our studies were from European countries and were relatively highly educated, and we do not yet know with certainty whether these brain changes also extend to women from different educational, socio-economic or cultural backgrounds.

In conclusion, these findings indicate that pregnancy leads to selective and pronounced changes in neural architecture and neural network organization particularly affecting the Default Mode Network of the human brain, which may underlie transformations in the neural representation of the self when becoming a mother. Sex steroid hormones—primarily third-trimester estrogens—were identified as potential factors contributing to the observed neuroanatomical changes, while no associations were observed with other experiential or biological measures. Furthermore, our findings suggest that pregnancy-related neuroplasticity plays a role in psychological and physiological gestational maternal processes that help a woman to prepare for the arrival of her baby. In addition, the neural changes relate to mother-infant bonding and problems in the mother-infant relationship as well as further developments herein across the late postpartum period, suggesting a long-term impact for the mother and infant. These data provide key insights into the impact of becoming a mother on the human brain and point to pronounced changes in brain structure and function that promote gestational and postpartum maternal processes central to the establishment of the new mother-infant dyad.

## Methods

### Design and participants

This research was evaluated and approved by the Ethics Review Board of the Leiden University Medical Centre and complies with all relevant ethical regulations. We set up a pre-conception prospective cohort study in which nulliparous women were examined and followed throughout the next few years. Women with and without the intention to become pregnant in the near future took part in the initial session. Recruitment and data collection for these groups was initiated at the same time. The participants were recruited using advertisements, flyers (at local GPs and pharmacies) and by word of mouth. The final group allocation depended on the transition from nulliparity to primiparity during the course of this study. The final groups are referred to as the pregnant (PRG) and the control group (CTR).

Women trying to become pregnant were scanned in the early follicular phase of their menstrual cycle before a possible conception could have taken place or prior to the insemination or transfer if they were involved in an assisted fertility trajectory. In addition, for completeness, they performed an extra sensitive pregnancy test (10 mIU/ml) on the day of the pre-conception session. Only participants who had not experienced a previous pregnancy beyond the first trimester were included in the study. The main inclusion criterion for continuing in the study for participants as part of the PRG group was achieving pregnancy in the period following the first MRI session, while not becoming pregnant during the course of this study represented the main inclusion criterion for the CTR group.

The sessions took place pre-conception, during late pregnancy, the early postpartum period and the late postpartum period (further details regarding the timing are provided later in this section). All sessions involved MRI acquisitions except for the pregnancy session.

Hormone levels were measured throughout the women’s pregnancies (every 4 weeks and every 2 weeks in the final stages of pregnancy, starting in week 36, see section below for more information). Participants for this study were recruited from across the Netherlands, mostly from the city of Leiden and surrounding areas. The participants were invited to the Leiden University Medical Centre to take part in the project. Informed consent was obtained from all participants prior to their participation in the study. Participants received a monetary compensation for their participation.

Eighty-nine nulliparous women were recruited for the initial session, including 58 women who wanted to become mothers for the first time in the near future. From this sample, 1 woman dropped out of the study due to illness, 2 lost interest and 11 did not become pregnant within the first year after the pre-conception scan, rendering a total of 44 women for this group. Prior to the early postpartum session, 5 more women from this group dropped out of the study (3 lost interest, 2 did not reply to the investigators’ emails to schedule a session). In addition, one woman from the control group became pregnant after her final session in this group, and then participated in the MRI sessions of the PRG group. For completeness, the main analyses were repeated excluding this participant from the PRG group, which rendered practically identical results. This rendered a final PRG sample with completed longitudinal Pre and Post MRI sessions of 40 women. Of these women, 35 were able to also take part in the late pregnancy session in the third trimester (Prg session, week 36 of pregnancy). In addition, the primiparous mothers were asked to return for a final examination around one year after delivery. Of the 40 women, 3 could not participate in this late postpartum session as they were already pregnant with their second child, and 9 women did not participate due to loss of interest or because the final sessions of the project had to be cancelled due to COVID-19 restrictions, which led to an early stop of the data collection (missing at random). Therefore, 28 women took part in the late postpartum (Post + 1 y) session.

**Fig. 4:**
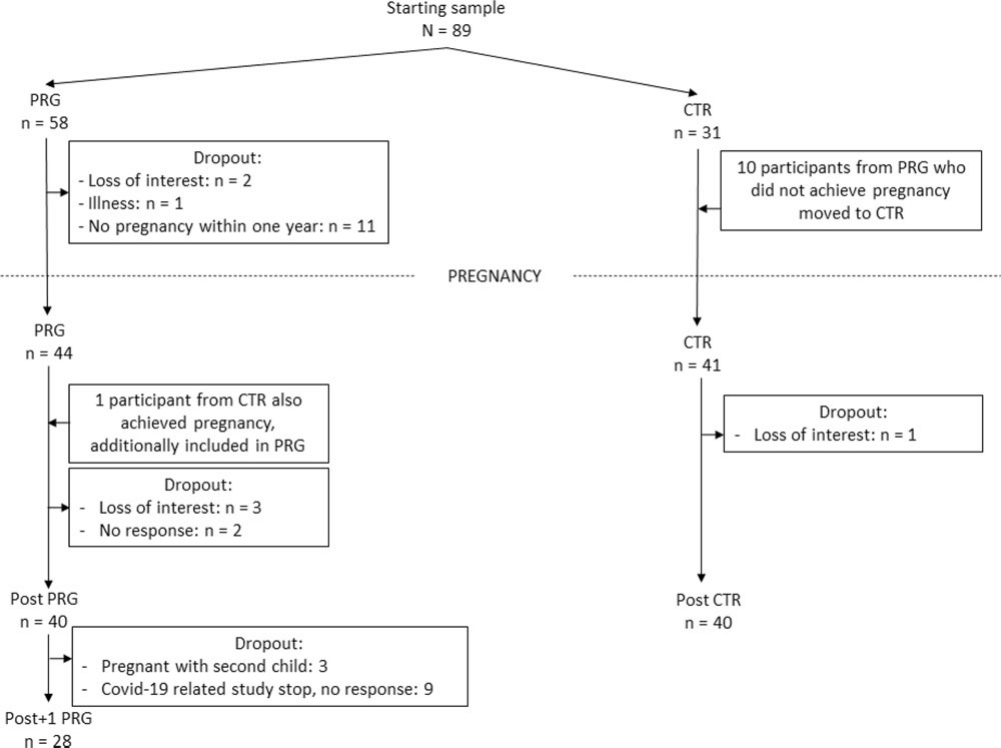
Schematic overview of subject attrition. Number of subjects participating in the first session in the PRG and CTR groups (the starting sample) and subject dropout for each of the sessions. PRG = women who became pregnant during this study, CTR = women who remained nulliparous during this study, Post = post-pregnancy session, Post+1 = late postpartum session.

In the CTR sample, 31 women with no intentions of becoming pregnant within the near future took part in the initial session, and 10 participants from the women with a pregnancy wish who did not become pregnant within the first year after the pre-conception session then took part in a Post session as part of the CTR sample. When scheduling the Post session, one participant dropped out due to loss of interest, rendering a total CTR sample with completed Pre and Post sessions of 40 women. For an overview of the group allocation and dropout, please see Fig. 4.

There were no statistically significant differences in age, level of education or Pre-to-Post time interval, between the groups (Supplementary Table 95). However, a trend was observed for a longer Pre-to-Post duration in the PRG group, which was driven by 3 participants who could initially not participate in the Post session but were scheduled after all at a later stage. Consequently, these participants had a time interval of more than 800 days between the Pre and Post session. Therefore, the main analyses were repeated excluding these 3 participants, which rendered highly similar results. For completeness, we also performed correlation analyses between these demographic variables and the observed brain changes, which rendered no significant results (see Supplementary Tables 96 and 97).

The Post session took place on average at 100.6 ± 70.8 days (mean ± s.d.) after parturition, and 95.4 ± 70.2 days when excluding the 3 delayed participants. The Post+1 y session took place on average 402.6 ± 64.8 days (mean ± s.d.) after parturition.

The majority of our PRG sample conceived via natural conception (37 women), while 3 women became pregnant by means of an assisted conception trajectory. Thirty-one women delivered their babies via vaginal birth, and 9 gave birth by means of a caesarean section. At the time of the POST session, 30 women were breastfeeding their infants and 10 used formula feeding. One woman delivered twins, and 39 women delivered singleton babies. Twenty-two women gave birth to a daughter, and 17 delivered a son.

Three of the women in the PRG group and 5 of the women in the CTR group had at some point in their lives experienced symptoms of depression or anxiety. One woman in the PRG group had previously suffered from an eating disorder, one had contracted meningitis as a baby, one had been diagnosed with ADHD and one suffered from an autonomic disorder (Harlequin syndrome), while one woman in the CTR group had suffered from facial pain and one had experienced a burn-out. For completeness, the main analyses in which any significant changes across pregnancy were observed (i.e. the analyses of grey matter volume, DMN temporal coherence and neural metabolites) were repeated including covariates for each of these conditions, rendering highly similar results.

### Materials and analyses

#### Anatomical MRI

##### Acquisitions

MRI acquisitions were performed on a 3-Tesla Philips MRI scanner. High-resolution 3D T1-weighted images were acquired in transverse orientation (Repetition Time (TR) = 9.8 ms; Echo Time (TE) = 4.6 ms; Flip Angle = 8°; voxel size = 0.875 × 0.875 × 1.20 mm^3^; Field of View= 178 × 224 × 168 mm). MRI scans were visually checked for quality control before data processing, but no images had to be excluded. Therefore, the analyses of the structural MRI data involved a total of 160 sessions for the analyses involving the Pre and Post sessions (40 subjects with complete Pre and Post datasets in each group), while analyses involving the Post+1 session involved 84 sessions in total (28 PRG participants who were scanned at baseline, early postpartum and late postpartum).

##### Longitudinal diffeomorphic modeling

The anatomical MRI images were processed in SPM12 (http://www.fil.ion.ucl.ac.uk/spm/), implemented in Matlab 7.8 (MathWorks), using the longitudinal symmetric diffeomorphic modeling pipeline^48^. The images of each participant were first processed using the longitudinal registration tool provided within this framework, which incorporates rigid-body registration, intensity inhomogeneity correction and nonlinear diffeomorphic registration in an interleaved fashion. Considering the bias associated with asymmetry in pairwise registration, this approach registers both time points to a within-subject average image. These midpoint average images were segmented into tissue classes using the unified segmentation algorithm^49^. The jacobian determinants resulting from the longitudinal registration were subsequently multiplied by each subject’s grey matter (GM) segment, creating maps of volumetric change in GM tissue. To bring these images into MNI space, the product images were normalized using DARTEL tools implemented in SPM12^50^ and smoothed with a 10-mm full-width half-maximum smoothing kernel. The individual smoothed GM volume difference maps were entered into general linear models.

A cross-sectional voxel-based morphometric approach was additionally applied to the baseline images to confirm the absence of pre-existing baseline differences between the PRG and CTR groups. This approach included a segmentation of the baseline images using the unified segmentation algorithm^49^, a DARTEL normalization of the grey matter (GM) segments^50^ and the application of a 10-mm full-width half-maximum smoothing kernel in SPM12. The Post + 1 year images were processed using the same longitudinal approach described above, rendering volume difference maps between the Post + 1 year images and the two other sessions.

##### Statistical analyses

General linear models in SPM were used to examine whether there are differences between the groups in the changes across sessions. The main comparisons involved comparing the maps of Pre-to-Post GM volume change between the groups (PRG and CTR) using two-sample *t* tests. Standard statistical tests in SPM were used, which are one-tailed. If a significant group difference was observed, we then proceeded to separately examine the changes within the groups separately using one-sample *t* tests in order to determine which changes were driving these group differences. To examine whether GM volumes within the regions affected by pregnancy underwent further changes across the first year postpartum relative to the Pre and early Post sessions, we performed one-sample *t* tests on the difference maps representing the volumetric changes between the Post+1 y session and the pre-conception baseline session and between the early and late postpartum sessions.

Effect sizes were extracted using the VBM8 toolbox (http://www.neuro.uni-jena.de/vbm/). To create images, the statistical maps were projected onto the PALS surface provided in Caret software (caret 5, http://brainvis.wustl.edu/wiki/index.php/Caret). Slice overlays were created using MRIcron (version 7 july 2012, http://www.mccauslandcenter.sc.edu/mricro/mricron/). The statistical maps were constructed by applying a stringent voxel-level threshold of *P* < 0.05 FWE-corrected across the whole brain.

##### Overlap quantification analyses

To objectively examine the localization of the observed anatomical changes, we computed the spatial correspondence between our structural changes and the cognitive components and resting state neural networks extracted from Yeo et al., who analyzed over 10.000 fMRI experiments to define a cognitive ontology of the human association cortex^11^ and resting state fMRI data of 1000 individuals to identify the organization of the human cerebral cortex at rest into intrinsic neural networks^12^. Overlap quantification analyses were additionally performed with the functional networks of Smith et al.^13^.

The overlap of our results with these functional maps was extracted by computing the intersections between each of these maps and the map of GM volume changes of pregnancy. The fraction of the observed intersection was then defined relative to the expected volume of the intersection based on a random distribution across the grey matter of the brain.

#### Diffusion-weighted MRI

##### Acquisitions

Diffusion-weighted imaging was performed in all participants. Two transverse DTI scans were acquired with the following parameter settings: 30 diffusion-weighted volumes with different non-collinear diffusion directions^51^ with b-factor 1000 s/mm2 and 5 diffusion-unweighted volumes (b-factor 0 s/mm2); parallel imaging sensitivity encoding for fast MRI (SENSE) factor = 3; Flip Angle 90°; 75 slices of 2 mm; no slice gap; acquisition matrix 128 × 98; reconstruction matrix 128 × 128; Field of View= 240 × 240 mm; TE = 69 ms; TR = 7315 ms; no cardiac gating; and scan duration = 271 s (542 s in total). The second DTI set had identical parameter settings as used for the first DTI set except that it was acquired with a reversed k-space readout direction enabling the removal of susceptibility artifacts during post-processing^52^. The diffusion-weighted scans were checked for quality before data processing, but no images had to be excluded. Therefore, the analyses of the diffusion-weighted MRI data involved a total of 160 sessions for the analyses involving the Pre and Post sessions (40 subjects with complete Pre and Post datasets in each group), while analyses involving the Post+1 session involved 84 sessions in total (28 PRG participants who were scanned at baseline, early postpartum and late postpartum).

##### Processing and analyses

The Diffusion-Weighted MRI scans were preprocessed using the FMRIB Software Library v6.0 (FSL 6.0)^53^. Susceptibility induced distortions were estimated and corrected using the topup tool^52^ and eddy was used to correct for current-induced distortions and subject movements^54^. Tensor fitting was then completed with dtifit.

DTI-TK software (version 2.3.3, http://dti-tk.sourceforge.net) was used for tensor-based registration of the scans. This package allows using the full diffusion tensor information to drive the registration, which leads to an improved alignment of white matter structures^55^.

Within-subject DTI templates were created using an iterative process of initial rigid registration of each subject’s longitudinal scans, followed by non-linear registration. Then a group template was generated by applying the same process to the intra-subject templates. The two transformations calculated for each scan were combined into a single deformation field, which was applied to the original tensor image to bring it to the group-wise template space. Maps of fractional anisotropy (FA), mean diffusivity (MD), axial diffusivity (AD), and radial diffusivity (RD) were then created for each registered diffusion tensor image in group-wise space.

Tract-based spatial statistics^56^ was subsequently applied. The mean FA image obtained from DTI-TK was thinned to create a mean FA skeleton representing the centres of all tracts common to the group. Each scan’s normalized FA data was then projected onto this skeleton, and this projection was then also applied to MD, RD and AD images. Difference images were calculated for each parameter (FA, MD, RD, AD) by subtracting each participant’s baseline skeletonized image from the follow-up one. Voxel-wise cross-subject statistics were applied for each parameter. Two sample *t* tests were performed to check for differences in the longitudinal evolution of FA, MD, RD and AD between the groups. Standard FSL statistical tests were applied (FSL randomize^57^), which are one-tailed. Results were corrected for multiple comparisons using a whole-brain FWE correction.

#### Proton nuclear magnetic resonance spectroscopy

##### Acquisitions

Magnetic Resonance Spectroscopy (MRS) was performed with single‐voxel point‐resolved spectroscopy (PRESS) localization (TR = 2000 ms; TE = 37 ms; 128 averages, 2 dummy scans, and 16 reference scans without water suppression). The volumes of interest (VOIs) were positioned in two regions that were found to undergo strong changes in brain structure in our previous study^10^. One was the precuneus/posterior cingulate cortex (PCC), centred between both hemispheres (Supplementary Figs. 11 and 12), which had a volume of 8 mL (20 × 20 × 20 mm^3^). This VOI will be referred to as the PCC VOI. The other VOI was positioned in the right superior temporal gyrus (Supplementary Fig. 11) and had a volume of 12 mL (20 × 30 × 20 mm^3^). The placement of both VOIs was chosen based on the clusters identified in Hoekzema et al.^10^. Shimming was performed with an automated second order projection-based algorithm.

Of the women included in the study, MRS data of the Pre and Post session were available of 39 women who became mothers during this study and 38 women of the CTR group, since the MRS acquisitions were omitted from one of the sessions of 3 participants due to a lack of time. In addition, one of the CTR women had to be excluded from the analyses because the spectroscopy VOI has been misplaced at the PRE session, rendering a total of 39 PRG (age at baseline (mean ± s.d.): 29.46 ± 3.49) and 37 CTR participants (age at baseline (mean ± s.d.): 29.22 ± 3.57) with complete Pre and Post datasets for the MRS analyses and 25 PRG participants for the Post+1 session.

##### Spectra quantifications and quality assessments

Metabolite concentrations were estimated with LCModel, (version 6.3-1 M), using a dataset containing 17 metabolites. For this study, we consider the major metabolites tNAA (N-acetylaspartate including contributions from N-acetylaspartylglutamate), tCr (creatine and phosphocreatine), Cho (phosphorylcholine and glycerophosphorylcholine), Glu (Glutamate), Ins (myo-Inositol). Concentrations were expressed using water scaling. Next, we corrected for partial volume contributions of GM, white matter and cerebrospinal fluid in the corresponding VOI, based on Sienax segmentation (FSL 5.0.10) of each subject’s 3DT1 images. Metabolite concentrations were calculated as described in the LCModel manual.

Spectral quality was examined based on the full-width half maximum (FWHM), signal-to-noise ratio (SNR), and the estimated Cramer-Rao lower bounds of each metabolite. Spectra with FHWM > 0.1 ppm (12 Hz) and/or SNR < 5 were considered poor quality. This led to exclusion of 75% of the STG spectra, and this region was left out from further analyses. All PCC spectra had high quality, with SNR mean±sd of 24.84 ± 1.86, FWHM 4.60 ± 0.61 Hz, and Cramer Rao lower bounds of metabolites well below 10%: tCr 2.13 ± 0.33%, tNAA 2.22 ± 0.41%, Cho 4.62 ± 0.51%, Ins 6.73 ± 0.74%, and Glu 7.95 ± 0.49%. No differences were observed in measures of spectral quality between sessions and groups.

##### Statistical analyses

The metabolite concentrations resulting from LCModel were analyzed in SPSS (version 25, https://www.ibm.com/products/spss-statistics). We ran repeated measures general linear models to assess whether there are differences between the groups in the changes in these metabolite concentrations across time. Normality was assessed using the Shapiro-Wilk test, and non-parametric tests (Mann Whitney U tests on the Post-Pre difference values) were performed in case of deviations from normality in any of the groups and sessions. Both the results from parametric and non-parametric tests are reported. The results were corrected for multiple comparisons (for the number of metabolites) using a Bonferroni correction^58^ (http://www.quantitativeskills.com/sisa/calculations/bonfer.htm). Since the Bonferroni correction procedure assumes independence between the tests, the correction threshold was adjusted according to the mean correlation (R) between the examined variables^58^.

#### Resting state functional MRI

##### Acquisitions

Functional MRI scans were acquired during rest from all participants. In order to minimize the possibility of subjects’ falling asleep, participants were instructed to fixate on a white cross presented in the middle of the screen. T2*-weighted whole-brain echo-planar images (EPIs) were acquired (139, including 2 dummy scans to allow for equilibration of T1 saturation effects) with the following acquisition parameters: TR = 2.2 s; TE = 30 ms; Flip Angle = 80°; Field of View = 220 × 220 × 111.65; voxel size = 2.75 × 2.75; 37 descending slices.

##### Preprocessing

The resting state fMRI images were preprocessed using DPARSF (version 4.5)^59^, involving slice time correction, realignment, and co-registration of the anatomical images to the mean functional images. The transformed anatomical images were then segmented^60^, and DARTEL^50^ was used to transform the images to MNI space. This was followed by the application of a 10 mm^3^ full-width half-maximum (FWHM) Gaussian kernel, which matches the recommended wide range smoothing kernel of 2–5 voxels of FWHM for multi-subject ICA^61^, to the space domain.

To account for head motion, we applied the Friston 24-parameter model^62^ and subjects with any frame-wise displacements (FD) exceeding 2 mm (for translations) or 2° (for rotations) or with a mean FD^63^ exceeding 0.2 in any of the sessions were excluded. Therefore, 4 participants from the CTR group had to be removed from the analyses involving the Pre and Post sessions, and 2 PRG participants from the analyses involving the Post+1 y session. This rendered a total sample for the analyses involving the Pre and Post sessions of 40 women in the PRG group (age at baseline (mean ± s.d.): 29.35 ± 3.51), and 36 women from the CTR group (age at baseline (mean ± s.d.): 29.22 ± 3.65).

##### Independent component analyses

Group spatial independent component analysis (ICA) was carried out using the Group ICA for fMRI Toolbox in Matlab (GIFT v4.0b, http://mialab.mrn.org/software/gift). Group ICA was applied using default options and the Infomax algorithm, involving the Pre and Post sessions of all subjects, and was carried out in three steps: Data reduction, ICA, and back-reconstruction. First, principal component analysis (PCA) was used to reduce the individual participants’ data, which were then concatenated, and followed by another PCA data reduction step. Next, group ICA was carried out using this reduced set of data. Finally, a back-reconstruction was performed of each participants’ time courses and spatial maps based on the output components and information for the PCA data reduction steps^64,65^. A low model order ICA of 20 components was used because this dimensionality is thought to be optimal for examining large-scale brain networks^13,66^. Components were selected through automated selection by spatial sorting with the components of Smith and colleagues^13^ who defined the major networks in the resting brain, using a cutoff value of R > 0.25. These rendered excellent correlation values, with a minimum of R = 0.41 and a maximum of R = 0.78 (see Supplementary Table 98). Given the strong overlap between our findings of structural change and the Default Mode Network, we were primarily interested in this network. Therefore, as an additional check, we also used the Default Mode Network component provided by GIFT in the spatial selection process, which rendered the same component (R = 0.63). The obtained networks are displayed in Supplementary Fig. 13. See Smith et al.^13^ for a description of the neural networks.

After determination of the neural networks, we additionally extracted the correlation between these networks using the FNC Toolbox (version 2.3, https://trendscenter.org/software/fnc/) for Matlab with a lag-shift algorithm. FNC calculates a constrained maximal lag correlation between each pair of networks by calculating Pearson’s correlations and constraining the lag between the time courses^65^. FNC was applied using default options, rendering correlations between the defined neural networks.

##### Statistical analyses

Within-network coherence was examined for each of the defined neural networks in SPM12 using general linear models. To examine whether there are differences between the groups in the change in network coherence across sessions, we tested for group (PRG, CTR)* session (Pre, Post) interaction effects. When significant results were observed, these were followed by paired samples *t* tests. For completeness, to enhance the consistency of the statistical approach with the analyses of the anatomical data, we additionally performed 2 sample *t* tests on the Post-Pre difference images, which rendered the same results. To restrict results to the core regions pertaining to the network, a mask of the mean component (imcalc > 0.5) for the respective network was applied to the analyses as an explicit mask. Given the strong overlap between the structural changes and the DMN, we were primarily interested in the within-network and between-network connectivity of the DMN, but the results for all neural networks are reported.

Changes across the postpartum period were examined by means of paired samples *t* tests involving the Post+1 y follow-up session and the two other sessions. For completeness, the Pre sessions were additionally compared to check for baseline differences. Results were considered significant at an FWE-corrected statistical threshold of *P* < 0.05.

Between-network correlations were examined in SPSS (version 25, https://www.ibm.com/products/spss-statistics), using repeated measures General Linear Models. To examine whether there are changes in the connectivity between networks between the pre-pregnancy and post-pregnancy session, we tested for interaction effects between group (PRG/CTR) and session (Pre/Post). In addition, data from the Post+1 session was used to investigate whether there are changes across the postpartum period in between-network connectivity by means of paired samples *t* tests. Results were considered significant at a statistical threshold of *P* < 0.05 corrected for multiple comparisons using a correlation-adjusted Bonferroni correction (http://www.quantitativeskills.com/sisa/calculations/bonfer.htm)^58^.

Effect sizes were extracted using the VBM8 toolbox (http://www.neuro.uni-jena.de/vbm/). To create images, the statistical maps were projected onto the PALS surface provided in Caret software (http://brainvis.wustl.edu/wiki/index.php/Caret). Slice overlays were created using MRIcron (http://www.mccauslandcenter.sc.edu/mricro/mricron/).

#### Physiological data

##### Acquisition

Physiological data were acquired using a Biopac MP150 system. Electrodes to measure the participants’ galvanic skin conductance response (SCR) were placed on the non-dominant hand. The participants’ heart rate responses were measured by means of electrocardiographic (ECG) electrodes placed on the chest and abdomen. Facial electromyography data have also been acquired within the framework of this project, but these were not included in analyses of the current data. Stimulus and response onset markers were conveyed from E-Prime (version 2.0, https://pstnet.com/products/e-prime/) via a parallel port and saved into an event marker channel. Data were stored using AcqKnowledge software (Acqknowledge 5.0, BIOPAC Systems Inc., Goleta, CA).

To acquire an indication of the women’s physiological reactions to infant cues, movies representing crying and laughing infants were shown to the participants on a computer screen using E-prime while their physiological reactions to the depicted stimuli were recorded. Seven 5.7 s movies of laughing babies and seven 5.7 s movies of crying babies were used, each presented to the participants 3 times in random order with an inter-trial interval of 10 seconds, followed by a question about the shown movie after the final presentation. Responses were averaged across the repeated presentations of the stimuli.

Thirty-five of the PRG women participated in the pregnancy session. However, for 3 subjects, a technical issue prevented the Biopac system from working accurately (*N* = 2) or from transmitting the markers to the event marker channel (*N* = 1). For 2 subjects, the placed electrodes did not remain firmly in place throughout the session. Therefore, reliable physiological data was acquired from 30 of the pregnant women (age at baseline (mean ± s.d.): 29.10 ± 3.38). For the skin conductance data, 2 subjects had to be removed from the laughing baby condition to correct for outliers in the data (see below) (age at baseline (mean ± s.d.): 29.04 ± 3.49).

##### Processing and analyses

All physiological data were preprocessed with BrainVision Analyzer software (Brain Vision Analyzer 2.2, Brain Products Inc., Gilching, Germany). Regarding the electrocardiographic (ECG) data, after filtering the data (10 Hz low cutoff filter), the R peaks were detected using in-house code for a Brain Vision Analyzer macro. Invalid inter-beat-intervals (IBIs) were automatically marked and adjacent R peaks were manually corrected. IBIs were then converted to a new timeseries (channel) using the amplitude of each IBI as amplitude, and applying linear interpolation between the discrete IBIs. ECG and skin conductance response data were averaged across trials separately for each condition. The data were baseline-corrected by subtracting the mean activity from 1000 to 0 ms prior to stimulus onset from the activity in the rest of the bins. The average value across a time window spanning the entire stimulus duration (0 to 5700 ms) was exported for statistical analyses.

Outliers were removed using is_extreme in RStudio (Rstudio 1.4.1717). The outliers included outliers in the dependent variables, but also outliers in the proportion of the segments retained after the pre-processing of the data (i.e., if too few of the measurements from a specific condition could be used after filtering the data, the value for that specific participant in that physiology was excluded). This led to the exclusion of 2 participants for the skin conductance response data for the laughing babies condition.

The interval between RR peaks was extracted to examine the participants’ heart rate interval in response to the presented stimuli (mean ± sd: ECG: cry: 0.616 ± 2.438, happy: 0.447 ± 2.473. *N* = 30) and the galvanic skin responses to the stimuli were recorded to measure the participants’ electrodermal activity in reaction to these stimuli (mean ± sd: SCR: cry: 0.024 ± 0.031. *N* = 30. happy: −0.004 ± 0.013. *N* = 28). The acquired data were included in correlation analyses (see section Correlation Analyses) to check for potential associations with the observed neural changes.

#### Hormone sampling

Every 4 weeks throughout their pregnancy and every 2 weeks near the end of pregnancy, women collected their first morning urine (pregnancy weeks 8, 12, 16, 20, 24, 28, 32, 36, 38, 40). Urine samples were saved in 10 mL polypropylene tubes and stored in a −20 freezer. Hormones were sampled at the Technical University of Dresden, using a high-throughput liquid chromatography-tandem mass spectrometry (LC-MS/MS) assay with atmospheric pressure chemical ionization coupled with online solid phase extraction^67^. This assay allowed for the simultaneous identification of Estradiol (pg/ml), Estriol (pg/ml), Progesterone (pg/ml), and Cortisol (nmol/L) from our samples. The intra- and inter-assay Coefficients of Variability for all steroid hormones measured were less than 12%.

To correct for urine concentration, levels of creatinine (mg/dl) were determined with a kinetic colorimetric test (Jaffé method) which is retraceable to the isotope-dilution mass spectrometry (IDMS) reference method. Intra- and inter-assay Coefficients of Variability were below 3%. All measures were corrected for urine concentration in the sample by adjusting for creatinine levels.

In addition, to examine osmotic changes, urine osmolality (mosmol/kg) was determined from all samples using an automated cryoscopy by Axonlab Arkray. The intra- and inter-assay Coefficients of Variability for these measures were 0.12% and 1.65% respectively.

Of the 40 PRG subjects, 37 women collected urine samples throughout their pregnancy. Tubes were missing (either not collected or lost during sampling (2 tubes)) for 7 subjects in week 8, 4 in week 12, 3 in week 1, 2 in week 20, 1 in week 24, 4 in week 32, 3 in week 36, 11 in week 38. In week 40 of pregnancy, many women had already given birth, and samples were thus missing from many (28) participants. Therefore, measures from week 40 of pregnancy were not used for correlation analyses given the small number of available samples. In addition, estriol could not be detected in 1 sample in week 8 and 1 sample in week 12, estradiol could not be detected in 2 samples in week 12, 1 sample in week 16, 20 and 40, and progesterone was not detected in 1 sample in week 32. Mean hormone levels per time point are depicted in Supplementary Figs. 14–17.

Correlation analyses were performed on the averaged hormone levels across the women’s pregnancy, and were followed up by correlations for every separate sampling point if a significant correlation was observed.

#### Scales and questionnaires

Antenatal maternal-foetal attachment was examined using the Prenatal Attachment Inventory^68,69^ (mean ± sd. Anticipation: 16.34 ± 2.68, Differentiation: 18.25 ± 2.98, Interaction: 19.40 ± 2.74. N = 35). In addition, the Maternal Antenatal Attachment Scale (MAAS)^70^ was applied to measure the quality of attachment and the intensity of preoccupation of a mother with her unborn child (mean ± sd: Quality of Attachment: 45.46 ± 3.49, Intensity of Preoccupation: 29.83 ± 3.83. N = 35).

To examine whether changes in mother’s brain are related to preparational activities during pregnancy, also referred to as nesting behaviour, we used the Nesting Questionnaire^18^, which consists of 4 subscales centred around the topic of space preparation (subscales cleaning/mental focus, and energy burst) and social selectivity (subscales familiarity preference and novelty aversion) (mean ± sd: Cleaning: 4.43 ± 1.24, Energy Burst: 0.60 ± 0.81, Familiarity Preference: 3.34 ± 1.39, Novelty Aversion: 2.26 ± 0.74. *N* = 35).

To acquire an indication of the postpartum bonding between a mother and her child, we used the Maternal Postnatal Attachment Scale^71^, measured during the late postpartum session as well as the early postpartum period. From this scale, three scores were extracted; the Quality of Attachment, the Absence of Hostility and the Pleasure in Interaction (mean ± sd: Late postpartum: MPAS total: 81.38 ± 7.08, Quality of Attachment: 41.64 ± 2.50, Absence of Hostility: 19.41 ± 2.76, Pleasure in Interaction: 20.32 ± 3.55. *N* = 28. Early postpartum: MPAS total: 81.28 ± 6.52, Quality of Attachment: 40.28 ± 3.83, Absence of Hostility: 19.97 ± 1.91, Pleasure in Interaction: 21.03 ± 2.39. N = 39).

To examine whether there were signs of impaired bonding, infant rejection or abuse, we applied the Postpartum Bonding Questionnaire (PBQ), which renders a total score as well as scores for Impaired Bonding, Rejection and Pathological Anger, Infant-focused Anxiety and the Risk of Abuse^72^ (mean ± sd: Late postpartum: PBQ Total: 10 ± 7.16, Impaired Bonding: 6.18 ± 4.15, Infant Rejection: 2.07 ± 2.49, Anxiety about Care: 1.54 ± 1.45, Risk of Abuse: 0.21 ± 0.49. *N* = 28. Early postpartum: PBQ Total: 11.23 ± 7.58, Impaired Bonding: 6.59 ± 4.02, Infant Rejection: 2.38 ± 2.82, Anxiety about Care: 2.16 ± 1.48, Risk of Abuse: 0.10 ± 0.31. *N* = 39). Measures that were not available in Dutch were translated by an English-Dutch translator and then back-translated by a native Dutch speaker to double-check for accuracy.

To measure psychological distress in women transitioning to motherhood during pregnancy and the postpartum period, the K10 questionnaire was applied^73^ (mean ± sd: K10: pregnancy: 7.63 ± 4.47. *N* = 35. postpartum: 7.21 ± 6.14. *N* = 39). In addition, the mothers were retrospectively asked about the stress levels they experienced during their pregnancy and in the first postpartum year as a result of various factors (worries about their foetus or baby, work, physical complaints, emotional distress, or difficulties in their social environment) on a 5-point Likert scale, which were averaged to obtain an indication of overall distress during pregnancy and the first postpartum period (mean ± sd: Pregnancy: subjective stress levels: 26.38 ± 14.60. *N* = 28. Postpartum: subjective stress levels:26.73 ± 19.09. *N* = 28).

To obtain an indication of the women’s sleep during pregnancy and the postpartum period, the participants were asked to keep track of their sleep duration and number of sleep disruptions. At home, by means of online questionnaires, participants were asked to provide the number of hours and number of times their sleep was disrupted during the week prior to the sessions (mean ± sd: pregnancy: Mean number of hours: 7.74 ± 1.46. Mean number of sleep disruptions: 2.83 ± 1.72. *N* = 35. postpartum: Mean number of hours: 6.70 ± 0.91. Mean number of sleep disruptions:2.255 ± 1.32. *N* = 40). Based on the information provided by the participants, the average number of hours slept during the last week and the average number of times they woke up during the last week prior to the session were included in correlation analyses. In addition, the women were asked to report changes in sleep across their pregnancy and early postpartum period in terms of the average number of hours and the number of sleep disruptions within these periods. Based on this information, we computed an indication of the total hours of sleep and the total number of sleep disruptions for their pregnancy (until 36 weeks of pregnancy) and the postpartum period (until the Post session), which were then averaged across the number of days included in that time period (mean ± sd: pregnancy: Mean number of hours: 7.99 ± 0.81. Mean number of sleep disruptions: 2.14 ± 0.96. *N* = 35. postpartum: Mean number of hours: 6.89 ± 1.17. *N* = 35. Mean number of sleep disruptions:2.34 ± 1.30. *N* = 36).

To further examine the contribution of postpartum factors to the observed changes, correlations were additionally performed with the time (in days) between the delivery and the Post session, which reflects the fraction of the postpartum period included in the pre-post time interval.

In order to investigate the potential impact of breastfeeding and type of delivery on a woman’s brain changes, the participants were asked about the type of delivery (vaginal delivery: N = 31, caesarean section: *N* = 9) and their breastfeeding status (breastfeeding: *N* = 30, not breastfeeding: *N* = 10). The women breastfeeding their babies were additionally asked for the number of feedings per 24 hours (mean ± sd: 7.03 ± 1.96. *N* = 30). Finally, we also collected data on the total duration (in months) of breastfeeding until the Post+1 y session (mean ± sd: 5.71 ± .28. *N* = 25).

#### Functional implications analyses

To examine the potential functional implications of pregnancy-related changes in a woman’s brain, we investigated the possible relation of various gestational and postpartum measures with the observed brain changes by means of correlation analyses. We were primarily interested in investigating whether pregnancy-related neural changes are associated with the stimulation of gestational maternal processes that facilitate the preparation for parturition and motherhood. Therefore, we investigated whether the neural changes related to the pregnant women’s (i) maternal-foetal attachment, (ii) physiological reactions to infant cues, (iii) nesting behaviour.

Finally, we were additionally interested whether these neural changes related to postpartum measures of (i) mother-infant bonding, and (ii) impairments in the mother-infant relationship.

To examine whether these functional measures were associated with the observed neural changes, we performed correlation analyses for each of the modalities in which clear changes across pregnancy were observed (i.e. changes in grey matter structure extracted from the anatomical data and changes in Default Mode Network coherence extracted from the resting state fMRI data) in the women in the PRG group. Correlation analyses were performed for the structural measures (grey matter structure) and functional measures (DMN coherence) separately, since these concern different modalities that are not necessarily related and could be associated with distinct functional implications.

Correlation analyses with changes in neural activity were performed using betavalues extracted with Marsbar 0.44 (http://marsbar.sourceforge.net) from the SPM models in SPSS (version 25, https://www.ibm.com/products/spss-statistics) using two-tailed Pearson’s correlations. The data were checked for linearity and extreme outliers, and normality was examined using Shapiro-Wilk tests. A non-parametric Spearman’s test was applied in case of any deviations. Both results of parametric and non-parametric tests are reported.

Since the changes in brain structure comprised a widespread pattern of changes across the brain rather than a focal change affecting a specific brain area, the regression analyses with changes in brain structure were performed in PRoNTo (version 2.1.3, http://www.mlnl.cs.ucl.ac.uk/pronto/)^74^, using multivariate regression analyses. More specifically, kernel ridge regression analyses with leave-one-out cross-validation were used. Permutation testing was used to compute the sampling distribution and generate P values (*N*permutations = 10,000; *P* < 0.05).

To examine whether pregnancy-related neuroplasticity was associated with functional changes that facilitate the preparation for parturition and motherhood, correlation analyses were performed for the changes in grey matter volume and DMN coherence within the following domains: (i) maternal-foetal attachment (the PAI and MAAS; 5 measures), the physiological response to infant cues (heart rate and skin conductance response to crying and laughing infants; 4 measures), (iii) and nesting behaviour (the nesting questionnaire; 4 measures).

We additionally wanted to test whether these changes were associated with: (i) postpartum bonding and (ii) impairments in the mother-infant relationship. Since we had acquired measures both in the early postpartum and late postpartum period, we first tested for associations with the total scores for bonding (acquired with the MPAS) and bonding impairments (acquired with the PBQ) for each of these time points. If a significant effect or trend was observed, we proceeded to perform correlation analyses with the subscores for that time point (MPAS; 3 measures, PBQ: 4 measures). Based on the observed results, as a supplementary analysis, we additionally performed an analysis of the Post+1 y—Post measures of these scales to test for associations with changes across the postpartum period.

The correlation results were considered significant at a statistical threshold of *P* < 0.05 corrected for multiple comparisons for each modality using a correlation-adjusted Bonferroni correction^58^ (http://www.quantitativeskills.com/sisa/calculations/bonfer.htm) for the number of applied tests per research question (reported with a * in the results). In addition, a Bonferroni correction across all prenatal measures (i.e. the PAI, MAAS, Nesting questionnaire, all physiological measures) or across all performed postnatal measures for that modality (i.e. the total scores of the Post and Post+1 y sessions, the Post+1 y subscores, the Post+1 y—Post totals and subscores of the MPAS and PBQ) respectively was additionally applied, and for each measure is indicated (with **) whether the result also survives this correction.

#### Contributing factors analyses

We hypothesized that pregnancy hormones, primarily sex steroid hormones, represent the primary factors triggering or regulating pregnancy-related neuroplasticity. Therefore, correlation analyses (described above) were performed with measures representing the women’s exposure to several sex steroid hormones (estradiol, estriol, progesterone) and cortisol throughout pregnancy (described in the Hormones section). A correlation-adjusted Bonferroni correction^58^ was applied to correct for multiple comparisons (the number of hormones).

Furthermore, to check whether we could find indications that other factors were also significantly contributing to the observed neural changes, we performed correlation analyses with various other measures. The following domains were investigated: Stress (subjective stress during pregnancy and postpartum, K10 during pregnancy and postpartum; 4 measures), osmolality (4 measures), sleep (average hours of sleep and number of sleep disruptions the week prior to the pregnancy and postpartum session and an indication of the hours and sleep disruptions during the first 36 weeks of pregnancy and early postpartum period; 8 measures), duration of exposure to postpartum factors (time between delivery and the Post session; 1 measure), breastfeeding (the number of feedings in breastfeeding women in the early postpartum period; 1 measure). The relation between postpartum neural changes and the total duration of breastfeeding until the Post+1 session was additionally investigated as a supplementary analysis. Since these measures are likely to render very subtle effects and are included in the study as factors to control for rather than factors of interest (which would reveal experience-dependent effects of e.g. stress and sleep loss rather than hormone-driven pregnancy-specific changes), we did not apply a Bonferroni correction to these analyses of alternative contributing factors. For completeness, the main models were also re-run in SPM (using the same approach outlined in the sections above) for the measures regarding sleep, stress, and breastfeeding while including these variables as confounding factors. This allowed us to examine whether the observed brain changes could still be observed when correcting for these factors. Potential effects of the type of delivery (vaginal versus caesarean section) and breastfeeding (breastfeeding versus not breastfeeding) were examined by comparing the brain changes in these groups in SPM.

### Reporting summary

Further information on research design is available in the Nature Research Reporting Summary linked to this article.

## Supplementary information

Supplementary Information

Reporting Summary

## Data Availability

Source data are provided with this paper: For each of the plots, Source Data files have been made available. The raw MRI data along with the used correlation variables and group/demographic information for the participants who have provided permission to share their data (13 women in the Ctr group and 14 women in the Prg group) are provided in the Open Science Framework depository. These data have been deposited in the Open Science Framework depository under the following 10.17605/OSF.IO/5MT8Z. The deposited data are available open access. Source data are provided with this paper.

## Source data

Source Data

## Code availability

Analyses are based on pipelines integrated within the software. No custom code is used that is central to the conclusions.
