# Supplementary material for: Mapping the effects of pregnancy on resting state brain activity, white matter microstructure, neural metabolite concentrations and grey matter architecture
Source: Nat Commun. 2022 Nov 22;13:6931. doi: 10.1038/s41467-022-33884-8 (PMC9681770; doi:10.1038/s41467-022-33884-8)
Supplement: Supplementary file 1 — Supplementary Information [file 41467_2022_33884_MOESM1_ESM.pdf]

Supplementary Material

Supplementary Table 1. Changes in grey matter volume (PRE to POST).

Contrasts	Regions	MNI coordinates			*T*	*p* (FWE-corrected)	Cluster Size (mm^3)
		x	*y*	*z*			
PRG > CTR	-						
CTR > PRG	R Superior Temporal Sulcus, R Middle, Inferior, Superior Temporal Gyrus, R Fusiform	63	-35	-18	10.19	<0.001	5907
		62	-24	-17	9.72	<0.001	
		59	-44	-12	9.37	<0.001	
		53	-3	-36	6.02	0.005	74
		48	11	-38	5.67	0.019	26
	L Superior Temporal Sulcus, L Middle, Inferior, Superior Temporal Gyrus, L Fusiform, L Temporo-Parietal Junction, L Angular Gyrus, L Inferior, Superior Parietal Lobule, L Supramarginal Gyrus, L Lingual Gyrus, Inferior, Middle, Superior Occipital Gyrus, L Parahippocampal Gyrus, L Postcentral Gyrus	-51	-54	42	10.01	<0.001	14984
		-42	-68	38	9.28	<0.001	
		-60	-47	-9	8.90	<0.001	
	LR Superior Medial Frontal Cortex, LR Inferior, Middle, Superior Frontal Gyrus, LR Orbitofrontal Cortex, LR Supplementary Motor Area, LR Precentral Gyrus, LR Anterior Cingulate Cortex, LR Middle Cingulate Cortex, LR Gyrus Rectus, LR Olfactory Gyrus, LR Temporal Pole, LR Insula	-26	27	59	9.91	<0.001	51707
		18	50	42	9.58	<0.001	
		12	62	33	9.39	<0.001	
		39	27	-12	6.66	<0.001	673
		33	42	-15	6.20	0.003	247
		24	48	-18	5.73	0.015	
		27	33	-17	5.68	0.018	
		-56	14	-27	5.58	0.026	19
	R Temporo-Parietal Junction, R Angular Gyrus, R Inferior, Superior Parietal Lobule, R Supramarginal Gyrus, R Lingual Gyrus, R Fusiform, R Middle, Superior Occipital Gyrus, R Postcentral Gyrus	39	-68	39	9.30	<0.001	4562
		48	-60	33	9.30	<0.001	
		41	-62	45	8.90	<0.001	
		32	-48	-11	5.50	0.034	13
	LR Precuneus, LR Posterior Cingulate Cortex, LR Middle Cingulate Cortex, LR Calcarine Sulcus, LR Cuneus	-8	-39	35	8.41	<0.001	3982
		-11	-54	15	7.78	<0.001	
		5	-35	36	7.48	<0.001	
		18	-48	3	6.02	0.005	47
	L Hippocampus	-27	-18	-18	6.37	0.001	58
	R Hippocampus, R Parahippocampal Gyrus	27	-18	-18	6.46	0.001	54
	L Caudate	-8	8	9	6.84	<0.001	219
	R Caudate	11	5	14	5.55	0.029	6
	LR Posterior Cerebellum, Vermis	-33	-65	-27	6.53	0.001	229
		36	-60	-29	6.47	0.001	118
		3	-54	-53	6.35	0.002	325
		-6	-56	-50	6.13	0.003	
		38	-63	-42	6.25	0.002	95
		2	-47	-17	6.28	0.002	113

Note. Results of the main model comparing grey matter volume changes between the Pre and Post sessions in the women who were pregnant between sessions in comparison to the nulliparous control group. Statistics are extracted from two-sample t-tests performed within the framework of an SPM12 General Linear Model and are one-sided (as is standard in SPM). Results are reported at a statistical threshold of p<0.05 FWE-corrected. P-value at peak voxel (whole-brain FWE corrected) is reported. PRG = nulliparous women who were pregnant between sessions, CTR = nulliparous women who were not pregnant between sessions, L = left, R = right.

Supplementary Table 2. A further specification of the CTR>PRG findings.

Contrasts	Regions	MNI coordinates			T	p (FWE-corrected)	Cluster Size (mm³)
		x	y	z			
CTR Increases	-						
PRG Decreases	LR Superior, Middle, Inferior Frontal Gyrus, LR Superior Medial Frontal Cortex, LR Superior, LR Orbitofrontal Cortex, Middle, Inferior Temporal Gyrus, LR Superior Temporal Sulcus, LR Temporal Pole, LR Precuneus, LR Cuneus, LR Superior, Middle, Inferior Parietal Lobule, Anterior, Middle, Posterior Cingulate Cortex, LR Precentral Gyrus, LR Fusiform, LR Angular Gyrus, LR Supramarginal Gyrus, LR Temporo-Parietal Junction, LR Insula, LR Anterior, Posterior Cerebellum, Superior, Middle, Inferior Occipital Gyrus, LR Lingual Gyrus, LR Caudate, LR Precentral Gyrus, LR Putamen, LR Calcarine Sulcus, LR Gyrus Rectus, LR Hippocampus, LR Parahippocampal Gyrus, LR Thalamus, LR Postcentral Gyrus, Vermis, LR Olfactory Gyrus, LR Pallidum, LR Paracentral Gyrus	-60	-46	-8	16.22	<0.001	3354972
		60	-22	-16	15.69	<0.001	
		-63	-30	-14	15.10	<0.001	
	L Paracentral Lobule	-4	-21	81	5.55	0.029	24
	R Postcentral Gyrus	16	-32	81	5.00	0.041	5

Note. Following up on the significant group differences obtained in the PRG>CTR contrast reported in Supplementary Table 1, this table reports the results for contrasts representing the increases in the CTR group and decreases in the PRG group between the pre-conception and post-pregnancy sessions, allowing us to examine whether the results observed in the CTR>PRG contrast reflect grey matter volume increases in the CTR group or grey matter volume decreases in the PRG group. Statistics are extracted from one-sample t-tests performed within the framework of an SPM12 General Linear Model and are one-sided (as is standard in SPM). Results are reported at a statistical threshold of $p<0.05$ FWE-corrected. P-value at peak voxel (whole-brain FWE corrected) is reported. PRG = nulliparous women who were pregnant between sessions, CTR = nulliparous women who were not pregnant between sessions, L = left, R = right.

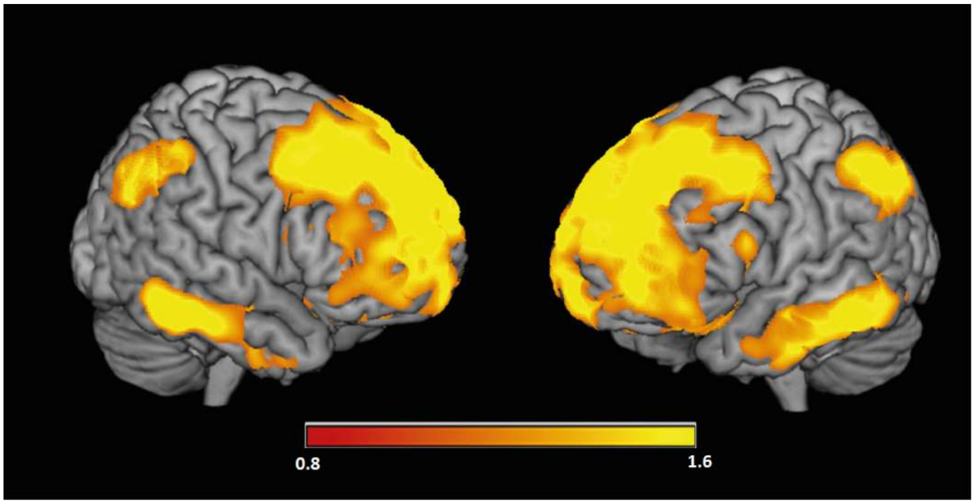

Supplementary Figure 1. Effect sizes (Cohen's d) for the PRE to POST changes in GM volume. Effect sizes are presented for the changes between sessions in the women who were pregnant between sessions in comparison to the control women. All depicted effect sizes correspond to large effect sizes (Cohen's d>0.8). Effect sizes were extracted using the VBM8 toolbox (http://www.neuro.uni-jena.de/vbm/) and plotted in mricron (https://www.nitrc.org/projects/mricron). N pregnant group = 40, N control group = 40.

Supplementary Table 3. Changes in grey matter volume (PRE to POST) excluding the participants with the long time interval between sessions.

Contrasts	Regions	MNI coordinates *x*	MNI coordinates *y*	MNI coordinates *z*	*T*	*p* (FWE-corrected)	Cluster Size (mm^3)
PRG > CTR	-						
CTR > PRG	L Superior, Middle, Inferior Temporal Gyrus, L Superior Temporal Sulcus, L Temporal Pole, L Fusiform, L Angular Gyrus, L Supramarginal Gyrus, L Temporo-Parietal Junction, L Superior, Inferior Parietal Lobule, L Lingual Gyrus, L Superior, Middle, Inferior Occipital Gyrus, L Parahippocampal Gyrus, L Putamen, L Postcentral Gyrus	-51	-54	42	9.91	<0.001	14082
		-42	-68	38	8.92	<0.001	
		-64	-30	-15	8.78	<0.001	
		-26	-16	3	5.64	0.022	14
	R Superior, Middle, Inferior Temporal Gyrus, R Superior Temporal Sulcus, R Fusiform	63	-34	-18	9.84	<0.001	5362
		62	-24	-16	9.55	<0.001	
		54	-36	2	7.36	<0.001	
		52	-3	-36	5.75	0.015	30
	LR Superior Medial Frontal Cortex, LR Superior, Middle, Inferior Frontal Gyrus, LR Orbitofrontal Cortex, LR Supplementary Motor Area, LR Precentral Gyrus, LR Anterior, Middle Cingulate Cortex, LR Gyrus Rectus, LR Olfactory Gyrus, L Temporal Pole, LR Insula, LR Precentral Gyrus, LR Olfactory	-26	27	58	9.83	<0.001	49081
		20	50	40	9.23	<0.001	
		10	62	33	9.10	<0.001	
		40	27	-12	6.49	0.001	579
		33	42	-16	5.86	0.010	65
		24	48	-18	5.58	0.027	18
		27	33	-16	5.46	0.041	2
	R Angular Gyrus, R Supramarginal Gyrus, R Temporo-Parietal Junction, R Superior, Inferior Parietal Lobule, R Temporal Pole, R Middle, Superior Occipital Gyrus, R Postcentral Gyrus, R Superior, Inferior Temporal Gyrus, R Superior Temporal Sulcus, R Fusiform, R Lingual Gyrus	48	-60	33	8.96	<0.001	4325
		39	-68	39	8.94	<0.001	
		40	-62	45	8.57	<0.001	
		48	10	-38	5.83	0.011	43
		32	-48	-10	5.46	0.042	8
	LR Precuneus, LR Posterior, Middle Cingulate Cortex, LR Calcarine Sulcus, LR Cuneus, R Lingual Gyrus	-8	-40	36	8.08	<0.001	3726
		4	-33	36	7.48	<0.001	
		-10	-54	15	7.45	<0.001	
		18	-46	3	6.04	0.005	46
	R Hippocampus, R Parahippocampal Gyrus	27	-20	-16	6.97	<0.001	76
	L Hippocampus	-27	-18	-18	6.23	0.003	49
	R Caudate	10	4	14	5.79	0.013	19
	L Caudate	-8	6	10	6.85	<0.001	233
	LR Posterior Cerebellum, Vermis	34	-60	-27	6.67	<0.001	153
		3	-54	-52	6.38	0.002	397
		-6	-56	-50	6.37	0.002	
		-32	-64	-27	6.48	0.001	220
		2	-46	-16	6.30	0.002	121
		38	-62	-42	6.12	0.004	68
		22	-72	-27	5.41	0.050	1

Note. Main model comparing grey matter volume changes between the Pre and Post sessions in the PRG and CTR groups, excluding the 3 women who initially did not participate in the Post session and therefore had a delayed time interval (>800 days) between the sessions (leaving a PRG sample size of N = 37 Statistics are extracted from two-sample t-tests performed within the framework of an SPM12 General Linear Model and are one-sided (as is standard in SPM). Results are reported at a statistical threshold of p<0.05 FWE-corrected. P-value at peak voxel (whole-brain FWE corrected) is reported. PRG = nulliparous women who were pregnant between sessions, CTR = nulliparous women who were not pregnant between sessions, L = left, R = right.

Supplementary Table 4. Changes in grey matter volume (PRE to POST) corrected for medical history.

Contrasts	Regions	MNI coordinates x	y	z	*T*	*p* (FWE-corrected)	Cluster Size (mm³)
PRG > CTR	-						
CTR > PRG	LR Superior, Middle, Inferior Frontal Gyrus, LR Medial Superior Frontal Cortex, LR Orbitofrontal Cortex, LR Gyrus Rectus, LR Insula, LR Supplementary Motor Area, LR Anterior, Middle Cingulate Cortex, LR Temporal Pole, LR Precentral Gyrus, LR Olfactory Gyrus	-24	30	57	10.95	<0.001	60680
		14	62	33	9.63	<0.001	
		18	50	42	9.60	<0.001	
	L Superior, Middle, Inferior Temporal Gyrus, L Superior Temporal Sulcus, L Temporal Pole, L Fusiform, L Angular Gyrus, L Supramarginal Gyrus, L Temporo-Parietal Junction, L Lingual Gyrus, L Inferior, Middle, Superior Occipital Gyrus, L Parahippocampal Gyrus,	-62	-46	-8	9.89	<0.001	14987
		-51	-54	44	9.52	<0.001	
		-64	-30	-14	9.49	<0.001	
		-54	15	-28	5.57	0.034	6
	R Superior, Middle, Inferior Temporal Gyrus, R Superior Temporal Sulcus, R Fusiform, R Posterior Cerebellum,	63	-34	-18	9.66	<0.001	6100
		58	-44	-12	9.22	<0.001	
		54	-36	2	7.80	<0.001	
		52	-3	-36	6.22	0.003	120
	R Angular Gyrus, R Supramarginal Gyrus, R Temporo-Parietal Junction, R Inferior Parietal Lobule, R Temporal Pole, R Superior, Inferior Temporal Gyrus, R Superior Temporal Sulcus, R Superior, Middle Occipital Gyrus, R Postcentral Gyrus, R Fusiform, R Lingual Gyrus, R Parahippocampal Gyrus	48	-60	32	9.53	<0.001	4133
		39	-68	39	9.12	<0.001	
		44	-51	46	8.03	<0.001	
		48	12	-39	5.56	0.035	9
		28	-46	-6	6.66	0.001	141
	LR Precuneus, LR Cuneus, LR Middle, Posterior Cingulate Cortex, LR Calcarine Sulcus	-6	-39	36	8.73	<0.001	4103
		8	-40	34	8.04	<0.001	
		-10	-52	15	7.51	<0.001	
		18	-48	4	5.61	0.030	8
	L Caudate	-8	8	9	6.80	<0.001	220
	R Hippocampus, R Parahippocampal Gyrus	27	-18	-18	5.88	<0.012	16
	L Hippocampus	-26	-20	-16	6.50	0.001	48
	LR Anterior, Posterior Cerebellum, Vermis	36	-62	-27	6.49	<0.001	179
		24	-75	-26	5.55	0.037	
		-30	-64	-27	6.45	0.001	236
		38	-63	-42	6.45	0.002	97
		3	-52	-52	6.36	0.002	327
		-6	-57	-51	6.27	0.003	
		0	-46	-16	6.24	0.003	101

Note. Main model comparing grey matter volume changes between the Pre and Post sessions in the PRG and CTR group corrected for the participants' medical history. Statistics are extracted from two-sample t-tests performed within the framework of an SPM12 General Linear Model and are one-sided (as is standard in SPM). Results are reported at a statistical threshold of $p<0.05$ FWE-corrected. P-value at peak voxel (whole-brain FWE corrected) is reported. PRG = nulliparous women who were pregnant between sessions, CTR = nulliparous women who were not pregnant between sessions, L = left, R = right.

Supplementary Table 5. Changes in grey matter volume (PRE to POST) corrected for fertility treatment or twins.

Contrasts	Regions	MNI coordinates *x*	*y*	*z*	*T*	*p* (FWE-corrected)	Cluster Size (mm³)
PRG > CTR	-						
CTR > PRG	R Superior, Middle, Inferior Temporal Gyrus, R Superior Temporal Sulcus	63	-35	-18	9.77	<0.001	4978
		60	-42	-14	9.07	<0.001	
		54	-17	-12	7.14	<0.001	
	L Superior, Middle, Inferior Temporal Gyrus, L Superior Temporal Sulcus, L Fusiform, L Angular Gyrus, L Temporo-Parietal Junction, L Temporal Pole, L Superior, Inferior Parietal Lobule, L Superior, Middle, Inferior Occipital Gyrus, L Lingual Gyrus, L Parahippocampal Gyrus	-52	-53	44	8.76	<0.001	10824
		-42	-69	38	7.92	<0.001	
		-65	-30	-15	7.09	<0.001	
		-32	-59	-8	6.48	0.001	298
		-60	17	-30	6.06	0.003	100
	LR Superior Medial Frontal Cortex, LR Superior, Middle, Inferior Frontal Gyrus, LR Orbitofrontal Cortex, LR Supplementary Motor Area, LR Precentral Gyrus, LR Anterior, Middle Cingulate Cortex, LR Gyrus Rectus, LR Olfactory Gyrus, LR Insula, L Temporal Pole	17	63	35	9.44	<0.001	39417
		-26	29	57	9.09	<0.001	
		17	50	44	8.50	<0.001	
		-51	11	20	7.48	<0.001	994
		-40	18	8	6.19	0.002	
		39	27	-12	6.12	0.002	209
		26	48	-17	5.77	0.009	55
		51	14	14	5.67	0.014	51
		50	20	9	5.43	0.036	
		-5	8	54	5.42	0.035	6
		-38	21	-15	7.36	<0.001	1337
		-38	36	-17	6.19	0.002	
		-17	12	-20	6.07	0.001	
		44	33	20	5.84	0.007	340
		45	39	26	5.77	0.009	
		34	41	-17	5.34	0.048	1
	R Superior, Middle, Inferior Temporal Gyrus, R Superior Temporal Sulcus, R Angular Gyrus, R Supramarginal Gyrus, R Temporo-Parietal Junction, R Superior, Inferior Parietal Lobule, R Superior, Middle Occipital Gyrus, R Temporal Pole	48	-62	32	8.55	<0.001	3054
		41	-66	39	7.92	<0.001	
		44	-53	48	7.51	<0.001	
		53	-2	-38	5.42	0.035	9
		48	11	-38	5.34	0.048	2
	LR Precuneus, LR Middle, Posterior Cingulate, LR Calcarine Sulcus, LR Cuneus, R Lingual Gyrus	-8	-41	35	8.05	<0.001	2190
		6	-30	34	7.08	<0.001	
		12	-54	21	6.35	0.001	
		-11	-54	15	6.69	<0.001	232
		18	-48	3	6.09	0.003	38
	L Hippocampus	-27	-17	-18	5.90	0.006	31
	R Hippocampus, R Parahippocampal Gyrus, R Fusiform	27	-20	-18	6.54	<0.001	79
		30	-29	-28	5.43	0.014	
	L Caudate	-9	6	12	6.34	0.001	146
	R Caudate	11	6	14	5.56	0.021	8
	R Anterior, Posterior Cerebellum	35	-62	-27	5.97	0.004	62
		38	-63	-42	5.84	0.007	37
		5	-54	-53	5.37	0.043	29
	L Anterior, Posterior Cerebellum	-35	-63	-29	6.16	0.00	133
		-8	-56	-50	5.50	0.026	16

	Vermis	0	-47	-17	5.69	0.013	30

Note. Main model comparing grey matter volume changes between the Pre and Post sessions in the PRG and CTR groups, excluding the women who underwent fertility treatment or delivered twins (leaving a PRG sample size of N = 36). Statistics are extracted from two-sample t-tests performed within the framework of an SPM12 General Linear Model and are one-sided (as is standard in SPM). Results are reported at a statistical threshold of p<0.05 FWE-corrected. P-value at peak voxel (whole-brain FWE corrected) is reported. PRG = nulliparous women who were pregnant between sessions, CTR = nulliparous women who were not pregnant between sessions, L = left, R = right.

Supplementary Table 6. Changes in grey matter volume (PRE to POST) excluding the participant who took part in the PRG group after completing the control trajectory.

Contrasts	Regions	MNI coordinates			*T*	*p* (FWE-corrected)	Cluster Size (mm^3)
		x	*y*	*z*			
PRG > CTR	-						
CTR > PRG	R Middle, Inferior, Superior Temporal Gyrus, R Temporal Pole, R Superior Temporal Sulcus	63	-36	-18	9.95	<0.001	5197
		62	-24	-16	9.43	<0.001	
		54	-16	-12	7.51	<0.001	
		52	-3	-36	6.09	0.004	62
		48	10	-38	5.52	0.035	6
	LR Superior Medial Frontal Cortex, LR Inferior, Middle, Superior Frontal Gyrus, LR Orbitofrontal Cortex, LR Supplementary Motor Area, LR Precentral Gyrus, LR Anterior, Middle Cingulate Cortex, LR Gyrus Rectus, L Olfactory Gyrus, LR Temporal Pole, LR Insula	-26	27	58	9.77	<0.001	49194
		20	50	42	9.63	<0.001	
		3	60	34	9.15	<0.001	
		39	27	-12	6.39	0.001	496
		33	42	-16	6.00	0.006	129
		24	48	-20	5.67	0.021	
		27	33	-16	5.53	0.034	6
	L Superior, Middle, Inferior Temporal Gyrus, L Superior Temporal Sulcus, L Angular Gyrus, L Temporo-Parietal Junction, L Superior, Middle, Inferior Occipital Gyrus, L Fusiform, L Supramarginal Gyrus, L Superior, Inferior Parietal Lobule, L Lingual Gyrus, L Parahippocampal Gyrus, L Postcentral Gyrus	-51	-54	42	9.72	<0.001	13535
		-42	-68	38	8.97	<0.001	
		-60	-46	-9	8.61	<0.001	
	R Angular Gyrus, R Temporo-Parietal Junction, R Superior, Inferior Parietal Lobule, R Superior Temporal Sulcus, R Supramarginal Gyrus, R Superior, Middle Occipital Gyrus, R Superior, Middle, Inferior Temporal Gyrus, R Postcentral Gyrus, R Fusiform	48	-60	33	9.03	<0.001	4371
		39	-68	39	9.03	<0.001	
		40	-62	45	8.72	<0.001	
		40	-26	-27	5.88	0.010	155
		44	-33	-21	5.69	0.019	
	LR Precuneus, LR Middle, Posterior Cingulate Cortex, LR Calcarine Sulcus, LR Cuneus	-8	-39	34	8.22	<0.001	3405
		-12	-54	15	7.82	<0.001	
		6	-38	36	7.30	<0.001	
		18	-48	3	5.85	0.011	28
	L Caudate	-8	8	9	6.83	<0.001	193
	R Hippocampus, R Parahippocampal Gyrus	27	-20	-18	6.34	0.002	44
	L Hippocampus	-27	-18	-18	6.18	0.003	41
	LR Anterior, Posterior Cerebellum, Vermis	36	-60	-28	6.39	0.001	108
		-33	-65	-27	6.27	0.002	146
		2	-46	-16	6.22	0.003	97
		3	-54	-52	6.12	0.004	236
		-6	-56	-50	5.96	0.007	
		38	-63	-42	6.09	0.005	71

Note. Main model comparing grey matter volume changes between the Pre and Post sessions in the PRG and CTR groups, excluding the woman who participated in the PRG group after completing the study trajectory in the CTR group (leaving a PRG sample size of N = 39). Statistics are extracted from two-sample t-tests performed within the framework of an SPM12 General Linear Model and are one-sided (as is standard in SPM). Results are reported at a statistical threshold of p<0.05 FWE-corrected. P-value at peak voxel (whole-brain FWE corrected) is reported. PRG = nulliparous women who were pregnant between sessions, CTR = nulliparous women who were not pregnant between sessions, L = left, R = right.

Supplementary Table 7. Total brain volumes Pre and Post sessions.

		PRG (Mean ± SD)	CTR (Mean ± SD)	Between-Group Differences
GM (L)	PRE	0.731 ± 0.060	0.741 ± 0.066	*t*= -1.180, *p* = 0.242
	POST	0.714 ± 0.059	0.735 ± 0.067	*t*= -1.452, *p* = 0.151
WM (L)	PRE	0.388 ± 0.037	0.391 ± 0.033	*t*= -0.355, *p* = 0.723
	POST	0.389 ± 0.037	0.392 ± 0.036	*t*= -0.427, *p* = 0.671
TBV (L)	PRE	1.119 ± 0.089	1.131 ± 0.093	*t*= -0.609, *p* = 0.544
	POST	1.103 ± 0.089	1.127 ± 0.097	*t*= -1.156, *p* = 0.251

Note. Total grey matter, white matter and total brain volume of the two groups at the Pre and Post sessions. Between-group differences were analyzed using two-sided two-sample t-tests. Repeated Measures General Linear Models comparing the change in total tissue volumes across sessions between the groups were also performed, which revealed significant group*session interaction effects for the changes in grey matter (F=24.39, p<0.001) and total brain volume (F=22.67, p<0.001) but not for white matter (F=0.221, p=0.640). Pre = pre-pregnancy session; Post = post-pregnancy session; L = liter; GM = grey matter; WM = white matter; TBV = total brain volume.

Supplementary Table 8. Total brain volumes late postpartum period (Post+1y).

	Late Postpartum (Mean ± SD)
GM (L)	0.712 ± 0.056
WM (L)	0.384 ± 0.038
TBV (L)	1.096 ± 0.087

Note. Values of total grey matter, white matter and total brain volume for the late postpartum sessions in the women who became pregnant during this study are provided in this table. L = liter; GM = grey matter; WM = white matter; TBV = total brain volume.

Supplementary Table 9. Changes in grey matter volume (PRE to POST) corrected for TBV change.

Contrasts	Regions	MNI coordinates			T	p (FWE-corrected)	Cluster Size (mm³)
		x	*y*	*z*			
PRG > CTR	-						
CTR > PRG	L Angular Gyrus, L Supramarginal Gyrus, L Temporo-Parietal Junction, L Inferior Parietal Lobule, L Middle Occipital Gyrus, L Middle, Inferior Temporal Gyrus, L Superior Temporal Sulcus, L Middle, Posterior Cingulate Cortex, L Precuneus	-51	-52	44	8.28	<0.001	1760
		-42	-68	38	7.30	<0.001	
		-48	-56	24	6.51	0.001	
		-66	-30	-15	7.11	<0.001	1241
		-60	-46	-9	6.87	<0.001	
		-63	-40	-18	6.75	<0.001	
		-8	-40	36	6.93	<0.001	109
		-10	-54	15	5.79	0.014	14
	R Inferior, Middle Temporal Gyrus, R Superior Temporal Sulcus, R Angular Gyrus, R Supramarginal Gyrus, R Temporo-Parietal Junction, R Superior, Inferior Parietal Lobule, R Middle, Superior Occipital Gyrus, R Middle Cingulate Cortex	63	-34	-18	8.25	<0.001	1361
		62	-24	-16	7.74	<0.001	
		60	-42	-14	7.50	<0.001	
		48	-60	33	8.04	<0.001	952
		40	-66	39	7.36	<0.001	
		44	-51	46	6.64	0.001	
		8	-39	36	5.76	0.016	27
		54	-36	2	5.53	0.035	2
	LR Superior, Middle, Inferior Frontal Gyrus, LR Superior Medial Frontal Cortex, L Precentral Gyrus, L Supplementary Motor Area, LR Gyrus Rectus, LR Orbitofrontal Cortex, L Temporal Pole	-26	27	58	7.83	<0.001	7452
		18	50	42	7.75	<0.001	
		12	60	33	7.45	<0.001	
		-50	9	20	6.47	0.001	73
		2	40	-24	6.24	0.003	199
		-8	34	-18	5.64	0.024	
		-4	27	-16	5.53	0.035	
		2	63	-10	5.59	0.003	161
		-38	21	-15	6.16	0.004	*63*
		-32	60	4	6.06	0.005	83
		-30	54	24	5.97	0.007	197
		6	72	-4	5.60	0.028	9
		16	68	15	5.53	0.036	6
		-46	18	8	5.51	0.038	4
	L Caudate	-6	6	9	5.76	0.016	18

Note. Main model comparing grey matter volume changes between the Pre and Post sessions in the PRG and CTR groups corrected for changes in total brain volume. Statistics are extracted from two-sample t-tests performed within the framework of an SPM12 General Linear Model and are one-sided (as is standard in SPM). Results are reported at a statistical threshold of p<0.05 FWE-corrected. P-value at peak voxel (whole-brain FWE corrected) is reported. PRG = nulliparous women who were pregnant between sessions, CTR = nulliparous women who were not pregnant between sessions, L = left, R = right.

Supplementary Table 10. Quantification of overlap between GM volume changes of pregnancy (PRE to POST) and the brain's cognitive networks

Map1	Volume map (mm^3)	Observed overlap (mm^3)	Observed overlap (%Map1)	Observed overlap (%δPRG)	Expected overlap (mm^3)	Observed / expected overlap
Comp 1	229105	11084	3.62	4.84	71294	0.16
Comp 2	221549	12342	4.03	5.57	68943	0.18
Comp 3	183971	37736	12.31	20.51	57249	0.66
Comp 4	235852	14070	4.59	5.97	73394	0.19
Comp 5	194876	48614	15.86	24.95	60643	0.80
Comp 6	194214	25252	8.24	13.00	60437	0.42
Comp 7	206442	14708	4.80	7.12	64242	0.23
Comp 8	218987	57996	18.92	26.48	68146	0.85
Comp 9	217411	73683	24.03	33.89	67655	1.09
Comp 10	180326	65526	21.37	36.34	56115	1.17
Comp 11	194241	36288	11.84	18.68	60445	0.60
Comp 12	184910	30736	10.03	16.62	57541	0.53

Note. The overlap of the changes in GM volume across pregnancy were quantified with the cognitive networks of the large-scale meta-analysis of cerebral functional network organization by Yeo et al. [1]. The tasks recruited by the cognitive components of Yeo et al. are depicted in an interactive map (https://surfer.nmr.mgh.harvard.edu/fswiki/BrainmapOntology_Yeo2015). The overlap of our results with these functional networks was extracted by computing the intersection between each of these maps and the GM volume changes of pregnancy (column 'Observed overlap'). The percentages of each of the maps represented by the overlap were subsequently determined and reported in percentage of the functional map (column 'Observed overlap (%Map1))' and in percentage of the map of GM volume changes of pregnancy (column 'Observed overlap (%δPRG))'. The expected overlap based on a random distribution across the brain was determined by multiplying the percentages of the brain's total GM represented by each of the 2 maps. The percentage of expected overlap was then multiplied by total GM (column 'Expected overlap'), and the expected overlap was divided by the observed overlap (column 'Observed>Expected overlap'). Comp= component, δPRG = changes in GM volume across pregnancy.

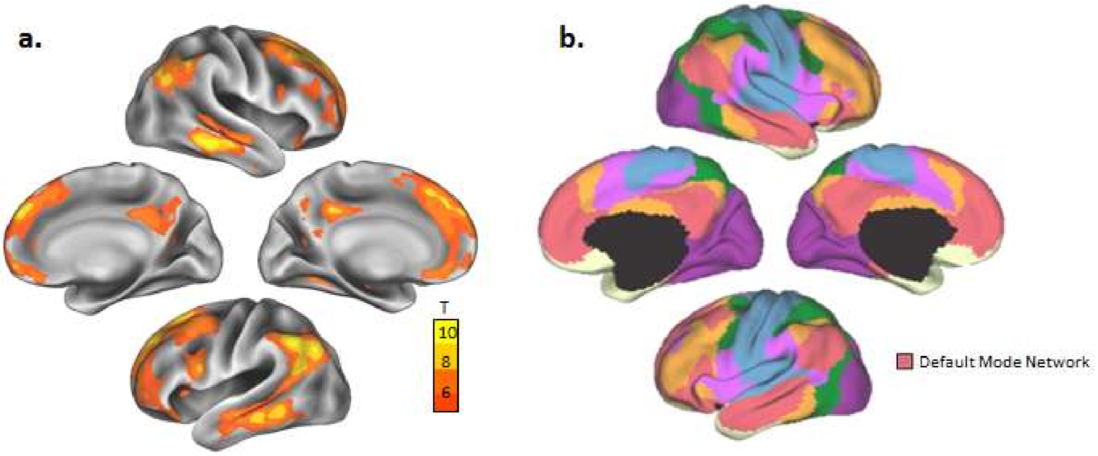

Supplementary Figure 2. Spatial overlap quantification analyses. a) Surface maps displaying the observed changes in grey matter volume in women. **b)** The networks of intrinsic connectivity as defined by Yeo et al.[2] in 'The organization of the human cerebral cortex estimated by intrinsic functional connectivity' published in 2011 in the Journal of Neurophysiology (J. Neurophysiology 106, 1125). The red color represents the Default Mode Network. Other colors represent the visual network (purple), the somatosensory network (blue), the dorsal attention network (green), the ventral attention network (violet), the limbic network (cream) and the frontoparietal network (orange).

Supplementary Table 11: Quantification of overlap between GM volume changes of pregnancy (PRE to POST) and networks of intrinsic functional connectivity by Yeo et al.

Map1	Volume map (mm^3)	Observed overlap (mm^3)	Observed overlap (%Map1)	Observed overlap (%δPRG)	Expected overlap (mm^3)	Observed / expected overlap
Comp 1	178136	3368	1.10	1.89	55433	0.06
Comp 2	155939	1286	0.42	0.82	48526	0.03
Comp 3	123073	12396	4.04	10.07	38298	0.32
Comp 4	109637	11570	3.77	10.55	34117	0.34
Comp 5	92316	9713	3.17	10.52	28728	0.34
Comp 6	155509	75799	24.72	48.74	48392	1.57
Comp 7	241012	133009	43.39	55.19	74999	1.77

Note. The overlap of the changes in GM volume across pregnancy were quantified with the networks of intrinsic functional connectivity defined by Yeo et al.[2]. The overlap of our results with these functional networks was extracted by computing the intersection between each of these maps and the GM volume changes of pregnancy (column 'Observed overlap'). The percentages of each of the maps represented by the overlap were subsequently determined and reported in percentage of the functional map (column 'Observed overlap (%Map1))' and in percentage of the map of GM volume changes of pregnancy (column 'Observed overlap (%δPRG))'. The expected overlap based on a random distribution across the brain was determined by multiplying the percentages of the brain's total GM represented by each of the 2 maps. The percentage of expected overlap was then multiplied by total GM (column 'Expected overlap'), and the expected overlap was divided by the observed overlap (column 'Observed>Expected overlap'). Comp= component, Comp 1 = Visual Network, Comp 2 = Somatosensory Network, Comp 3 = Dorsal Attention Network, Comp 4 = Ventral Attention Network, Comp 5 = Limbic Network, Comp 6 = Frontoparietal Network, Comp 7 = Default Mode Network, δPRG = changes in GM volume across pregnancy.

Supplementary Table 12: Quantification of overlap between GM volume changes of pregnancy (PRE to POST) and networks of intrinsic functional connectivity by Smith et al.

Map1	Volume map (mm^3)	Observed overlap (mm^3)	Observed overlap (%Map1)	Observed overlap (%δPRG)	Expected overlap (mm^3)	Observed / expected overlap
Comp 1	1027127	121622	11.84	9.12	319627	0.38
Comp 2	1014319	134963	13.31	10.22	315641	0.43
Comp 3	1089929	138929	12.75	9.95	339170	0.41
Comp 4	1048353	175004	16.69	12.92	326232	0.54
Comp 5	990070	129131	13.04	9.96	308095	0.42
Comp 6	1093109	160043	14.64	11.43	340159	0.47
Comp 7	1079939	143535	13.29	10.35	336061	0.43
Comp 8	1148361	179921	15.67	12.37	357353	0.50
Comp 9	1139896	186276	16.34	12.88	354719	0.53
Comp 10	1098363	190279	17.32	13.54	341795	0.56

Note. The overlap of the changes in GM volume across pregnancy were quantified with the networks of intrinsic functional connectivity defined by Smith et al.[3]. The overlap of our results with these functional networks was extracted by computing the intersection between each of these maps and the GM volume changes of pregnancy (column 'Observed overlap'). The percentages of each of the maps represented by the overlap were subsequently determined and reported in percentage of the functional map (column 'Observed overlap (%Map1))' and in percentage of the map of GM volume changes of pregnancy (column 'Observed overlap (%δPRG))'. The expected overlap based on a random distribution across the brain was determined by multiplying the percentages of the brain's total GM represented by each of the 2 maps. The percentage of expected overlap was then multiplied by total GM (column 'Expected overlap'), and the expected overlap was divided by the observed overlap (column 'Observed>Expected overlap'). Comp= component, Comp 1 = Visual Network 1, Comp 2 = Visual Network 2, Comp 3 = Visual Network 3, Comp 4 = Default Mode Network, Comp 5 = Cerebellar Network, Comp 6 = Sensorimotor Network, Comp 7 = Auditory Network, Comp 8 = Executive Control Network, Comp 9 = Fronto-parietal Network 1, Comp 10 = Fronto-parietal Network 2, δPRG = changes in GM volume across pregnancy.

Supplementary Table 13. Changes in default mode network connectivity (PRE to POST).

Contrasts	Regions	MNI coordinates			*T*	*P* (FWE-corrected)	Cluster Size (mm^3)
		x	*y*	*z*			
(PRG post>pre) > (CTR post>pre)	L cuneus, R cuneus	6	-84	18	4.69	0.019	135
(PRG pre>post) > (CTR pre>post)	-						

Note. Results of interaction contrasts between group (PRG vs CTR) and session (PRE and POST) for the default mode network component. The other networks rendered no significant results. PRG = women who were pregnant between sessions, CTR = women who were not pregnant between sessions. Results are reported at a statistical threshold of P<0.05 FWE-corrected (P value at peak voxel).

Supplementary Table 14. Changes in within-network connectivity (PRE to POST).

Contrasts	Direction	Regions	MNI coordinates			*T*	*P* (FWE-corrected)	Cluster Size (mm³)
			x	*y*	*z*			
Visual 3	increase	R lingual	18	-51	-6	4.81	0.013	108
	decrease	-						
DMN	increase	L cuneus, R cuneus	6	-84	18	5.55	0.001*	297
	decrease	-						

Note. Changes across pregnancy in within-network connectivity in each of the networks. The other neural networks rendered no significant results. Results are extracted from one-sided t-tests performed in SPM12 and reported at a threshold of P<0.05 FWE-corrected (P value peak voxel). * also present in group*session interaction effect.

Supplementary Table 15. Baseline differences in DMN connectivity (PRE).

Contrasts	Regions	MNI coordinates			*T*	*P* (FWE-corrected)	Cluster Size (mm³)
		x	*y*	*z*			
PRG > CTR	L Precuneus	-3	-75	39	5.30	0.002	675
	L Angular, L Inferior Parietal	-36	-72	45	4.55	0.029	52
CTR > PRG	-						

Note. Results of one-sided two-sample t-tests performed within the framework of the SPM12 General Linear Model testing for baseline differences in DMN connectivity. A region of interest (ROI) analysis involving the region of change observed in the main analysis indicated that there is no overlap between the observed cluster and the region undergoing changes across pregnancy.

Supplementary Table 16. Correlation between baseline (PRE) differences and DMN coherence changes across pregnancy.

Measures	R	p
Within PRG: Region 1 (-3 -75 39)	-.012	.940
Within PRG: Region 2 (-36 -72 45)	-.060	.711
In whole sample: Region 1 (-3 -75 39)	.040	.730
In whole sample: Region 2 (-36 -72 45)	.068	.557

Note. Correlation results between the baseline signal values extracted from the clusters in the Supplementary Table above and the observed changes in default mode network coherence across pregnancy.

Supplementary Table 17. Changes in DMN coherence (PRE to POST), excluding the women with the delayed time interval.

Contrasts	Regions	MNI coordinates			*T*	*P* (FWE-corrected)	Cluster Size (mm³)
		x	*y*	*z*			
(PRG post>pre) > (CTR post>pre)	L cuneus, R cuneus	6	-84	18	4.85	0.012	162
(PRG pre>post) > (CTR pre>post)	-						

Note. Results of interaction contrasts between group (PRG vs CTR) and session (Pre and Post) for the DMN component within the framework of an SPM12 General Linear Model, excluding the women who initially did not participate in the Post session and therefore had a delayed time interval (>800 days) between the sessions (leaving a PRG sample size of N = 37). PRG= women who were pregnant between sessions, CTR = women who were not pregnant between sessions. Results are reported at a statistical threshold of p<0.05 FWE-corrected (P value at peak voxel).

Supplementary Table 18. Changes in DMN coherence (PRE to POST), excluding the participant who took part in the PRG group after completing the control trajectory.

Contrasts	Regions	MNI coordinates			*T*	*P* (FWE-corrected)	Cluster Size (mm³)
		x	*y*	*z*			
(PRG post>pre) > (CTR post>pre)	L cuneus, R cuneus	6	-84	18	4.77	0.015	162
(PRG pre>post) > (CTR pre>post)	-						

Note. Results of interaction contrasts between group (PRG vs CTR) and session (Pre and Post) for the DMN component within the framework of an SPM12 General Linear Model, excluding the participant who participated first as a control subject and subsequently as a PRG participant (leaving a PRG sample size of N = 39). PRG= women who were pregnant between sessions, CTR = women who were not pregnant between sessions. Results are reported at a statistical threshold of p<0.05 FWE-corrected (P value at peak voxel).

Supplementary Table 19. Changes in DMN coherence (PRE to POST), excluding the participant who had twins.

Contrasts	Regions	MNI coordinates			*T*	*P* (FWE-corrected)	Cluster Size (mm³)
		x	*y*	*z*			
(PRG post>pre) > (CTR post>pre)	L cuneus, R cuneus	6	-84	18	4.61	0.024	162
(PRG pre>post) > (CTR pre>post)	-						

Note. Results of interaction contrasts between group (PRG vs CTR) and session (Pre and Post) for the DMN component within the framework of an SPM12 General Linear Model, excluding the participant who had twins (leaving a PRG sample size of N = 39). PRG= women who were pregnant between sessions, CTR = women who were not pregnant between sessions. Results are reported at a statistical threshold of p<0.05 FWE-corrected (P value at peak voxel).

Supplementary Table 20. Changes in DMN coherence (PRE to POST), correcting for the participants' medical history.

Contrasts	Regions	MNI coordinates			*T*	*P* (FWE-corrected)	Cluster Size (mm³)
		x	*y*	*z*			
(PRG post>pre) > (CTR post>pre)	L cuneus, R cuneus	6	-81	15	4.32	0.064	
(PRG pre>post) > (CTR pre>post)	-						

Note. Results of interaction contrasts between group (PRG vs CTR) and session (Pre and Post) for the DMN component within the framework of an SPM12 General Linear Model, correcting for the participants' medical

history. PRG= women who were pregnant between sessions, CTR = women who were not pregnant between sessions. Results are reported at a statistical threshold of p<0.05 FWE-corrected (P value at peak voxel).

Supplementary Table 21. Changes in DMN coherence (PRE to POST), excluding the participants who had undergone fertility treatment.

**Contrasts**	**Regions**	**MNI coordinates**			***T***	***P* (FWE-corrected)**	**Cluster Size (mm³)**
		x	***y***	***z***			
(PRG post>pre) > (CTR post>pre)	L cuneus, R cuneus	6	-84	18	4.43	0.046	27
(PRG pre>post) > (CTR pre>post)	-						

Note. Results of interaction contrasts between group (PRG vs CTR) and session (Pre and Post) for the DMN component within the framework of an SPM12 General Linear Model, excluding the participants who had undergone fertility treatment (leaving a PRG sample size of N = 37). PRG= women who were pregnant between sessions, CTR = women who were not pregnant between sessions. Results are reported at a statistical threshold of p<0.05 FWE-corrected (P value at peak voxel).

Supplementary Table 22. Changes in DMN correlations across pregnancy (PRE to POST).

	**DMN**	
	F	*p*
Visual network 2	.417	.520
Visual network 3	1.325	.218
Sensorimotor network	.111	.740
Visual network 1	.242	.624
Perception-pain network	.149	.700
Auditory network	.137	.712
Cognition/language network	.496	.484
Cerebellum network	.000	.994
Executive control network	.011	.919

Note. F-statistics and p-values of DMN correlations session (Pre vs Post) * subject group (primiparous vs control). Interaction effects were extracted from Repeated-Measures General Linear Models.

Supplementary Table 23. Changes in network correlations (PRE to POST) between all resting-state networks across pregnancy.

	Visual 2	Visual 3	Sensori motor	Visual 1	DMN	Perception-pain	Aud-itory	Cognitionl anguage	Cere-bellum	Executive control
**Visual 2**	-	.865	.880	.031	.520	.637	.317	.724	.445	.006
**Visual 3**	.865	-	.854	.971	.218	.364	.315	.509	.562	.759
**Sensori-motor**	.880	.854	-	.709	.740	.752	.016	.650	.220	.369
**Visual 1**	.031	.971	.709	-	.624	.624	.276	.493	.839	.163
**DMN**	.520	.218	.740	.624	-	.700	.712	.484	.994	.919
**Perception-pain**	.637	.364	.752	.624	.700	-	.576	.669	.531	.123
**Auditory**	.317	.315	.016	.276	.712	576	-	.176	.731	.077
**Cognition-language**	.724	.509	.650	.493	.484	.669	.176	-	.738	.981
**Cere-bellum**	.445	.562	.220	.839	.994	.531	.731	.738	-	.874
**Executive control**	.006	.759	.369	.163	.919	.123	.077	.981	.874	-

Note. GLM p-values of two-sided interaction effect session (Pre vs Post) * group (primiparous vs control) of between-network correlations of all networks. *P<0.05 corrected for multiple comparisons. None of these effects survive a correction for multiple comparisons.

Supplementary Table 24. Baseline comparisons (PRE) neural metabolite concentrations.

**Metabolite**	**T (U)**	**P (p U)**
tNAA	1.144	.256
Cho	.142	.887
tCr	.659 (695)	.512 (.783)
Glu	1.646	.104
Ins	.117	.907

Note. Comparisons between the metabolite concentrations in the Pre session between women who were pregnant between scans (PRG) in comparison to women who were not (CTR) in the Posterior Cingulate Cortex. In case of any deviations from normality, non-parametric Mann- Whitney U tests were performed. The U values and p-values of these tests are reported between brackets in the table. None of these effects survive a correction for multiple comparisons. tNAA = N-acetylaspartate (including contributions from N-acetylaspartylglutamate), Cho =

Choline (phosphorylcholine and glycerophosphorylcholine), tCr = Creatine (creatine and phosphocreatine), Glu = Glutamate , Ins = myo-Inositol.

Supplementary Table 25. Changes in neural metabolite concentrations (PRE to POST).

Metabolite	Session	Metabolite concentrations PRG (Mean±SD)	CTR (Mean±SD)	F (U)	P (p U)
tNAA	PRE	11.18 ± .41	11.23 ± .44	.091	.764
	POST	11.38 ± .56	11.39 ± .44		
Cho	PRE	1.21 ± .12	1.20 ± .15	4.316	.041
	POST	1.38 ± .13	1.29 ± .17		
tCr	PRE	7.50 ± .51	7.51 ± .46	4.136	.046
	POST	8.04 ± .41	7.78 ± .46	(536)	(.054)
Glu	PRE	9.67 ± .68	9.91 ± .71	.031	.861
	POST	9.99 ± .64	10.19 ± .78		
Ins	PRE	4.38 ± .48	4.33 ± .52	2.156	.146
	POST	4.75 ± .54	4.52 ± .50	(530)	(.047)

Note. Changes in metabolite concentrations between Pre and Post sessions in women who were pregnant between scans (PRG) in comparison to women who were not (CTR) in the Posterior Cingulate Cortex. In case of any deviations from normality in one of the groups or sessions, non-parametric Mann- Whitney U tests were performed on the Post-Pre difference values. The U values (“U”) and p-values (“p U”) of these tests are reported between brackets in the table. None of these effects survive a correction for multiple comparisons. tNAA = N-acetylaspartate (including contributions from N-acetylaspartylglutamate), Cho = Choline (phosphorylcholine and glycerophosphorylcholine), tCr = Creatine (creatine and phosphocreatine), Glu = Glutamate , Ins = myo-Inositol.

Supplementary Table 26. Effect sizes for the changes (PRE to POST) in spectroscopic results.

Metabolite	η_p^2
tNAA	.001
Cho	.055
tCr	.053
Glu	.000
Ins	.028

Note. Partial eta squared values for the changes in metabolite concentrations between Pre and Post sessions in women who were pregnant between scans (PRG) in comparison to women who were not (CTR) in the Posterior Cingulate Cortex Volume of Interest. tNAA = N-acetylaspartate (including contributions from N-acetylaspartylglutamate), Cho = Choline (phosphorylcholine and glycerophosphorylcholine), tCr = Creatine (creatine and phosphocreatine), Glu = Glutamate , Ins = myo-Inositol.

Supplementary Table 27. Changes in neural metabolite concentrations (PRE to POST) excluding the participants with the delayed time interval.

Metabolite	F (U)	P (p U)
tNAA	.062	.804
Cho	3.880	.053
tCr	3.877 (495)	.053 (.059)
Glu	.041	.841
Ins	1.477 (497)	.228 (.062)

Note. Changes in metabolite concentrations between Pre- and Post-pregnancy sessions in women who were pregnant between scans (PRG) in comparison to women who were not (CTR), excluding the women who initially did not participate in the Post session and therefore had a delayed time interval (>800 days) between the sessions (leaving a PRG sample size of N = 36). Session (pre-post pregnancy) * group (PRG, CTR) interaction effects are reported. In case of any deviations from normality in one of the groups or sessions, non-parametric Mann-Whitney U tests were performed on the Post-Pre difference values. The U values ("U") and p-values ("p U") of these tests are reported between brackets in the table. None of these effects survive a correction for multiple comparisons. tNAA = N-acetylaspartate (including contributions from N-acetylaspartylglutamate), Cho = Choline (phosphorylcholine and glycerophosphorylcholine), tCr = Creatine (creatine and phosphocreatine), Glu = Glutamate , Ins = myo-Inositol.

Supplementary Table 28. Changes in metabolite concentrations (PRE to POST) excluding the participant who took part in the PRG group after completing the control trajectory.

Metabolite	F (U)	P (p U)
tNAA	.154	.696
Cho	4.223	.043
tCr	4.075 (520)	.047 (.052)
Glu	.024	.877
Ins	2.195 (513)	.143 (.044)

Note. Changes in metabolite concentrations between Pre and Post sessions in women who were pregnant between scans (PRG) in comparison to women who were not (CTR), excluding the woman who had participated in this study both as a nulliparous control and subsequently also as a pregnant women (leaving a PRG sample size of N = 38). Session (pre-post pregnancy) * group (PRG, CTR) interaction effects are reported. In case of any deviations from normality in one of the groups or sessions, non-parametric Mann- Whitney U tests were performed on the Post-Pre difference values. The U values ("U") and p-values ("p U") of these tests are reported between brackets in the table. None of these effects survive a correction for multiple comparisons. tNAA = N-acetylaspartate (including contributions from N-acetylaspartylglutamate), Cho = Choline (phosphorylcholine and glycerophosphorylcholine), tCr = Creatine (creatine and phosphocreatine), Glu = Glutamate , Ins = myo-Inositol.

Supplementary Table 29. Changes in metabolite concentrations (PRE to POST) excluding the women who underwent fertility treatment or who had twins.

Metabolite	F (U)	P (p U)
tNAA	.005	.944
Cho	3.940	.051
tCr	3.575 (528)	.063 (.064)
Glu	.040	.842
Ins	4.323 (526)	.041 (.061)

Note. Changes in metabolite concentrations between Pre- and Post-pregnancy sessions in women who were pregnant between scans (PRG) in comparison to women who were not (CTR), excluding the women who underwent fertility treatment or who had twins (leaving a PRG sample size of N = 35). Session (pre-post pregnancy) * group (PRG, CTR) interaction effects are reported. In case of any deviations from normality in one of the groups or sessions, non-parametric Mann- Whitney U tests were performed on the Post-Pre difference values. The U values ("U") and p-values ("p U") of these tests are reported between brackets in the table. None of these effects survive a correction for multiple comparisons. tNAA = N-acetylaspartate (including contributions from N-acetylaspartylglutamate), Cho = Choline (phosphorylcholine and glycerophosphorylcholine), tCr = Creatine (creatine and phosphocreatine), Glu = Glutamate , Ins = myo-Inositol.

Supplementary Table 30. Changes in metabolite concentrations (PRE to POST) corrected for a previous history of medical or psychiatric disorders.

Metabolite	F	p
tNAA	.710	.403
Cho	4.135	.046
tCr	3.669	.060
Glu	.361	.550
Ins	1.014	.318

Note. Changes in metabolite concentrations between Pre- and Post-pregnancy sessions in women who were pregnant between scans (PRG) in comparison to women who were not (CTR), while correcting for a previous history of medical or psychiatric disorders. Session (pre-post pregnancy) * group (PRG, CTR) interaction effects are reported. None of these effects survive a correction for multiple comparisons. tNAA = N-acetylaspartate (including contributions from N-acetylaspartylglutamate), Cho = Choline (phosphorylcholine and glycerophosphorylcholine), tCr = Creatine (creatine and phosphocreatine), Glu = Glutamate, Ins = myo-Inositol.

Supplementary Table 31. Correlations between observed changes across pregnancy.

Changes	R (rho)	P (p rho)
GM & rsfMRI	-.23	.784
GM & tCr	.18	.121
GM & Cho	.14	.177
GM & Ins	.56	.001
rsfMRI & tCr	.092	.579
rsfMRI & Cho	.031	.849
rsfMRI & Ins	.200 (.186)	.221 (.258)

Note. Correlation results between the observed changes across pregnancy. In case of deviations from normality, a non-parametric Spearman's correlation test was performed rather than a Pearson's test. Spearman's rho ("rho") and the p-values for this test ("p rho") are then also reported in the table. The correlations with grey matter were performed using multivariate regression analyses using Kernel Ridge Regression. GM=observed changes in grey matter volume, rsfMRI=observed changes in temporal coherence of the Default Mode Network, tCr=observed changes in creatine concentrations, Cho=observed changes in choline concentrations, Ins=observed changes in myo-inositol.

Supplementary Table 32. Changes in grey matter volume in the late postpartum period relative to the pre-pregnancy session (PRE to POST+1y).

Contrasts	Regions	MNI coordinates			*T*	*p* (FWE-corrected)	Cluster Size (mm^3)
		x	*y*	*z*			
Increase	-						
Decrease	LR Superior Medial Frontal Cortex, LR Superior, Middle, Inferior Frontal Gyrus, LR Orbitofrontal Cortex, LR Supplementary Motor Area, LR Precentral Gyrus, LR Anterior, Middle Cingulate Cortex, LR Gyrus Rectus, LR Insula, LR Olfactory Gyrus, LR Temporal Pole, LR Caudate, LR Putamen, L Fusiform, R Pallidum	45	10	36	16.38	<0.001	85673
		-24	22	51	15.89	<0.001	
		-46	20	22	14.40	<0.001	
		38	-8	20	7.07	<0.001	108
		22	-3	70	6.57	<0.001	4
	LR Precuneus, LR Middle, Posterior Cingulate Cortex, LR Cuneus, LR Calcarine Sulcus, LR Cuneus	9	-60	34	11.23	<0.001	5954
		10	-54	24	10.28	<0.001	
		12	-44	38	9.87	<0.001	
	L Superior, Inferior Parietal Lobule, L Superior, Middle, Inferior Temporal Gyrus, L Superior Temporal Sulcus, L Angular Gyrus, L Supramarginal Gyrus, L Temporo-Parietal Junction, L Superior, Middle, Inferior Occipital Gyrus, L Thalamus, L Parahippocampal Gyrus, L Fusiform, L Lingual Gyrus	-48	-54	24	11.20	<0.001	3546
		-27	-70	40	9.06	<0.001	
		-6	-26	6	7.59	<0.001	44
		-32	-38	-14	7.36	<0.001	58
		-34	-78	-4	7.35	<0.001	85
		-32	-54	-4	6.63	<0.001	11
		-26	-82	4	6.61	<0.001	8
		-36	-75	10	6.56	<0.001	4
		-18	-78	-3	6.49	<0.001	1
	R Angular Gyrus, R Supramarginal Gyrus, R Temporo-Parietal Junction, R Superior, Middle Occipital Gyrus, R Superior, Inferior Parietal Lobule, R Lingual Gyrus, R Fusiform, R Thalamus	50	-58	33	10.83	<0.001	3258
		39	-50	48	8.23	<0.001	
		38	-60	40	8.15	<0.001	
		30	-52	-3	7.51	<0.001	41
		33	-87	3	6.95	<0.001	19
		10	-21	9	6.84	<0.001	5
		32	-33	-18	6.80	<0.001	11
		14	-74	-6	6.53	<0.001	2
	LR Anterior, Posterior Cerebellum	-20	-72	-26	9.71	<0.001	2115
		-28	-78	-27	8.78	<0.001	
		-36	-58	-44	7.84	<0.001	
		36	-60	-26	8.91	<0.001	826
		32	-62	-39	8.71	<0.001	
		20	-70	-26	8.54	<0.001	
		-3	-60	-51	7.48	<0.001	125
		-12	-58	-48	7.02	<0.001	
		6	-58	-50	6.92	<0.001	
		9	-58	-16	7.19	<0.001	20
	L Caudate, L Putamen	-10	12	8	9.46	<0.001	528
		-21	3	0	6.83	<0.001	

Note. Grey matter volume changes between the late postpartum session and the pre-conception baseline in the PRG participants. Statistics are extracted from one-sample t-tests performed within the framework of an SPM12 General Linear Model and are one-sided (as is standard in SPM). P-value at peak voxel (whole-brain FWE corrected) is reported extracted from one-sample t-tests perform. L = left, R = right.

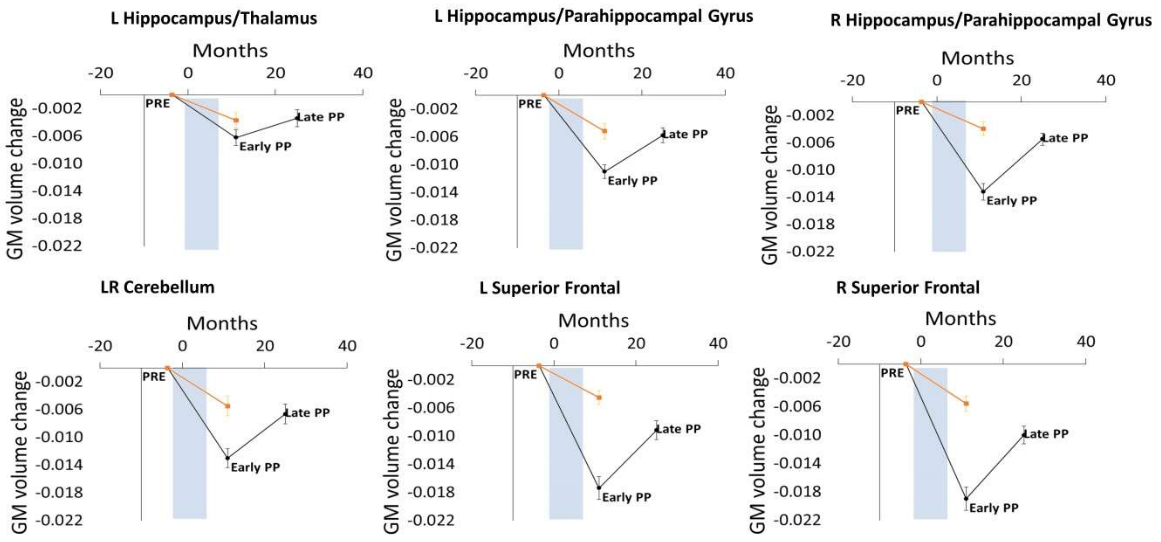

Supplementary Figure 3. Plots of volume changes from pre-conception to late postpartum of the PRG and CTR sample. Mean (± SEM) grey matter volume changes at each Post session (Post and Post+1y) relative to the Pre-pregnancy baseline of the most significant clusters (i.e., T > 8), extracted from the smoothed normalized Jacobian difference images for each cluster. Red line represents control group (no Post+1y data is available of the control group). Blue bar represents pregnancy. GM = grey matter, L = left, R = right, PP = Postpartum.

Supplementary Table 33. Changes in grey matter volume in primiparous mothers across the postpartum period (POST to POST+1y).

Contrasts	Regions	MNI coordinates			*T*	*p* (FWE-corrected)	Cluster Size (mm³)
		x	*y*	*z*			
Increase	R Hippocampus, R Parahippocampal Gyrus, R Precuneus, R Posterior Cingulate Cortex, R Lingual Gyrus, R Calcarine Sulcus, R Thalamus, R Fusiform, R Inferior Temporal Gyrus, R Cerebellum	28	-18	-15	10.49	<0.001	780
		24	-32	-14	7.28	<0.001	
		32	-38	-24	7.01	<0.001	
		15	-44	8	7.66	<0.001	254
		24	-32	8	7.14	<0.001	
		40	-21	-26	7.18	<0.001	29
		16	-64	-14	7.10	<0.001	48
	L Hippocampus	-32	-40	-6	9.43	<0.001	720
		-27	-16	-10	7.49	<0.001	
		-10	-34	9	8.26	<0.001	105
	R Superior, Middle, Inferior Frontal Gyrus, R Superior Medial Frontal Cortex, R Orbitofrontal Cortex, R Insula	26	57	6	8.46	<0.001	847
		36	54	-2	7.30	<0.001	
		14	68	14	7.19	<0.001	
		50	-10	12	7.85	<0.001	210
		22	18	48	7.46	<0.001	339
		16	50	24	6.74	<0.001	71
		50	10	8	6.67	<0.001	18
		32	45	20	6.65	<0.001	35
		44	-76	32	6.55	<0.001	10
		46	22	-3	6.44	<0.001	6
	L Superior, Middle, Inferior Frontal Gyrus, L Superior Medial Frontal Cortex, L Orbitofrontal Cortex	-21	66	6	8.05	<0.001	1176
		-15	64	15	7.56	<0.001	
		-32	56	4	7.36	<0.001	
		-2	56	38	7.92	<0.001	564
		0	45	46	6.88	<0.001	
		-9	34	50	6.76	<0.001	55
		-27	8	52	6.76	<0.001	79
		-24	30	36	6.65	<0.001	41
		-45	39	3	6.52	<0.001	15
	L Superior, Middle Temporal Gyrus, L Superior Temporal Sulcus, L Angular Gyrus, L Supramarginal Gyrus, L Temporo-Parietal Junction, L Middle Occipital Gyrus	-48	-60	22	7.54	<0.001	295
		-62	-54	22	6.69	<0.001	
		-44	-69	36	6.99	<0.001	83
		-33	-80	24	6.45	<0.001	1
	LR Posterior Cerebellum	2	-50	-45	8.27	<0.001	605
		0	-54	-54	7.85	<0.001	
		-4	-62	-48	7.39	<0.001	
		16	-40	-48	6.80	<0.001	18
		12	-39	-21	6.54	<0.001	7
		12	-45	-42	6.43	<0.001	4
	LR Precuneus, L Cuneus	-2	-68	27	7.32	<0.001	150
	LR Superior Medial Frontal Cortex	3	28	42	6.67	<0.001	28
Decrease	-						

Note. Changes in grey matter volume across the postpartum period in primiparous mothers. Statistics are extracted from one-sample t-tests performed within the framework of an SPM12 General Linear Model and are one-sided (as is standard in SPM). P-value at peak voxel (whole-brain FWE corrected) is reported. L = left, R = right.

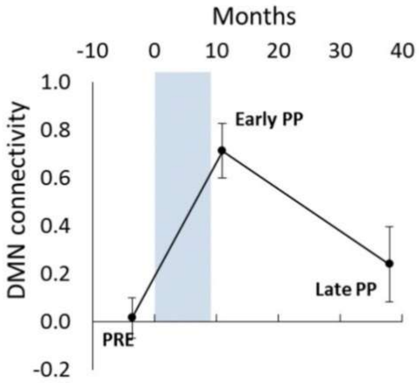

Supplementary Figure 4. Changes in DMN coherence from pre-conception until the late postpartum period. Mean (± SEM) levels of DMN connectivity at each Post session (Post and Post+1y) relative to the Pre-pregnancy baseline (N = 28) in women who became pregnant during this study. Blue bar represents pregnancy. GM = grey matter, L = left, R = right, PP = Postpartum.

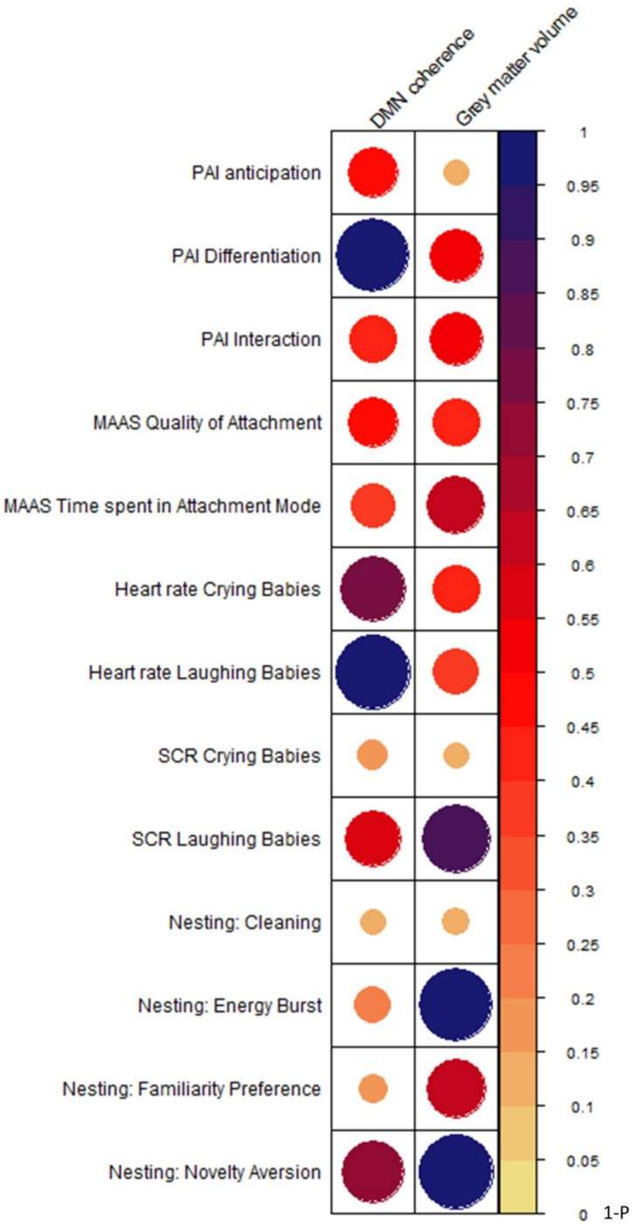

Supplementary Figure 5. Correlation results prenatal measures. Plot depicting correlations between the neural changes across pregnancy and all prenatal measures. DMN coherence = changes in Default Mode Network coherence between Pre and Post sessions. Grey matter volume = changes in grey matter volume between Pre and Post sessions, 1-P = reverse p-values, PAI = Prenatal Attachment Inventory, MAAS = Maternal Antenatal Attachment Scale, Nesting = Nesting Questionnaire, SCR = Skin Conductance Response.

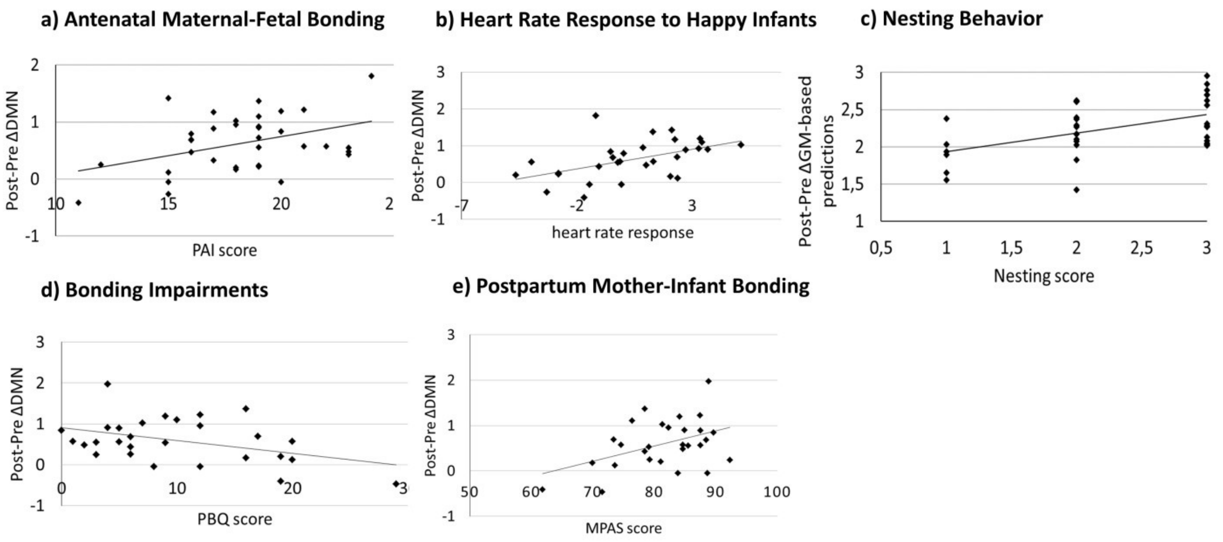

Supplementary Figure 6. Functional correlates of neural changes across pregnancy (PRE to POST). Plots depicting correlations between the neural changes across pregnancy and a) the Differentiation scale of the Prenatal Attachment Inventory (PAI), b) interval between heart rate peaks (peak RR) in response to movies of laughing babies, c) the Social Selectivity Scale of the Nesting Behavior Questionnaire, d) the late postpartum Postpartum Bonding Questionnaire (PBQ, total score), e) the late postpartum Maternal Postnatal Attachment Scale (MPAS, total score). It should be noted that for the multivariate regression analyses, the depicted values represent predicted values of the correlate based on the pattern of brain changes across the whole brain and the depicted values thus do not represent the direction of the biological effect. Post-Pre ΔDMN = Difference in DMN coherence between Pre and Post session, Post-PreΔGM-based predictions = Predicted covariate values based on changes in grey matter between Pre and Post sessions defined by multivariate regression analyses.

Supplementary Table 34. Correlations between Nesting questionnaire and PRE to POST changes in DMN coherence.

Measure	R (rho)	P (p rho)
Space Preparation: Cleaning	.068<br>(.026)	.697<br>(.884)
Space Preparation: Energy Burst	-.079<br>(-.054)	.654<br>(.759)
Social Selectivity: Familiarity Preference	.041<br>(.035)	.817<br>(.843)
Social Selectivity: Novelty Aversion	-.229<br>(-.186)	.186<br>(.284)

Note. Correlation results between the Nesting Questionnaire and the observed changes in the coherence of the default mode network. In case of deviations from normality, a non-parametric Spearman's correlation test was performed rather than a Pearson's test. Spearman's rho ("rho") and the p-values for this test ("p rho") are then also reported in the table. *P<0.05 corrected for multiple comparisons (correlation-adjusted Bonferroni correction for the number of tests within the research question). **Effect also survives correction for multiple comparisons for all prenatal tests (correlation-adjusted Bonferroni correction for the number of performed tests with prenatal measures for this modality).

Supplementary Table 35. Correlations between Nesting questionnaire and PRE to POST changes in grey matter volume.

Measure	R	p
Space Preparation: Cleaning	-.34	.866
Space Preparation: Energy Burst	.33	.040
Social Selectivity: Familiarity Preference	-.00	.372
Social Selectivity: Novelty Aversion	.50	.003**

Note. Correlation results between the Nesting Questionnaire and the observed changes in grey matter volume based on multivariate regression analyses. It should be noted that for these analyses the R cannot be interpreted as a direct reflection of the direction of the biological effect, since this statistic is based on patterns of brain changes across the whole brain. *P<0.05 corrected for multiple comparisons (correlation-adjusted Bonferroni correction for the number of tests within the research question). **effect also survives correction for multiple comparisons for all prenatal tests (correlation-adjusted Bonferroni correction for the number of performed tests with prenatal measures for this modality).

Supplementary Table 36. Correlations between PAI, MAAS and PRE to POST changes in DMN coherence.

Measure	R (rho)	P (p rho)
PAI Anticipation	.110	.529
PAI Differentiation	.399	.018*
PAI Interaction	.094	.590
MAAS Quality of Attachment	.189<br>(.110)	.277<br>(.530)
MAAS Time spent in Attachment Mode	.082	.639

Note. Correlation results between the Prenatal Attachment Inventory (PAI) and Maternal Antenatal Attachment Scale (MAAS) and the observed changes in default mode network coherence. In case of deviations from normality, a non-parametric Spearman's correlation test was performed rather than a Pearson's test. Spearman's rho ("rho") and the p-values for this test ("p rho") are then also reported in the table. *P<0.05 corrected for multiple comparisons (correlation-adjusted Bonferroni correction for the number of tests within the research question). **effect also survives correction for multiple comparisons for all prenatal tests (correlation-adjusted Bonferroni correction for the number of performed tests with prenatal measures for this modality).

Supplementary Table 37. Correlations between PAI, MAAS and PRE to POST changes in grey matter volume.

Measure	R	p
PAI Anticipation	-.34	.881
PAI Differentiation	-.04	.488
PAI Interaction	-.04	.470
MAAS Quality of Attachment	-.12	.584
MAAS Time spent in Attachment Mode	.01	.373

Note. Correlation results between the Prenatal Attachment Inventory (PAI) and Maternal Antenatal Attachment Scale (MAAS) and the changes in grey matter volume based on multivariate regression analyses. It should be noted that for these analyses the R cannot be interpreted as a direct reflection of the direction of the biological effect, since this statistic is based on patterns of brain changes across the whole brain. *P<0.05 corrected for multiple comparisons (correlation-adjusted Bonferroni correction for the number of tests within the research question).

**effect also survives correction for multiple comparisons for all prenatal tests (correlation-adjusted Bonferroni correction for the number of performed tests with prenatal measures for this modality).

Supplementary Table 38. Correlations between physiological responses and PRE to POST changes in DMN coherence.

Measure	R (rho)	P (rho p)
Heart rate Crying Babies	.224	.235
Heart rate Laughing Babies	.507	.004**
SCR Crying Babies	-.075 (-.042)	.694 (.824)
SCR Laughing Babies	.117 (.158)	.552 (.421)

Note. Correlation results between the physiological responses (interval between heart rates and skin conductance response (SCR)) to movies of laughing and crying babies and the observed changes in default mode network coherence. In case of deviations from normality, a non-parametric Spearman's correlation test was performed rather than a Pearson's test. Spearman's rho ("rho") and the p-values for this test ("p rho") are then also reported in the table. *P<0.05 corrected for multiple comparisons (correlation-adjusted Bonferroni correction for the number of tests within the research question). **effect also survives correction for multiple comparisons for all prenatal tests (correlation-adjusted Bonferroni correction for the number of performed tests with prenatal measures for this modality).

Supplementary Table 39. Correlations between physiological responses and PRE to POST changes in grey matter volume.

Measure	R	p
Heart rate Crying Babies	-.14	.587
Heart rate Laughing Babies	-.15	.615
SCR Crying Babies	-.36	.878
SCR Laughing Babies	.18	.143

Note. Correlation results between the physiological responses (interval between heart rates and skin conductance response (SCR)) to movies of laughing and crying babies and the changes in grey matter volume based on multivariate regression analyses. It should be noted that for these analyses the R cannot be interpreted as a direct reflection of the direction of the biological effect, since this statistic is based on patterns of brain changes across the whole brain. *P<0.05 corrected for multiple comparisons (correlation-adjusted Bonferroni correction for the number of tests within the research question). **effect also survives correction for multiple comparisons for all prenatal tests (correlation-adjusted Bonferroni correction for the number of performed tests with prenatal measures for this modality).

Supplementary Note 1

Postpartum Mother-Infant Bonding and Bonding Impairments

In addition to the association with gestational changes in a mother that prepare for motherhood, we wanted to investigate whether the observed brain changes across pregnancy could predict a mother's bonding to her infant after birth and problems in the mother-infant relationship. Mother-infant bonding was measured using the Maternal Postnatal Attachment Scale (MPAS) in the early (Post) and late (Post+1y) postpartum session. In addition, impairments in the mother-infant relationship were assessed with the Postpartum Bonding Questionnaire (PBQ) in the early (Post) and late (Post+1y) postpartum session. First, correlation analyses were performed with the total MPAS and PBQ measures acquired in the early and late postpartum period to examine the time point when potential associations could be observed. These analyses revealed no associations with the early postpartum measures, while changes in default mode activity across pregnancy significantly predicted both mother-infant bonding and bonding impairments in the late postpartum period (Supplementary Figure 7, Supplementary Tables 40 – 43), with stronger brain changes being associated with more mother-infant bonding and less impairments in the mother-infant relationship. Subsequent analyses of the subscores of these scales in the late postpartum period did not render significant results, although a trend was observed with impaired bonding and the risk of infant rejection (Supplementary Tables 44 – 45).

Development of Mother-Infant Bonding and Bonding Problems across the Postpartum Period

Based on the observed associations with these measures in the late but not the early postpartum period, we hypothesized that neurally-regulated effects on bonding may actually only become evident in the postpartum period. Therefore, we performed supplementary analyses to test whether pregnancy-related changes in DMN coherence relate to subsequent developments in the mother-infant relationship that take place in the postpartum period (Supplementary Figure 8 and 9). These analyses showed that stronger pregnancy-related increases in DMN coherence predicted a stronger increase in bonding and a decrease in bonding impairments across the postpartum period (Supplementary Table 46). Associations were additionally observed with changes in the degree of pleasure experienced by the mother in the interaction with her baby and the absence of hostility (Supplementary Tables 47). Furthermore, pregnancy-related changes in DMN coherence were associated with postpartum changes in the risk of infant rejection and pathological anger across the postpartum period (Supplementary Tables 48). It should be noted that these changes were not associated with the neural changes across the postpartum period, only with the preceding changes across pregnancy. When applying a Bonferroni correction across all postnatal measures, the correlation between the changes in DMN coherence and the postpartum changes in mother-infant bonding and infant rejection and pathological anger remained significant. These findings thus reveal associations between pregnancy-related neural changes and mother-infant bonding and bonding impairments across the postpartum period, suggesting that neurogestational effects on aspects of maternal caregiving may affect the subsequent bonding of a mother to her infant across the postpartum period.

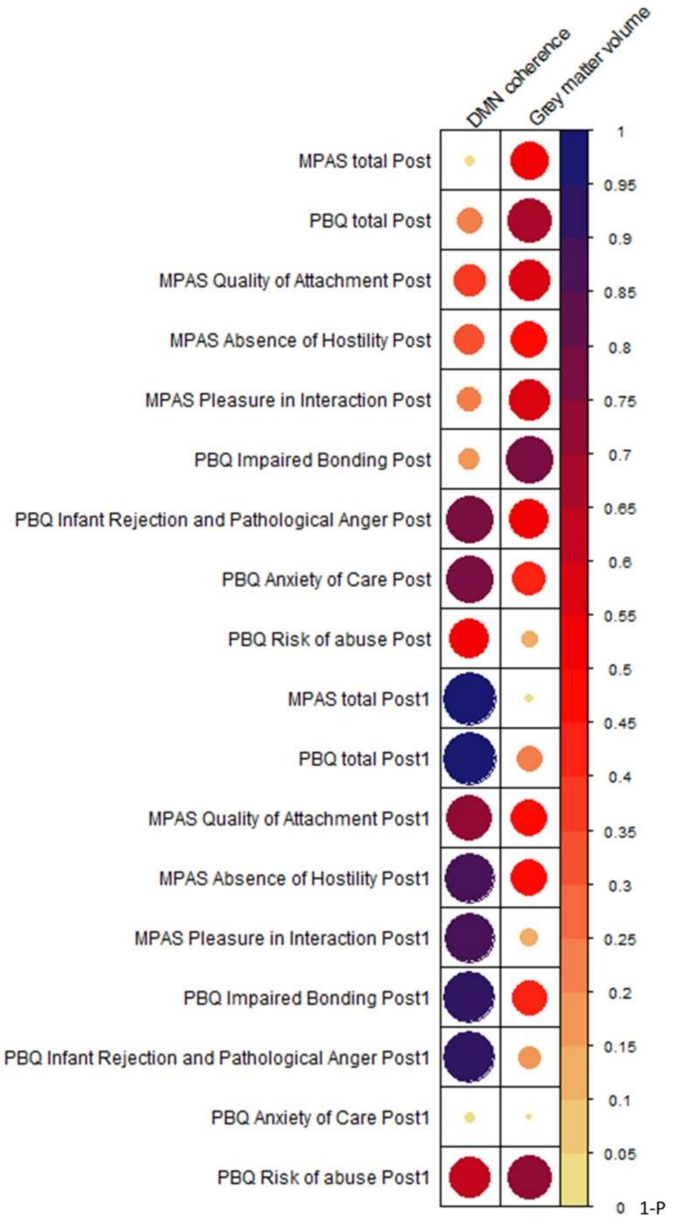

Supplementary Figure 7. Correlation results postnatal measures. Plot depicting correlations between the neural changes across pregnancy and all postnatal measures (except for the Post+1y – Post measures, depicted in the next figure). Note that the Post analyses for both modalities and the Post+1y analyses for grey matter volume have only been added for illustrative purposes but were not performed as part of the main analyses (since only the Post+1y totals of the MPAS were significant). DMN coherence = changes in Default Mode Netwerk coherence between Pre and Post sessions. Grey matter volume = changes in grey matter volume between Pre and Post sessions, 1-P = reverse p-values, Post1 = Post+1y session, MPAS = Maternal Postnatal Attachment Scale, PBQ = Postpartum Bonding Questionnaire.

Supplementary Table 40. Correlations between PRE to POST changes in DMN coherence and early (POST) and late postpartum (POST+1y) MPAS.

Measure	R (rho)	P (p rho)
MPAS early postpartum	.004<br>(.008)	.983<br>(.962)
MPAS late postpartum	.440	.019**

Note. Correlation results between the total Maternal Postnatal Attachment Scale (MPAS) scores in the early and late postpartum period with the observed changes in the coherence of the default mode network. In case of deviations from normality, a non-parametric Spearman's correlation test was performed rather than a Pearson's test. Spearman's rho ("rho") and the p-values for this test ("p rho") are then also reported in the table. *P<0.05 corrected for multiple comparisons (correlation-adjusted Bonferroni correction for the number of tests within the research question). **effect also survives correction for multiple comparisons for all postpartum tests (correlation-adjusted Bonferroni correction for the number of performed tests with postpartum measures for this modality).

Supplementary Table 41. Correlations between PRE to POST changes in DMN coherence and early (POST) and late postpartum (POST+1y) PBQ.

Measure	R (rho)	P (p rho)
PBQ early postpartum	.075<br>(-.048)	.648<br>(.772)
PBQ late postpartum	-.413	.029**

Note. Correlation results between the total Postpartum Bonding Questionnaire (PBQ) scores in the early and late postpartum period with the observed changes in the coherence of the default mode network. In case of deviations from normality, a non-parametric Spearman's correlation test was performed rather than a Pearson's test. Spearman's rho ("rho") and the p-values for this test ("p rho") are then also reported in the table. *P<0.05 corrected for multiple comparisons (correlation-adjusted Bonferroni correction for the number of tests within the research question). **effect also survives correction for multiple comparisons for all postpartum tests (correlation-adjusted Bonferroni correction for the number of performed tests with postpartum measures for this modality).

Supplementary Table 42. Correlations between PRE to POST changes in grey matter volume and early (POST) and late postpartum (POST+1y) MPAS.

Measure	R	p
MPAS early postpartum	-.05	.482
MPAS late postpartum	-.56	.972

Note. Correlation results between the Maternal Postnatal Attachment Scale (MPAS) scores in the early and late postpartum period with the observed changes in grey matter volume based on multivariate regression analyses. It should be noted that for these analyses the R cannot be interpreted as a direct reflection of the direction of the biological effect, since this statistic is based on patterns of brain changes across the whole brain. *P<0.05 corrected for multiple comparisons (correlation-adjusted Bonferroni correction for the number of tests within the research question). **effect also survives correction for multiple comparisons for all postpartum tests (correlation-adjusted Bonferroni correction for the number of performed tests with postpartum measures for this modality).

Supplementary Table 43. Correlations between PRE to POST changes in grey matter volume and early (POST) and late postpartum (POST+1y) PBQ.

Measure	R	p
PBQ early postpartum	.05	.321
PBQ late postpartum	-.28	.753

Note. Correlation results between the Postpartum Bonding Questionnaire (PBQ) scores in the early and late postpartum period with the observed changes in grey matter volume based on multivariate regression analyses. It should be noted that for these analyses the R cannot be interpreted as a direct reflection of the direction of the biological effect, since this statistic is based on patterns of brain changes across the whole brain. *P<0.05 corrected for multiple comparisons (correlation-adjusted Bonferroni correction for the number of tests within the research question). **effect also survives correction for multiple comparisons for all postpartum tests (correlation-adjusted Bonferroni correction for the number of performed tests with postpartum measures for this modality).

Supplementary Table 44. Correlations between PRE to POST changes in DMN coherence and late postpartum (Post+1y) MPAS.

Measure	R (rho)	P (p rho)
Quality of Attachment	.311 (.224)	.107 (.251)
Absence of Hostility	.289	.136
Pleasure in Interaction	.434 (.296)	.021 (.126)

Note. Correlation results between the Maternal Postnatal Attachment Scale (MPAS) scores in the late postpartum period (Post+1) with the observed changes in the coherence of the default mode network. In case of deviations from normality, a non-parametric Spearman's correlation test was performed rather than a Pearson's test. Spearman's rho ("rho") and the p-values for this test ("p rho") are then also reported in the table. *P<0.05 corrected for multiple comparisons (correlation-adjusted Bonferroni correction for the number of tests within the research question). **effect also survives correction for multiple comparisons for all postpartum tests (correlation-adjusted Bonferroni correction for the number of performed tests with postpartum measures for this modality).

Supplementary Table 45. Correlations between PRE to POST changes in DMN coherence and late postpartum (Post+1y) PBQ.

Measure	R (rho)	P (p rho)
Impaired Bonding	-.333	.083
Infant Rejection and Pathological Anger	-.479 (-.358)	.010 (.061)
Anxiety of Care	-.152 (.011)	.441 (.955)
Risk of Abuse	-.319 (-.166)	.098 (.397)

Note. Correlation results between the Postpartum Bonding Questionnaire (PBQ) scores in the late postpartum period (Post+1year) with the observed changes in the coherence of the default mode network. In case of deviations from normality, a non-parametric Spearman's correlation test was performed rather than a Pearson's test. Spearman's rho ("rho") and the p-values for this test ("p rho") are then also reported in the table. *P<0.05 corrected for multiple comparisons.

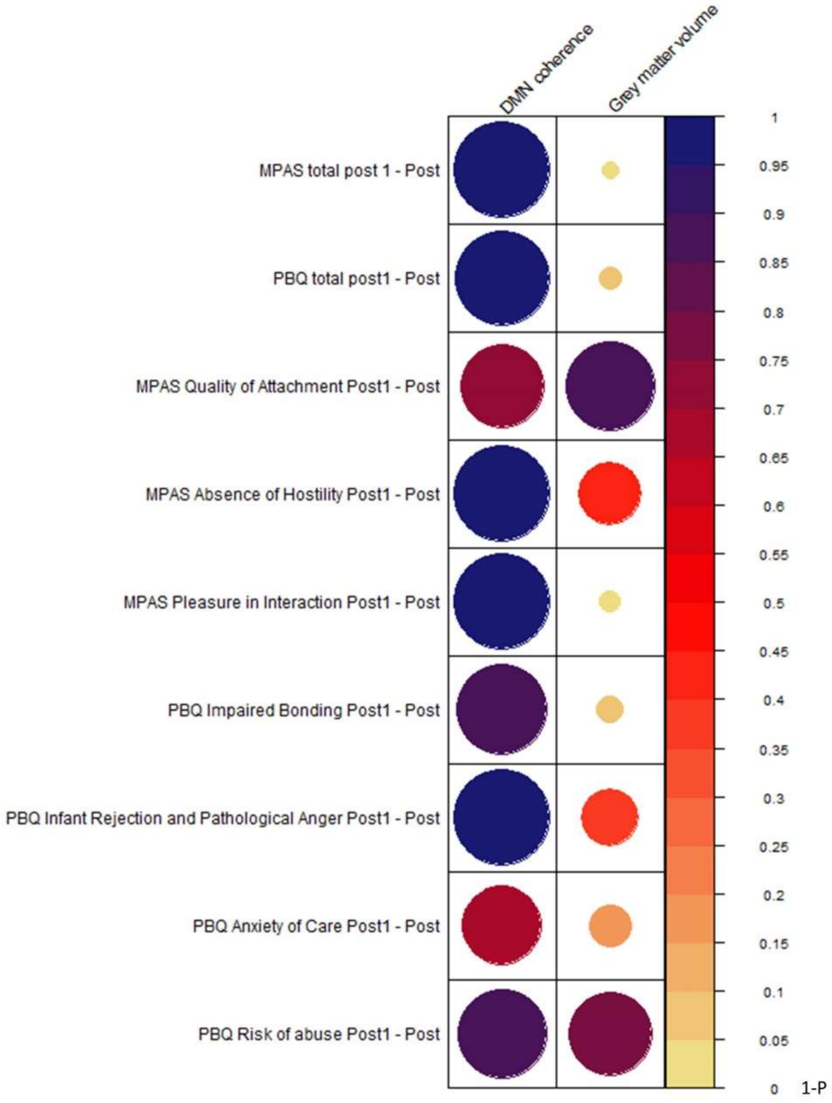

Supplementary Figure 8. Correlation results postnatal measures. Plot depicting correlations between the neural changes across pregnancy and the Post+1y – Post measures. Note that these analyses for grey matter volume have only been added for illustrative purposes but were not performed as part of the main analyses (since only the Post+1y - Post totals of the MPAS were significant). DMN coherence = changes in Default Mode Netwerk coherence between Pre and Post sessions. Grey matter volume = changes in grey matter volume between Pre and Post sessions, 1-P = reverse p-values, Post1 – Post = Post+1y - Post session, MPAS = Maternal Postnatal Attachment Scale, PBQ = Postpartum Bonding Questionnaire.

Supplementary Table 46. Correlations between PRE to POST changes in DMN coherence changes across pregnancy MPAS and PBQ changes across postpartum period (POST to POST+1y).

Measure	R	p
MPAS total	.628	<.0001**
PBQ total	-.430	.025*

Note. Correlation results between the changes in total Maternal Postnatal Attachment Scale (MPAS) and Postpartum Bonding Questionnaire (PBQ) scores across the postpartum period with the observed changes across pregnancy in the coherence of the default mode network. *P<0.05 corrected for multiple comparisons (correlation-adjusted Bonferroni correction for the number of tests within the research question). **effect also survives correction for multiple comparisons for all postpartum tests (correlation-adjusted Bonferroni correction for the number of performed tests with postpartum measures for this modality).

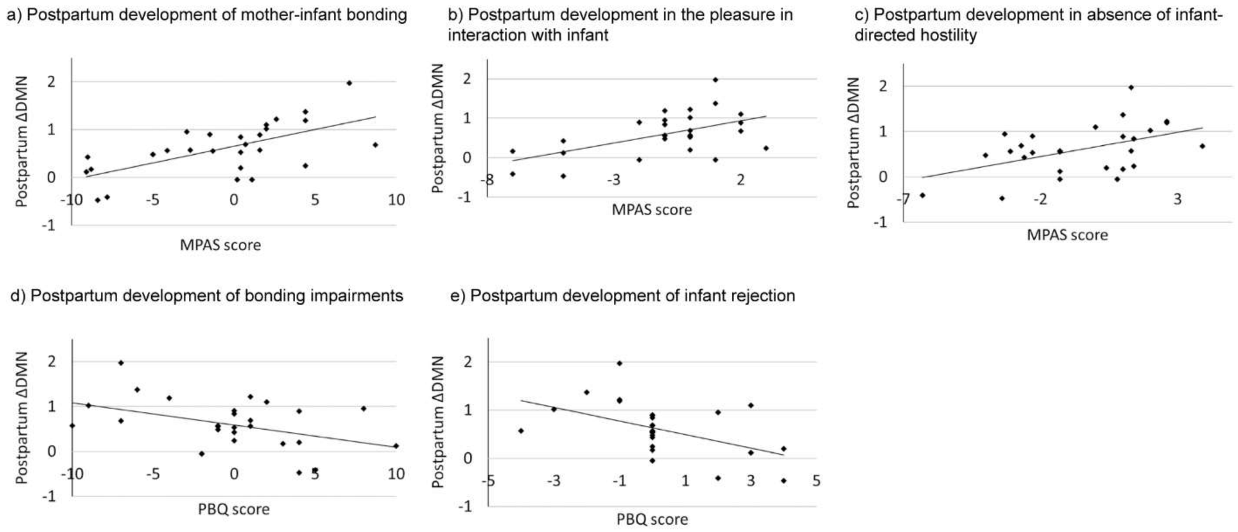

Supplementary Figure 9. Correlations between PRE to POST neural changes with postpartum changes in mother-infant bonding. Plots depicting correlations between neural changes across pregnancy and the postpartum development of a) mother-infant bonding extracted from the Maternal Postnatal Attachment Scale (MPAS, total score), b) the Pleasure in Interaction extracted from the MPAS, c) the Absence of Hostility extracted from the MPAS, d) problems in the mother-infant relationship extracted from the Postpartum Bonding Questionnaire (PBQ, total score), e) Infant Rejection extracted from the PBQ. Post-Pre ΔDMN = Difference in DMN coherence between Pre and Post session.

Supplementary Table 47. Correlations between DMN coherence change across pregnancy (PRE to POST) and MPAS changes across postpartum period (POST to POST+1y).

Measure	R (rho)	P (p rho)
Quality of Attachment	.228	.253
Absence of Hostility	.477	.012*
Pleasure in Interaction	.545 (.463)	.003 (.015*)

Note. Correlation results between the changes in Maternal Postnatal Attachment Scale (MPAS) scores across the postpartum period with the observed changes across pregnancy in the coherence of the default mode network. In

case of deviations from normality, a non-parametric Spearman's correlation test was performed rather than a Pearson's test. Spearman's rho ("rho") and the p-values for this test ("p rho") are then also reported in the table. *P<0.05 corrected for multiple comparisons (correlation-adjusted Bonferroni correction for the number of tests within the research question). **effect also survives correction for multiple comparisons for all postpartum tests (correlation-adjusted Bonferroni correction for the number of performed tests with postpartum measures for this modality).

Supplementary Table 48. Correlations between DMN coherence change across pregnancy (PRE to POST) and PBQ changes across postpartum period (POST to POST+1y)

Measure	R (rho)	P (p rho)
Impaired Bonding	-.303	.125
Infant Rejection and Pathological Anger	-.473 (-.564)	.013 (.002**)
Anxiety of Care	-.190 (-.197)	.344 (.323)
Risk of Abuse	-.342 (-.289)	.081 (.143)

Note. Correlation results between the changes in Postpartum Bonding Questionnaire (PBQ) scores across the postpartum period with the observed changes across pregnancy in the coherence of the default mode network. In case of deviations from normality, a non-parametric Spearman's correlation test was performed rather than a Pearson's test. Spearman's rho ("rho") and the p-values for this test ("p rho") are then also reported in the table. *P<0.05 corrected for multiple comparisons (correlation-adjusted Bonferroni correction for the number of tests within the research question). **effect also survives correction for multiple comparisons for all postpartum tests (correlation-adjusted Bonferroni correction for the number of performed tests with postpartum measures for this modality).

Supplementary Table 49. Correlations between hormones and changes in grey matter volume across pregnancy (PRE to POST).

Measure	R	p
Estradiol	.36	.017*
Estriol	.38	.022
Progesterone	-.23	.774
Cortisol	.02	.353

Note. Correlation results between hormone levels across pregnancy and the changes in grey matter volume based on multivariate regression analyses. It should be noted that for these analyses the R cannot be interpreted as a reflection of the direction of the biological effect, since this statistic is based on patterns of brain changes across the whole brain. *P<0.05 corrected for multiple comparisons (correlation-adjusted Bonferroni correction for the number of hormones).

Supplementary Table 50. Correlations between hormones and DMN coherence across pregnancy (PRE to POST).

Measure	R (rho)	p (p rho)
Estradiol	.037 (-.007)	.827 (.968)
Estriol	.077 (.012)	.651 (.942)
Progesterone	.015	.928
Cortisol	.099	.561

Note. Correlation results between hormone levels across pregnancy and the observed changes in DMN coherence. In case of deviations from normality, a non-parametric Spearman's correlation test was performed rather than a Pearson's test. Spearman's rho ("rho") and the p-values for this test ("p rho") are then also reported in the table.
*P<0.05 corrected for multiple comparisons (correlation-adjusted Bonferroni correction for the number of hormones).

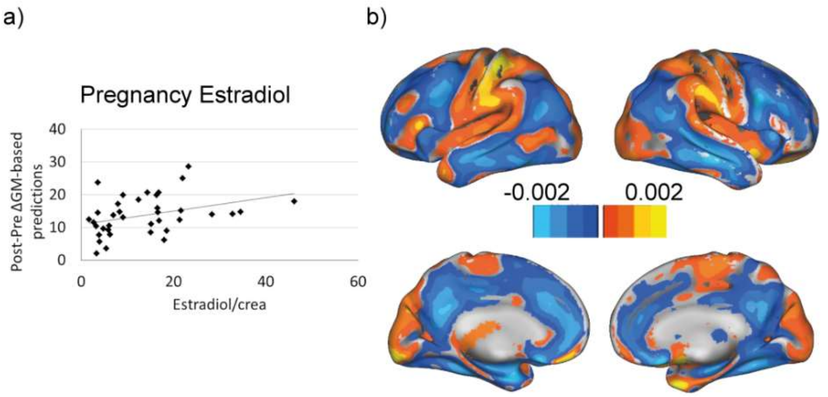

Supplementary Figure 10. Correlation between mean pregnancy Estradiol levels and the changes in brain structure across pregnancy (PRE to POST). a) Scatter plot depicting results from the multivariate regression analyses examining the relation between mean Estradiol levels (pg/ml) divided by creatinine (mg/dl) across pregnancy and changes in Grey Matter volume across pregnancy. It should be noted that for these analyses the direction of the depicted correlation cannot be interpreted as a reflection of the direction of the biological effect,

since this statistic is based on patterns of brain changes across the whole brain. b) Weight map depicting the relative contribution of each voxel to the multivariate regression. Note that blue colors depict a negative contribution to the regression, reflecting that higher Estradiol levels in pregnancy are associated with stronger volume reductions within the blue regions. Post-PreΔGM-based predictions = Predicted covariate values based on changes in grey matter between Pre and Post sessions defined by multivariate regression analyses.

Supplementary Table 51. Correlations between estradiol levels and grey matter volume across pregnancy (PRE to POST).

Pregnancy week	R	p
8	.06	.260
12	.19	.039
16	-.01	.375
20	.07	.250
24	.22	.095
28	.25	.041
32	.41	.016
36	.22	.076
38	.27	.080

Note. Correlation results between Estradiol levels across pregnancy and the changes in grey matter volume based on multivariate regression analyses. Note that correlations were not performed with any hormone levels extracted from week 40 due to the low number of available samples. It should be noted that for these analyses the R cannot be interpreted as a reflection of the direction of the biological effect, since this statistic is based on patterns of brain changes across the whole brain.

Supplementary Table 52. Correlations between osmolality levels and PRE to POST changes in grey matter volume.

Osmolality levels	R	p
Trimester 1	-.38	.891
Trimester 2	-.10	.570
Trimester 3	.17	.133
Whole pregnancy	-.15	.628

Note. Correlation results between mean osmolality levels averaged across whole pregnancy and for each of the trimesters of pregnancy with the changes in grey matter volume based on multivariate regression analyses. It should be noted that for these analyses the R cannot be interpreted as a reflection of the direction of the biological effect, since this statistic is based on patterns of brain changes across the whole brain. *P<0.05 corrected for multiple comparisons.

Supplementary Table 53. Correlations between osmolality levels and PRE to POST changes in DMN coherence.

Osmolality levels	R	p
Trimester 1	.048	.791
Trimester 2	.065	.704
Trimester 3	-.097	.570
Whole pregnancy	.041	.808

Note. Correlation results between mean osmolality levels averaged across whole pregnancy and for each of the trimesters of pregnancy with the changes in default mode network coherence.*P<0.05 corrected for multiple comparisons.

Supplementary Table 54. Correlations between osmolality levels and PRE to POST changes in choline across pregnancy.

Osmolality levels	R	p
Trimester 1	.257	.156
Trimester 2	-.087	.612
Trimester 3	.390	.019
Whole pregnancy	.310	.079

Note. Correlation results between mean osmolality levels averaged across whole pregnancy and for each of the trimesters of pregnancy with the changes in Choline across pregnancy.

Supplementary Table 55. Correlations between osmolality levels and PRE to POST changes in creatine across pregnancy.

Osmolality levels	R	p
Trimester 1	.147	.423
Trimester 2	-.207	.227
Trimester 3	.308	.068
Whole pregnancy	.174	.310

Note. Correlation results between mean osmolality levels averaged across whole pregnancy and for each of the trimesters of pregnancy with the changes in Creatine across pregnancy.

Supplementary Table 56. Correlations between osmolality levels and PRE to POST changes in glutamate across pregnancy.

Osmolality levels	R	p
Trimester 1	.287	.111
Trimester 2	-.015	.930
Trimester 3	.431	.009*
Whole pregnancy	.340	.039

Note. Correlation results between mean osmolality levels averaged across whole pregnancy and for each of the trimesters of pregnancy with the changes in glutamate across pregnancy.

Supplementary Table 57. Correlations between osmolality levels and PRE to POST changes in myo-inositol across pregnancy.

Osmolality levels	R	p
Trimester 1	.038	.834
Trimester 2	-.063	.715
Trimester 3	.341	.042
Whole pregnancy	.229	.179

Note. Correlation results between mean osmolality levels averaged across whole pregnancy and for each of the trimesters of pregnancy with the changes in myo-inositol across pregnancy.

Supplementary Table 58. Correlations between osmolality levels and PRE to POST changes in tNAA across pregnancy.

Osmolality levels	R	p
Trimester 1	.215	.236
Trimester 2	.283	.094
Trimester 3	.086	.617
Whole pregnancy	.209	.221

Note. Correlation results between mean osmolality levels averaged across whole pregnancy and for each of the trimesters of pregnancy with the changes in tNAA across pregnancy.

Supplementary Note 2

Stress

Becoming a mother represents a life-changing transition involving many drastic changes in a woman's biology and environment and this can be a stressful period for many mothers. To measure psychological distress, we applied the K10 questionnaire during pregnancy and in the postpartum period. In addition, a questionnaire measuring subjective stress during these periods was applied to obtain an indication of the overall distress experienced by women during their pregnancy and in the postpartum period between delivery and the early postpartum session. Correlation analyses involving each of these measures during pregnancy and the postpartum period rendered no significant results (see Supplementary Tables 59-62), suggesting that stress did not represent a major factor in the induction of the observed neural changes. In addition, for completeness, the main models rendering the changes in brain structure and function were repeated while correcting for each of these stress variables, which indicated that the observed brain changes were also evident when correcting for the degree of stress experienced during pregnancy and the postpartum period (Supplementary Tables 63-70).

Sleep

Furthermore, when becoming a mother, many women experience drastic changes in sleep, especially in the early postpartum period. Therefore, women were asked to keep track of their sleep duration and the number of sleep disruptions, which were used as an indication of the women's sleep quality. An average number of hours of sleep and an average number of sleep disruptions per night in the week preceding the pregnancy and postpartum sessions were included in correlation analyses. In addition, indications of the women's sleep across pregnancy (in terms of the number of hours and sleep disruptions, until week 36 of pregnancy) and the early postpartum period (until the first postpartum session) were included in correlation analyses. All measures of sleep were included in correlation analyses with the observed changes in brain structure and function, which rendered no significant results (Supplementary Tables 71 and 72). In addition, the main models were repeated while correcting for each of these sleep variables, which rendered highly similar results (Supplementary Tables 73-84), suggesting that the women's sleep does not represent a major factor contributing to the observed brain changes.

Duration of Exposure to Postpartum Factors

To further examine the contribution of postpartum factors to the observed changes, correlations were additionally performed with the time between delivery and the Post session, which represents the duration of exposure to postpartum factors until the women's participation in the early postpartum session. These analyses indicated no significant correlations between this variable and the observed changes in brain structure and function (Supplementary Tables 85and 86). Accordingly, models including this variable as a confounding factor also rendered similar results to the main models (Supplementary Tables 87 and 88).

Breastfeeding

Breastfeeding, which involves intense contact with the infant and its own repertoire of hormonal fluctuations, could also be hypothesized to potentially contribute to the observed neural changes. To examine the potential role of breastfeeding, we compared the changes in brain structure and function between women who breastfed their infant and women who did not. These analyses did not render any significant results. In addition, within the group of women who breastfed their children, correlations were performed with the number of feedings per 24 hours in the period of the postpartum session, which also did not render significant results (see Supplementary Tables 89 and 90). Accordingly, including this variable as a confounding factor in the main analyses did not significantly alter the results (see Supplementary Tables 91 and 92).

Interestingly, a supplementary analysis examining the association between the total months of breastfeeding until the Post+1y session with changes in grey matter volume and DMN coherence across the postpartum period revealed a positive correlation between reversal of DMN coherence across the postpartum period and the duration of breastfeeding (Supplementary Tables 93 and 94), suggesting that prolonged breastfeeding may play a role/may stimulate the maintenance of these changes in DMN coherence.

Type of delivery

Finally, to investigate whether the type of delivery plays an important role in the observed brain changes, we compared the changes in brain structure and function between the women who gave birth by means of vaginal delivery to the women who delivered by means of a caesarean section. These comparisons not show significant differences in brain changes based on the type of childbirth.

Supplementary Table 59. Correlations between K10 and PRE to POST changes in DMN coherence.

Measures	R (rho)	P (p rho)
K10 Pregnancy	.002	.992
K10 Postpartum	.119 (-.155)	.472 (.345)

Note. Correlation results between K10 scores acquired during pregnancy and the postpartum period and the observed changes in default mode network coherence. In case of deviations from normality, a non-parametric Spearman's correlation test was performed rather than a Pearson's test. Spearman's rho ("rho") and the p-values for this test ("p rho") are then also reported in the table.

Supplementary Table 60. Correlations between K10 and PRE to POST changes in grey matter volume.

Measures	R	p
K10 Pregnancy	-.12	.580
K10 Postpartum	-.34	.898

Note. Correlation results between K10 scores acquired during pregnancy and the postpartum period and the observed changes in grey matter volume. It should be noted that for these analyses the R cannot be interpreted as a reflection of the direction of the biological effect, since this statistic is based on patterns of brain changes across the whole brain.

Supplementary Table 61. Correlations between subjective stress and PRE to POST changes in DMN coherence.

Measures	R	p
Subjective Stress Pregnancy	-.243	.213
Subjective Stress Postpartum	-.211	.282

Note. Correlation results between the women's subjective stress experienced during pregnancy and the postpartum period and the observed changes in default mode network coherence.

Supplementary Table 62. Correlations between subjective stress and PRE to POST changes in grey matter volume.

Measures	R	p
Subjective Stress Pregnancy	-.24	.710
Subjective Stress Postpartum	-.35	.856

Note. Correlation results between the women's subjective stress experienced during pregnancy and the postpartum period and the observed changes in grey matter volume. It should be noted that for these analyses the R cannot be interpreted as a reflection of the direction of the biological effect, since this statistic is based on patterns of brain changes across the whole brain.

Supplementary Table 63. Changes in DMN across pregnancy (PRE to POST) corrected for the K10 scale in the pregnancy period.

Contrast	Regions	MNI coordinates			T	P (FWE-corrected)	Cluster Size (mm^3)
		x	y	z			
Increases	L Cuneus, R Cuneus	6	-84	18	6.44	0.001	243
Decreases	-						

Note. Increases or decreases in within-network connectivity in the DMN in primiparous women between the pre-conception and the early postpartum session corrected for the K10 scale in the pregnancy period. Results are reported at a statistical threshold of p<0.05 FWE-corrected. L = left, R = right.

Supplementary Table 64. Changes in DMN across pregnancy (PRE to POST) corrected for the K10 scale in the early postpartum period.

Contrast	Regions	MNI coordinates			T	P (FWE-corrected)	Cluster Size (mm^3)
		x	y	z			
Increases	R Cuneus	9	-81	18	5.55	0.006	108
Decreases	-						

Note. Increases or decreases in within-network connectivity in the DMN in primiparous women between the pre-conception and the early postpartum session corrected for the K10 scale in the early postpartum period. Results are reported at a statistical threshold of p<0.05 FWE-corrected. L = left, R = right.

Supplementary Table 65. Changes in DMN across pregnancy (PRE to POST) corrected for the degree of stress during pregnancy.

Contrast	Regions	MNI coordinates			T	P (FWE-corrected)	Cluster Size (mm^3)
		x	y	z			
Increases	R Cuneus	6	-81	15	5.53	0.019	81
Decreases	-						

Note. Increases or decreases in within-network connectivity in the DMN in primiparous women between the pre-conception and the early postpartum session corrected for stress during pregnancy. Results are reported at a statistical threshold of p<0.05 FWE-corrected. L = left, R = right.

Supplementary Table 66. Changes in DMN across pregnancy (PRE to POST) corrected for the degree of stress since birth.

Contrast	Regions	MNI coordinates			T	P (FWE-corrected)	Cluster Size (mm^3)
		x	y	z			
Increases	R Cuneus	6	-81	15	5.52	0.019	81
Decreases	-						

Note. Increases or decreases in within-network connectivity in the DMN in primiparous women between the pre-conception and the early postpartum session corrected for the amount of stress since birth. Results are reported at a statistical threshold of p<0.05 FWE-corrected. L = left, R = right.

Supplementary Table 67. Changes in grey matter volume across pregnancy (PRE to POST) corrected for the K10 scale in the pregnancy period.

Contrasts	Regions	MNI coordinates *x*	*y*	*z*	*T*	*p* (FWE-corrected)	Cluster Size (mm³)
Increases	-						
Decreases	LR Superior, Middle, Inferior Frontal Gyrus, LR Superior Medial Frontal Cortex, LR Orbitofrontal Cortex, LR Superior, Middle, Inferior Temporal Gyrus, LR Precuneus, LR Superior, Inferior Parietal Gyrus, LR Anterior, Middle, Posterior Cingulate Cortex, LR Fusiform Gyrus, LR Precentral Gyrus, LR Postcentral Gyrus, LR Superior, Middle, Inferior Occipital Gyrus, LR Angular gyrus, LR Supplementary Motor Area, LR Supramarginal Gyrus, LR Anterior, Posterior Cerebellum, LR Insula, LR Superior, Middle Temporal Pole, LR Rolandic Operculum, LR Lingual Gyrus, LR Thalamus, LR Caudate Nucleus, LR Putamen, LR Calcarine Sulcus, LR Gyrus Rectus, LR Hippocampus, LR Parahippocampal Gyrus, LR Cuneus, LR Heschl's Gyrus, Vermis, LR Olfactory Cortex, LR Pallidum, LR Paracentral Lobule, LR Superior Temporal Sulcus, LR Temporo-Parietal Junction	-66<br>-68<br>-36	-27<br>-41<br>24	-15<br>-14<br>-17	18.49<br>16.39<br>15.66	<0.001<br><0.001<br><0.001	1223498
	L Precuneus, L Postcentral Gyrus, L Superior Parietal Gyrus	-11	-56	84	7.28	0.001	1553
	R Postcentral Gyrus	17	-35	90	7.21	0.001	753
	R Postcentral Gyrus, R Superior Parietal Gyrus	18	-51	81	6.13	0.010	273
	R Posterior Cerebellum	60	-66	-45	5.85	0.019	74

Note. Increases or decreases in regional grey matter volumes in primiparous women between the pre-conception and the early postpartum session corrected for K10 in the pregnancy session. Statistics are extracted from one-sample t-tests performed within the framework of an SPM12 General Linear Model and are one-sided (as is standard in SPM). Results are reported at a statistical threshold of $p<0.05$ FWE-corrected. L = left, R = right.

Supplementary Table 68. Changes in grey matter volume across pregnancy (PRE to POST) corrected for the K10 scale in the postpartum period.

Contrasts	Regions	MNI coordinates			*T*	*p* (FWE-corrected)	Cluster Size (mm^3)
		x	*y*	*z*			
Increases	-						
Decreases	LR Superior, Middle, Inferior Frontal Gyrus, LR Superior Medial Frontal Cortex, LR Orbitofrontal Cortex, LR Superior, Middle, Inferior Temporal Gyrus, LR Precuneus, LR Superior, Inferior Parietal Gyrus, LR Anterior, Middle, Posterior Cingulate Cortex, LR Fusiform Gyrus, LR Precentral Gyrus, LR Postcentral Gyrus, LR Superior, Middle, Inferior Occipital Gyrus, LR Angular gyrus, LR Supplementary Motor Area, LR Supramarginal Gyrus, LR Anterior, Posterior Cerebellum, LR Insula, LR Superior, Middle Temporal Pole, LR Rolandic Operculum, LR Lingual Gyrus, LR Thalamus, LR Caudate Nucleus, LR Putamen, LR Calcarine Sulcus, LR Gyrus Rectus, LR Hippocampus, LR Parahippocampal Gyrus, LR Cuneus, LR Heschl's Gyrus, Vermis, LR Olfactory Cortex, LR Pallidum, LR Paracentral Lobule, LR Superior Temporal Sulcus, LR Temporo-Parietal Junction	-65<br>-65<br>65	-29<br>-51<br>-33	-15<br>-18<br>-17	18.11<br>17.64<br>15.46	<0.001<br><0.001<br><0.001	1273847
	R Postcentral Gyrus, R Superior Parietal Gyrus, R Precentral Gyrus	17	-35	89	6.78	0.001	1499
	L Superior Parietal Gyrus, L Precuneus, L Postcentral Gyrus	-14	-57	83	6.17	0.006	628

Note. Increases or decreases in regional grey matter volumes in primiparous women between the pre-conception and the early postpartum session corrected for K10 of the early postpartum session. Statistics are extracted from one-sample t-tests performed within the framework of an SPM12 General Linear Model and are one-sided (as is standard in SPM). Results are reported at a statistical threshold of $p<0.05$ FWE-corrected. L = left, R = right.

Supplementary Table 69. Changes in grey matter volume across pregnancy (PRE to POST) corrected for the degree of stress during pregnancy

Contrasts	Regions	MNI coordinates *x*	*y*	*z*	*T*	*p* (FWE-corrected)	Cluster Size (mm^3)
Increases	-						
Decreases	LR Superior, Middle, Inferior Frontal Gyrus, LR Superior Medial Frontal Cortex, LR Orbitofrontal Cortex, LR Superior, Middle, Inferior Temporal Gyrus, LR Precuneus, LR Superior, Inferior Parietal Gyrus, LR Anterior, Middle, Posterior Cingulate Cortex, LR Fusiform Gyrus, LR Precentral Gyrus, LR Postcentral Gyrus, LR Superior, Middle Occipital Gyrus, L Inferior Occipital Gyrus, LR Angular gyrus, LR Supplementary Motor Area, LR Supramarginal Gyrus, LR Anterior, Cerebellum, LR Insula, LR Superior, Middle Temporal Pole, LR Rolandic Operculum, LR Lingual Gyrus, LR Thalamus, LR Caudate Nucleus, LR Putamen, LR Calcarine Sulcus, LR Gyrus Rectus, LR Hippocampus, LR Parahippocampal Gyrus, LR Cuneus, LR Heschl's Gyrus, Vermis, LR Olfactory Cortex, LR Pallidum, LR Paracentral Lobule, LR Superior Temporal Sulcus, LR Temporo-Parietal Junction	-63<br>-66<br>53	-42<br>-29<br>-35	-20<br>-15<br>3	19.53<br>18.93<br>15.40	<0.001<br><0.001<br><0.001	928172
	Vermis, L Anterior Cerebellum	3	-47	-15	6.89	0.006	270
	R Calcarine Sulcus	12	-75	11	6.61	0.010	192
	L Posterior Cerebellum	-36	-86	-47	6.60	0.010	186
	R Supramarginal Gyrus, R Postcentral Gyrus, R Rolandic Operculum	59	-17	27	6.54	0.011	250
	L Inferior Temporal Gyrus	-36	3	-39	6.48	0.013	216
	R Posterior Cerebellum	41	-30	-39	6.36	0.017	30
	L Anterior Cingulate Cortex	-2	0	27	6.17	0.025	81
	R Posterior Cerebellum	41	-84	-48	6.06	0.031	34

Note. Increases or decreases in regional grey matter volumes in primiparous women between the pre-conception and the early postpartum session corrected for subjective degree of stress during pregnancy. Statistics are extracted from one-sample t-tests performed within the framework of an SPM12 General Linear Model and are one-sided (as is standard in SPM). Results are reported at a statistical threshold of $p<0.05$ FWE-corrected. L = left, R = right.

Supplementary Table 70. Changes in grey matter volume across pregnancy (PRE to POST) corrected for the degree of stress since birth

Contrasts	Regions	MNI coordinates x	y	z	*T*	*p* (FWE-corrected)	Cluster Size (mm^3)
Increases	-						
Decreases	LR Superior, Middle, Inferior Frontal Gyrus, LR Superior Medial Frontal Cortex, LR Orbitofrontal Cortex, LR Superior, Middle, Inferior Temporal Gyrus, LR Precuneus, LR Superior, Inferior Parietal Gyrus, LR Anterior, Middle, Posterior Cingulate Cortex, LR Fusiform Gyrus, LR Precentral Gyrus, LR Postcentral Gyrus, LR Superior, Middle Occipital Gyrus, L Inferior Occipital Gyrus, LR Angular gyrus, LR Supplementary Motor Area, LR Supramarginal Gyrus, LR Anterior, Posterior Cerebellum, LR Insula, LR Superior, Middle Temporal Pole, LR Rolandic Operculum, LR Lingual Gyrus, LR Thalamus, LR Caudate Nucleus, LR Putamen, LR Calcarine Sulcus, LR Gyrus Rectus, LR Hippocampus, LR Parahippocampal Gyrus, LR Cuneus, LR Heschl's Gyrus, Vermis, LR Olfactory Cortex, LR Pallidum, LR Paracentral Lobule, LR Superior Temporal Sulcus, LR Temporo-Parietal Junction	-63	-42	-20	19.16	<0.001	923400
		-66	-29	-15	18.94	<0.001	
		53	-35	3	15.00	<0.001	
	L Posterior Cerebellum	-36	-86	-47	6.64	0.009	196
	Vermis	3	-47	-14	6.60	0.010	155
	R Calcarine Sulcus	12	-75	11	6.57	0.011	189
	R Supramarginal Gyrus, R Postcentral Gyrus	59	-17	27	6.49	0.013	213
	L Inferior Temporal Gyrus	-36	3	-39	6.38	0.016	179
	R Posterior Cerebellum	39	-30	-38	6.22	0.022	17
	R Posterior Cerebellum	41	-84	-48	6.05	0.031	41

Note. Increases or decreases in regional grey matter volumes in primiparous women between the pre-conception and the early postpartum session corrected for the subjective degree of stress since birth. Statistics are extracted from one-sample t-tests performed within the framework of an SPM12 General Linear Model and are one-sided (as is standard in SPM). Results are reported at a statistical threshold of $p<0.05$ FWE-corrected. P-value at peak voxel (whole-brain FWE corrected) is reported. L = left, R = right.

Supplementary Table 71. Correlations between sleep quality and PRE to POST changes in grey matter volume.

Measures	R	p
Average hours of sleep week preceding pregnancy session	-.46	.965
Average number of sleep disruptions week preceding pregnancy session	-.08	.521
Average hours of sleep week preceding postpartum session	.16	.166
Average number of sleep disruptions week preceding postpartum session	-.25	.831
Indication of sleep quality (hours of sleep) across the first 36 weeks of pregnancy	-.04	.445
Indication of sleep quality (sleep disruptions) across the first 36 weeks of pregnancy	.11	.194
Indication of sleep quality (hours of sleep) between delivery and the Post session	.04	.319
Indication of sleep quality (sleep disruptions) between delivery and the Post session	-.03	.436

Note. Correlation results between variables representing the women's sleep quality during pregnancy and the postpartum period and the observed changes in grey matter volume. It should be noted that for these analyses the R cannot be interpreted as a reflection of the direction of the biological effect, since this statistic is based on patterns of brain changes across the whole brain.

Supplementary Table 72. Correlations between sleep quality and PRE to POST changes in DMN coherence.

Measures	R (rho)	p (rho p)
Average hours of sleep week preceding pregnancy session	.071 (-0.006)	.683 (.971)
Average number of sleep disruptions week preceding pregnancy session	-.155 (-.228)	.374 (.188)
Average hours of sleep week preceding postpartum session	-.202	.212
Average number of sleep disruptions week preceding postpartum session	-.075 (.010)	.647 (.951)
Indication of sleep quality (hours of sleep) across the first 36 weeks of pregnancy	-.092	.601
Indication of sleep quality (sleep disruptions) across the first 36 weeks of pregnancy	-.188 (-.232)	.280 (.179)
Indication of sleep quality (hours of sleep) between delivery and the Post session	-.102	.560
Indication of sleep quality (sleep disruptions) between delivery and the Post session	-.028 (.077)	.871 (.654)

Note. Correlation results between variables representing the women's sleep quality during pregnancy and the postpartum period and the observed changes in default mode network coherence. In case of deviations from normality, a non-parametric Spearman's correlation test was performed rather than a Pearson's test. Spearman's rho ("rho") and the p-values for this test ("p rho") are then also reported in the table.

Supplementary Table 73. Changes in DMN across pregnancy (PRE to POST) corrected for hours of sleep in the week before the pregnancy session.

Contrast	Regions	MNI coordinates			*T*	*P* (FWE-corrected)	Cluster Size (mm³)
		x	*y*	*z*			
Increases	L Cuneus, R Cuneus	6	-84	18	6.32	0.001	216
Decreases	-						

Note. Increases or decreases in within-network connectivity in the DMN in primiparous women between the pre-conception and the early postpartum session corrected for the average number of hours of sleep per night in the week before the pregnancy session. Results are reported at a statistical threshold of p<0.05 FWE-corrected. . L = left, R = right.

Supplementary Table 74. Changes in DMN across pregnancy (PRE to POST) corrected for hours of sleep in the week before the early postpartum session.

Contrast	Regions	MNI coordinates			*T*	*P* (FWE-corrected)	Cluster Size (mm³)
		x	*y*	*z*			
Increases	L Cuneus, R Cuneus	6	-84	18	5.59	0.005	135
Decreases	-						

Note. Increases or decreases in within-network connectivity in the DMN in primiparous women between the pre-conception and the early postpartum session corrected for the average number of hours of sleep per night in the week before the early postpartum session. Results are reported at a statistical threshold of p<0.05 FWE-corrected. L = left, R = right.

Supplementary Table 75. Changes in DMN across pregnancy (PRE to POST) corrected for sleep disruptions in the week before the pregnancy session.

Contrast	Regions	MNI coordinates			*T*	*P* (FWE-corrected)	Cluster Size (mm³)
		x	*y*	*z*			
Increases	L Cuneus, R Cuneus	6	-84	18	6.31	0.001	216
Decreases	-						

Note. Increases or decreases in within-network connectivity in the DMN in primiparous women between the pre-conception and the early postpartum session corrected for the average number of sleep disruptions per night in the week before the pregnancy session. Results are reported at a statistical threshold of p<0.05 FWE-corrected. L = left, R = right.

Supplementary Table 76. Changes in DMN across pregnancy (PRE to POST) corrected for sleep disruptions in the week before the early postpartum session.

Contrast	Regions	MNI coordinates			*T*	*P* (FWE-corrected)	Cluster Size (mm³)
		x	*y*	*z*			
Increases	L Cuneus, R Cuneus	6	-84	18	5.52	0.006	135
Decreases	-						

Note. Increases or decreases in within-network connectivity in the DMN in primiparous women between the pre-conception and the early postpartum session corrected for the average number of sleep disruptions per night in the week before the early postpartum session. Results are reported at a statistical threshold of p<0.05 FWE-corrected. . L = left, R = right.

Supplementary Table 77. Changes in DMN across pregnancy (PRE to POST) corrected for hours of sleep between birth and the early postpartum session.

Contrast	Regions	MNI coordinates			*T*	*P* (FWE-corrected)	Cluster Size (mm³)
		x	*y*	*z*			
Increases	L Cuneus, R Cuneus	6	-84	18	6.03	0.003	216
Decreases	-						

Note. Increases or decreases in within-network connectivity in the DMN in primiparous women between the pre-conception and the early postpartum session corrected for the average number of hours of sleep per night between birth and the early postpartum session. Results are reported at a statistical threshold of p<0.05 FWE-corrected. . L = left, R = right.

Supplementary Table 78. Changes in DMN across pregnancy (PRE to POST) corrected for the average number of sleep disruptions per night between birth and the early postpartum session

Contrast	Regions	MNI coordinates *x*	*y*	*z*	*T*	*P* (FWE-corrected)	Cluster Size (mm³)
Increases	L Cuneus, R Cuneus	6	-84	18	5.96	0.003	216
Decreases	-						

Note. Increases or decreases in within-network connectivity in the DMN in primiparous women between the pre-conception and the early postpartum session corrected for the average number of sleep disruptions per night between birth and the early postpartum session. Results are reported at a statistical threshold of p<0.05 FWE-corrected. L = left, R = right.

Supplementary Table 79. Changes in grey matter volume across pregnancy (PRE to POST) corrected for hours sleep in the week before the pregnancy session.

Contrasts	Regions	MNI coordinates *x*	*y*	*z*	*T*	*p* (FWE-corrected)	Cluster Size (mm³)
Increases	-						
Decreases	LR Superior, Middle, Inferior Frontal Gyrus, LR Superior Medial Frontal Cortex, LR Orbitofrontal Cortex, LR Superior, Middle, Inferior Temporal Gyrus, LR Precuneus, LR Superior, Inferior Parietal Gyrus, LR Anterior, Middle, Posterior Cingulate Cortex, LR Fusiform, LR Precentral Gyrus, LR Postcentral Gyrus, LR Superior, Middle, Inferior Occipital Gyrus, LR Angular gyrus, LR Supplementary Motor Area, LR Supramarginal Gyrus, LR Anterior, Posterior Cerebellum, LR Insula, LR Superior, Middle Temporal Pole, LR Rolandic Operculum, LR Lingual Gyrus, LR Thalamus, LR Caudate Nucleus, LR Putamen, LR Calcarine Sulcus, LR Gyrus Rectus, LR Hippocampus, LR Parahippocampal Gyrus, LR Cuneus, LR Heschl's Gyrus, Vermis, LR Olfactory Cortex, LR Pallidum, LR Paracentral Lobule, LR Superior Temporal Sulcus, LR Temporo-Parietal Junction	-65<br>-63<br>-36	-29<br>-41<br>26	-15<br>-17<br>-12	18.56<br>17.91<br>16.17	<0.001<br><0.001<br><0.001	1299375
	R Postcentral Gyrus	17	-35	90	7.08	0.001	746
	L Precuneus, L Superior Parietal Gyrus	-14	-57	83	6.32	0.006	638
	R Posterior Cerebellum	44	-87	-47	6.17	0.009	338
	R Postcentral Gyrus, R Superior Parietal Gyrus	18	-50	80	5.99	0.014	189
	R Posterior Cerebellum	60	-66	-45	5.92	0.016	74

Note. Increases or decreases in regional grey matter volumes in primiparous women between the pre-conception and the early postpartum session corrected for the average number of hours of sleep per night in the week before the pregnancy session. Statistics are extracted from one-sample t-tests performed within the framework of an SPM12 General Linear Model and are one-sided (as is standard in SPM). Results are reported at a statistical threshold of p<0.05 FWE-corrected. P-value at peak voxel (whole-brain FWE corrected) is reported. L = left, R = right.

Supplementary Table 80. Changes in grey matter volume across pregnancy (PRE to POST) corrected for hours sleep in the week before the early postpartum session.

Contrasts	Regions	MNI coordinates x	y	z	*T*	*p* (FWE-corrected)	Cluster Size (mm³)
Increases	-						
Decreases	LR Superior, Middle, Inferior Frontal Gyrus, LR Superior Medial Frontal Cortex, LR Orbitofrontal Cortex, LR Superior, Middle, Inferior Temporal Gyrus, LR Precuneus, LR Superior, Inferior Parietal Gyrus, LR Anterior, Middle, Posterior Cingulate Cortex, LR Fusiform, LR Precentral Gyrus, LR Postcentral Gyrus, LR Superior, Middle, Inferior Occipital Gyrus, LR Angular gyrus, LR Supplementary Motor Area, LR Supramarginal Gyrus, LR Anterior, Posterior Cerebellum, LR Insula, LR Superior, Middle Temporal Pole, LR Rolandic Operculum, LR Lingual Gyrus, LR Thalamus, LR Caudate Nucleus, LR Putamen, LR Calcarine Sulcus, LR Gyrus Rectus, LR Hippocampus, LR Parahippocampal Gyrus, LR Cuneus, LR Heschl's Gyrus, Vermis, LR Olfactory Cortex, LR Pallidum, LR Paracentral Lobule, LR Superior Temporal Sulcus, LR Temporo-Parietal Junction	-65<br>-65<br>63	-41<br>-29<br>-32	-17<br>-15<br>-15	18.56<br>17.91<br>16.17	<0.001<br><0.001<br><0.001	1343770
	R Superior Parietal Gyrus, R Precentral Gyrus, R Postcentral Gyrus, R Paracentral Lobule	17	-35	89	7.01	0.001	1755
	L Precuneus, L Superior Parietal Gyrus, L Postcentral Gyrus	-14	-57	83	6.29	0.004	1158
	L Paracentral Lobule	-3	-15	86	5.81	0.013	341

Note. Increases or decreases in regional grey matter volumes in primiparous women between the pre-conception and the early postpartum session corrected for the average number of hours of sleep per night in the week before the early postpartum session. . Statistics are extracted from one-sample t-tests performed within the framework of an SPM12 General Linear Model and are one-sided (as is standard in SPM). Results are reported at a statistical threshold of $p<0.05$ FWE-corrected. P-value at peak voxel (whole-brain FWE corrected) is reported. L = left, R = right.

Supplementary Table 81. Changes in grey matter volume across pregnancy (PRE to POST) corrected for sleep disruptions in the week before the pregnancy session.

Contrasts	Regions	MNI coordinates			*T*	*p* (FWE-corrected)	Cluster Size (mm^3)
		x	*y*	*z*			
Increases	-						
Decreases	LR Superior, Middle, Inferior Frontal Gyrus, LR Superior Medial Frontal Cortex, LR Orbitofrontal Cortex, LR Superior, Middle, Inferior Temporal Gyrus, LR Precuneus, LR Superior, Inferior Parietal Gyrus, LR Anterior, Middle, Posterior Cingulate Cortex, LR Fusiform, LR Precentral Gyrus, LR Postcentral Gyrus, LR Superior, Middle, Inferior Occipital Gyrus, LR Angular gyrus, LR Supplementary Motor Area, LR Supramarginal Gyrus, LR Anterior, Posterior Cerebellum, LR Insula, LR Superior, Middle Temporal Pole, LR Rolandic Operculum, LR Lingual Gyrus, LR Thalamus, LR Caudate Nucleus, LR Putamen, LR Calcarine Sulcus, LR Gyrus Rectus, LR Hippocampus, LR Parahippocampal Gyrus, LR Cuneus, LR Heschl's Gyrus, Vermis, LR Olfactory Cortex, LR Pallidum, LR Paracentral Lobule, LR Superior Temporal Sulcus, LR Temporo-Parietal Junction	-66<br>-63<br>-36	-27<br>-42<br>26	-15<br>-18<br>-17	19.93<br>17.05<br>16.28	<0.001<br><0.001<br><0.001	1227214
	R Postcentral Gyrus	17	-35	90	7.18	0.001	699
	L Precuneus, L Superior Parietal Gyrus	-14	-57	83	6.38	0.005	786
	R Postcentral Gyrus, R Superior Parietal Gyrus	17	-50	81	6.25	0.007	304
	R Posterior Cerebellum	44	-86	-53	6.14	0.010	321
	R Posterior Cerebellum	60	-66	-45	5.85	0.019	182

Note. Increases or decreases in regional grey matter volumes in primiparous women between the pre-conception and the early postpartum session corrected for the average number of sleep disruptions per night in the week before the pregnancy session. Statistics are extracted from one-sample t-tests performed within the framework of an SPM12 General Linear Model and are one-sided (as is standard in SPM). Results are reported at a statistical threshold of $p<0.05$ FWE-corrected. P-value at peak voxel (whole-brain FWE corrected) is reported. L = left, R = right.

Supplementary Table 82. Changes in grey matter volume across pregnancy (PRE to POST) corrected for sleep disruptions in the week before the early postpartum session.

Contrasts	Regions	MNI coordinates *x*	*y*	*z*	*T*	*p* (FWE-corrected)	Cluster Size (mm^3)
Increases	-						
Decreases	LR Superior, Middle, Inferior Frontal Gyrus, LR Superior Medial Frontal Cortex, LR Orbitofrontal Cortex, LR Superior, Middle, Inferior Temporal Gyrus, LR Precuneus, LR Superior, Inferior Parietal Gyrus, LR Anterior, Middle, Posterior Cingulate Cortex, LR Fusiform, LR Precentral Gyrus, LR Postcentral Gyrus, LR Superior, Middle, Inferior Occipital Gyrus, LR Angular gyrus, LR Supplementary Motor Area, LR Supramarginal Gyrus, LR Anterior, Posterior Cerebellum, LR Insula, LR Superior, Middle Temporal Pole, LR Rolandic Operculum, LR Lingual Gyrus, LR Thalamus, LR Caudate Nucleus, LR Putamen, LR Calcarine Sulcus, LR Gyrus Rectus, LR Hippocampus, LR Parahippocampal Gyrus, LR Cuneus, LR Heschl's Gyrus, Vermis, LR Olfactory Cortex, LR Pallidum, LR Paracentral Lobule, LR Superior Temporal Sulcus, LR Temporo-Parietal Junction	-65<br>-63<br>63	-29<br>-41<br>-32	-15<br>-18<br>-17	18.77<br>18.60<br>16.44	<0.001<br><0.001<br><0.001	1321907
	R Superior Parietal Gyrus, R Precentral Gyrus, R Postcentral Gyrus, R Paracentral Lobule	17	-35	89	7.13	<0.001	1944
	L Precuneus, L Superior Parietal Gyrus, L Postcentral Gyrus	-14	-57	83	6.28	0.004	1232
	L Paracentral Lobule	-6	-15	89	5.83	0.012	358
	R Postcentral Gyrus	72	-6	30	5.44	0.033	4

Note. Increases or decreases in regional grey matter volumes in primiparous women between the pre-conception and the early postpartum session corrected for the average number of sleep disruptions per night in the week before the early postpartum session. Statistics are extracted from one-sample t-tests performed within the framework of an SPM12 General Linear Model and are one-sided (as is standard in SPM). Results are reported at a statistical threshold of $p<0.05$ FWE-corrected. P-value at peak voxel (whole-brain FWE corrected) is reported. L = left, R = right.

Supplementary Table 83. Changes in grey matter volume across pregnancy (PRE to POST) corrected for hours sleep between birth and the early postpartum session.

Contrasts	Regions	MNI coordinates *x*	*y*	*z*	*T*	*p* (FWE-corrected)	Cluster Size (mm³)
Increases	-						
Decreases	LR Superior, Middle, Inferior Frontal Gyrus, LR Superior Medial Frontal Cortex, LR Orbitofrontal Cortex, LR Superior, Middle, Inferior Temporal Gyrus, LR Precuneus, LR Superior, Inferior Parietal Gyrus, LR Anterior, Middle, Posterior Cingulate Cortex, LR Fusiform, LR Precentral Gyrus, LR Postcentral Gyrus, LR Superior, Middle, Inferior Occipital Gyrus, LR Angular gyrus, LR Supplementary Motor Area, LR Supramarginal Gyrus, LR Anterior, Posterior Cerebellum, LR Insula, LR Superior, Middle Temporal Pole, LR Rolandic Operculum, LR Lingual Gyrus, LR Thalamus, LR Caudate Nucleus, LR Putamen, LR Calcarine Sulcus, LR Gyrus Rectus, LR Hippocampus, LR Parahippocampal Gyrus, LR Cuneus, LR Heschl's Gyrus, Vermis, LR Olfactory Cortex, LR Pallidum, LR Paracentral Lobule, LR Superior Temporal Sulcus, LR Temporo-Parietal Junction	-63	-39	-18	18.43	<0.001	1162229
		-65	-29	-14	17.83	<0.001	
		-36	23	-14	14.49	<0.001	
	R Postcentral Gyrus	17	-35	89	7.29	0.001	604
	R Postcentral Gyrus, R Superior Parietal Gyrus	20	-48	83	6.04	0.012	132
	R Posterior Cerebellum	60	-66	-45	5.65	0.031	27

Note. Increases or decreases in regional grey matter volumes in primiparous women between the pre-conception and the early postpartum session corrected for the average number of hours of sleep per night between birth and the early postpartum session. Statistics are extracted from one-sample t-tests performed within the framework of an SPM12 General Linear Model and are one-sided (as is standard in SPM). Results are reported at a statistical threshold of p<0.05 FWE-corrected. P-value at peak voxel (whole-brain FWE corrected) is reported. L = left, R = right.

Supplementary Table 84. Changes in grey matter volume across pregnancy (PRE to POST) corrected for sleep disruptions between birth and the early postpartum session.

Contrasts	Regions	MNI coordinates *x*	*y*	*z*	*T*	*p* (FWE-corrected)	Cluster Size (mm³)
Increases	-						
Decreases	LR Superior, Middle, Inferior Frontal Gyrus, LR Superior Medial Frontal Cortex, LR Orbitofrontal Cortex, LR Superior, Middle, Inferior Temporal Gyrus, LR Precuneus, LR Superior, Inferior Parietal Gyrus, LR Anterior, Middle, Posterior Cingulate Cortex, LR Fusiform, LR Precentral Gyrus, LR Postcentral Gyrus, LR Superior, Middle, Inferior Occipital Gyrus, LR Angular gyrus, LR Supplementary Motor Area, LR Supramarginal Gyrus, LR Anterior, Posterior Cerebellum, LR Insula, LR Superior, Middle Temporal Pole, LR Rolandic Operculum, LR Lingual Gyrus, LR Thalamus, LR Caudate Nucleus, LR Putamen, LR Calcarine Sulcus, LR Gyrus Rectus, LR Hippocampus, LR Parahippocampal Gyrus, LR Cuneus, LR Heschl's Gyrus, Vermis, LR Olfactory Cortex, LR Pallidum, LR Paracentral Lobule, LR Superior Temporal Sulcus, LR Temporo-Parietal Junction	-63	-41	-18	19.07	<0.001	1182546
		-65	-27	-15	18.53	<0.001	
		63	-32	-17	16.47	<0.001	
	R Postcentral Gyrus, R Superior Parietal Gyrus	17	-35	89	7.05	0.001	888
	R Posterior Cerebellum	44	-87	-47	6.11	0.009	311
	L Precuneus, L Superior Parietal Gyrus	-14	-56	83	5.94	0.014	371
	R Posterior Cerebellum	60	-66	-45	5.86	0.017	115

Note. Increases or decreases in regional grey matter volumes in primiparous women between the pre-conception and the early postpartum session corrected for the average number of sleep disruptions per night between birth and the early postpartum session. Statistics are extracted from one-sample t-tests performed within the framework of an SPM12 General Linear Model and are one-sided (as is standard in SPM). Results are reported at a statistical threshold of $p<0.05$ FWE-corrected. P-value at peak voxel (whole-brain FWE corrected) is reported. L = left, R = right.

Supplementary Table 85. Correlation between duration exposure to postpartum factors and grey matter volume.

Measures	R	p
Duration exposure postpartum factors	-.01	.940

Note. Correlation results between variables representing the duration of exposure to postpartum factors and the observed changes in grey matter volume. It should be noted that for these analyses the R cannot be interpreted as a reflection of the direction of the biological effect, since this statistic is based on patterns of brain changes across the whole brain.

Supplementary Table 86. Correlation between duration exposure to postpartum factors and DMN coherence.

Measures	R	p
Duration exposure postpartum factors	-.08	.524

Note. Correlation results between variables representing the duration of exposure to postpartum factors and the observed changes in default mode network coherence.

Supplementary Table 87. Changes in grey matter volume (PRE to POST) corrected for time between birth and post session.

Contrasts	Regions	MNI coordinates			T	p (FWE-corrected)	Cluster Size (mm³)
		x	y	z			
Increase	-						
Decrease	LR Superior, Middle, Inferior Frontal Gyrus, LR Superior Medial Frontal Cortex, LR Superior, Middle, Inferior Temporal Gyrus, LR Superior Temporal Sulcus, LR Temporal Pole, LR Precuneus, LR Anterior, Middle, Posterior Cingulate Cortex, LR Fusiform, LR Angular Gyrus, LR Supramarginal Gyrus, LR Temporo-Parietal Junction, LR Supplementary Motor Area, LR Anterior, Posterior Cerebellum, LR Insula, LR Orbitofrontal Cortex, LR Precentral Gyrus, LR Postcentral Gyrus, LR Putamen, LR Calcarine Sulcus, LR Gyrus Rectus, LR Hippocampus LR Thalamus, LR Parahippocampal Gyrus, LR Cuneus, LR Olfactory, Vermis, Pallidum, LR Paracentral Lobule, LR Superior, Middle, Inferior Occipital Gyrus, LR Superior, Inferior Parietal Lobule, LR Lingual Gyrus, LR Caudate	-62	-32	-12	21.07	<0.001	3461124
		-36	24	-10	18.33	<0.001	
		-28	10	54	17.76	<0.001	
	R Anterior Cerebellum	42	-80	-48	6.17	0.032	9
	R Postcentral Gyrus, R Superior Parietal Lobule	15	-33	82	6.17	0.033	11
		18	-50	78	6.16	0.033	8

Note. One sample *t*-test to explore the direction of change in grey matter volume in primiparous (PRG) participants across Pre and Post1 sessions, while adjusting for the days between birth and Post session. . Statistics are extracted from one-sample t-tests performed within the framework of an SPM12 General Linear Model and are one-sided (as is standard in SPM). Results are reported at a statistical threshold of p<0.05 FWE-corrected. P-value at peak voxel (whole-brain FWE corrected) is reported. L = left, R = right.

Supplementary Table 88. Changes in DMN across pregnancy (PRE to POST) corrected for time between birth and post session.

Contrast	Regions	MNI coordinates			T	P (FWE-corrected)	Cluster Size (mm³)
		x	y	z			
Increases	L Cuneus, R Cuneus	9	-84	15	6.25	0.006	216
Decreases	-						

Note. Increases or decreases in within-network connectivity in the DMN in primiparous women between the pre-conception and the early postpartum session corrected for the duration of exposure to postpartum factors (the number of days between birth and the post session). Results are reported at a statistical threshold of p<0.05 FWE-corrected. L = left, R = right.

Supplementary Table 89. Correlation between breastfeeding and PRE to POST changes in grey matter volume.

Measures	R	p
Number of feedings per 24 hours	-.32	.819

Note. Correlation results between the number of feedings per 24 hours in the women who breastfed their children and the observed changes in grey matter volume. It should be noted that for these analyses the R cannot be interpreted as a reflection of the direction of the biological effect, since this statistic is based on patterns of brain changes across the whole brain.

Supplementary Table 90. Correlation between breastfeeding and PRE to POST changes in DMN coherence.

Measures	R (rho)	P (rho p)
Number of feedings per 24 hours	-.054 (-.060)	.775 (.755)

Note. Correlation results between the number of feedings per 24 hours in the women who breastfed their children and the observed changes default mode network coherence. In case of deviations from normality, a non-parametric Spearman's correlation test was performed rather than a Pearson's test. Spearman's rho ("rho") and the p-values for this test ("p rho") are then also reported in the table.

Supplementary Table 91. Changes in grey matter volume across pregnancy (PRE to POST) corrected for the number of feedings per 24 hours in breastfeeding mothers.

Contrasts	Regions	MNI coordinates *x*	*y*	*z*	*T*	*p* (FWE-corrected)	Cluster Size (mm^3)
Increases	-						
Decreases	LR Superior, Middle, Inferior Frontal Gyrus, LR Superior Medial Frontal Cortex, LR Orbitofrontal Cortex, LR Superior, Middle, Inferior Temporal Gyrus, LR Precuneus, LR Superior, Inferior Parietal Gyrus, LR Anterior, Middle, Posterior Cingulate Cortex, LR Fusiform Gyrus, LR Precentral Gyrus, LR Postcentral Gyrus, LR Superior, Middle Inferior Occipital Gyrus, LR Angular gyrus, LR Supplementary Motor Area, LR Supramarginal Gyrus, LR Anterior, Posterior Cerebellum, LR Insula, LR Superior, Middle Temporal Pole, LR Rolandic Operculum, LR Lingual Gyrus, LR Thalamus, LR Caudate Nucleus, LR Putamen, LR Calcarine Sulcus, LR Gyrus Rectus, LR Hippocampus, LR Parahippocampal Gyrus, LR Cuneus, LR Heschl's Gyrus, Vermis, LR Olfactory Cortex, LR Pallidum, LR Paracentral Lobule, LR Superior Temporal Sulcus, LR Temporo-Parietal Junction	-68<br>-66<br>-36	-41<br>-27<br>23	-14<br>-14<br>-15	16.49<br>16.33<br>13.73	<0.001<br><0.001<br><0.001	934777
	R Postcentral Gyrus	17	-35	90	6.71	0.005	365
	Vermis	2	-45	-17	5.94	0.027	152
	R Posterior Cerebellum	38	-30	-36	5.93	0.028	34
	R Posterior Cerebellum	39	-30	-39	5.67	0.049	4

Note. Changes in regional grey matter volumes in primiparous women between the pre-conception and the early postpartum session corrected for the number of feedings per 24 hours in breastfeeding women. Statistics are extracted from one-sample t-tests performed within the framework of an SPM12 General Linear Model and are one-sided (as is standard in SPM). Results are reported at a statistical threshold of p<0.05 FWE-corrected. P-value at peak voxel (whole-brain FWE corrected) is reported. L = left, R = right.

Supplementary Table 92. Changes in DMN across pregnancy (PRE to POST) corrected for the number of feedings per day.

Contrast	Regions	MNI coordinates			*T*	*P* (FWE-corrected)	Cluster Size (mm³)
		x	*y*	*z*			
Increases	L Cuneus, R Cuneus	6	-84	18	5.96	0.006	189
Decreases	-						

Changes in within-network connectivity in the DMN in primiparous women between the Pre and Post sessions corrected for the number of feedings per day in women breastfeeding their infants. Results are reported at a statistical threshold of $p<0.05$ FWE-corrected. L = left, R = right.

Supplementary Table 93. Correlation between breastfeeding and grey matter volume changes across postpartum period (POST to POST+1y).

Measures	R	p
Total months of breastfeeding	.06	.264

Note. Correlation results between the total number of months of breastfeeding until the Post+1yr session and the observed changes in grey matter volume across the postpartum period (between Post and Post+1yr sessions). It should be noted that for these analyses the R cannot be interpreted as a reflection of the direction of the biological effect, since this statistic is based on patterns of brain changes across the whole brain.

Supplementary Table 94. Correlation between breastfeeding and DMN coherence changes across postpartum period (POST to POST+1y).

Measures	R	p
Total months of breastfeeding	.48	.022

Note. Correlation results between the total number of months of breastfeeding until the Post+1yr session and the observed changes in default mode network coherence across the postpartum period (between Post and Post+1yr sessions).

Supplementary Table 95. Demographics Table

Characteristic	PRG	CTR	Between Group Differences
Sample Size	40	40	
Age at Pre (M ± SD years)	29.35 ± 3.51	29.33 ± 3.57	t = 0.032, p = 0.975
Education (M Verhage Score ± SD)	6.43 ± 0.78	6.65 ± 0.53	t = -1.505, p = 0.137
Secondary school	0	0	
College	19	12	
University	21	28	
Duration Pre-Post (M ± SD days)	509.78 ± 158.91	457.95 ± 81.95	t = 1.833, p = 0.072
Duration without the 3 subjects with delayed session (M ± SD days)	480.32 ± 124.29	457.95 ± 81.95	t = .925, p = 0.359
Natural Conception	37	-	
Assisted Conception	3	-	
Vaginal Birth	31	-	
Caesarean section	9	-	
Breastfeeding	30	-	
Formula Feeding	10	-	
Twins	1	-	
Medical history			
Depression/anxiety	3	5	
Eating Disorder	1		
ADHD	1		
Harlequin Syndrome	1		
Trigeminal neuralgia		1	
Meningitis	1		
Burnout		1	

Note. Demographic information of the sample and between-group differences. PRG = nulliparous women who were pregnant between sessions, CTR = nulliparous women who were not pregnant between sessions.

Supplementary Table 96. Correlation between age, scan interval and educational level with PRE to POST changes in DMN coherence.

Measure	R	p
Age	.06	.715
Time interval between scans	-.063	.699
Educational level (Verhagen)	.023	.888

Note. Correlation results between age (at Pre session), the time interval between the pre-conception and post-pregnancy scans and the educational level according to the Verhagen scale and the observed changes in default mode network coherence.

Supplementary Table 97. Correlation between age, scan interval and educational level with PRE to POST changes in grey matter volume.

Measure	R	p
Age	.16	.144
Time interval between scans	-.08	.516
Educational level (Verhagen)	-.06	.472

Note. Correlation results between age (at Pre session), the time interval between the pre-conception and post-pregnancy scans and the educational level according to the Verhagen scale and the observed changes in grey matter volume based on multivariate regression analyses. It should be noted that for these analyses the R cannot be interpreted as a direct reflection of the direction of the biological effect, since this statistic is based on patterns of brain changes across the whole brain.

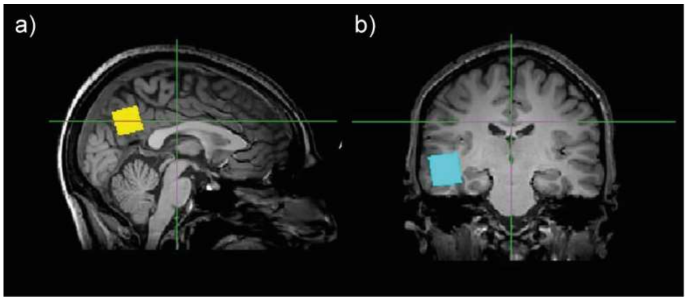

Supplementary Figure 11. Positions of the VOIs used during the Magnetic Resonance Spectroscopy acquisitions. These depict the PCC VOI (a, in yellow) and the STG VOI (b, in blue) of a representable subject at the baseline session, shown in subject T1 space. VOI = Volume of Interest, PCC = Posterior Cingulate Cortex, STG = Superior Temporal Gyrus.

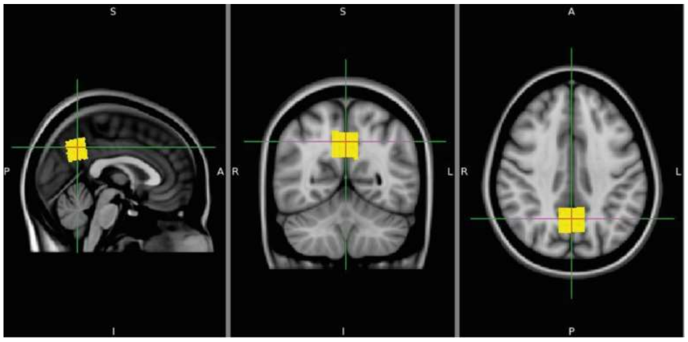

Supplementary Figure 12. Position of the PCC VOI used during the Magnetic Resonance Spectroscopy acquisitions. These images depict the VOI of a representable subject at the baseline session, shown in MNI space. The position of this VOI is at the mean center-of-gravity of all VOIs (x=0 mm, y=-57 mm, z=38 mm). I= Inferior, S=Superior, L=Left, R=Right, A=Anterior, P=Posterior, VOI = Volume of Interest, PCC= Posterior Cingulate Cortex.

Supplementary Table 98. Networks based on spatial sorting.

Neural network	R values
Visual network 1	0.781
Visual network 2	0.524
Visual network 3	0.705
Sensorimotor network	0.665
Default mode network	0.591
Perception/pain network	0.608
Auditory network	0.702
Cognition/language network	0.671
Cerebellum network	0.415
Executive control network	0.464

Note. Overview of the networks derived from spatial sorting by using the components of Smith et al.[3] and their respective R values.

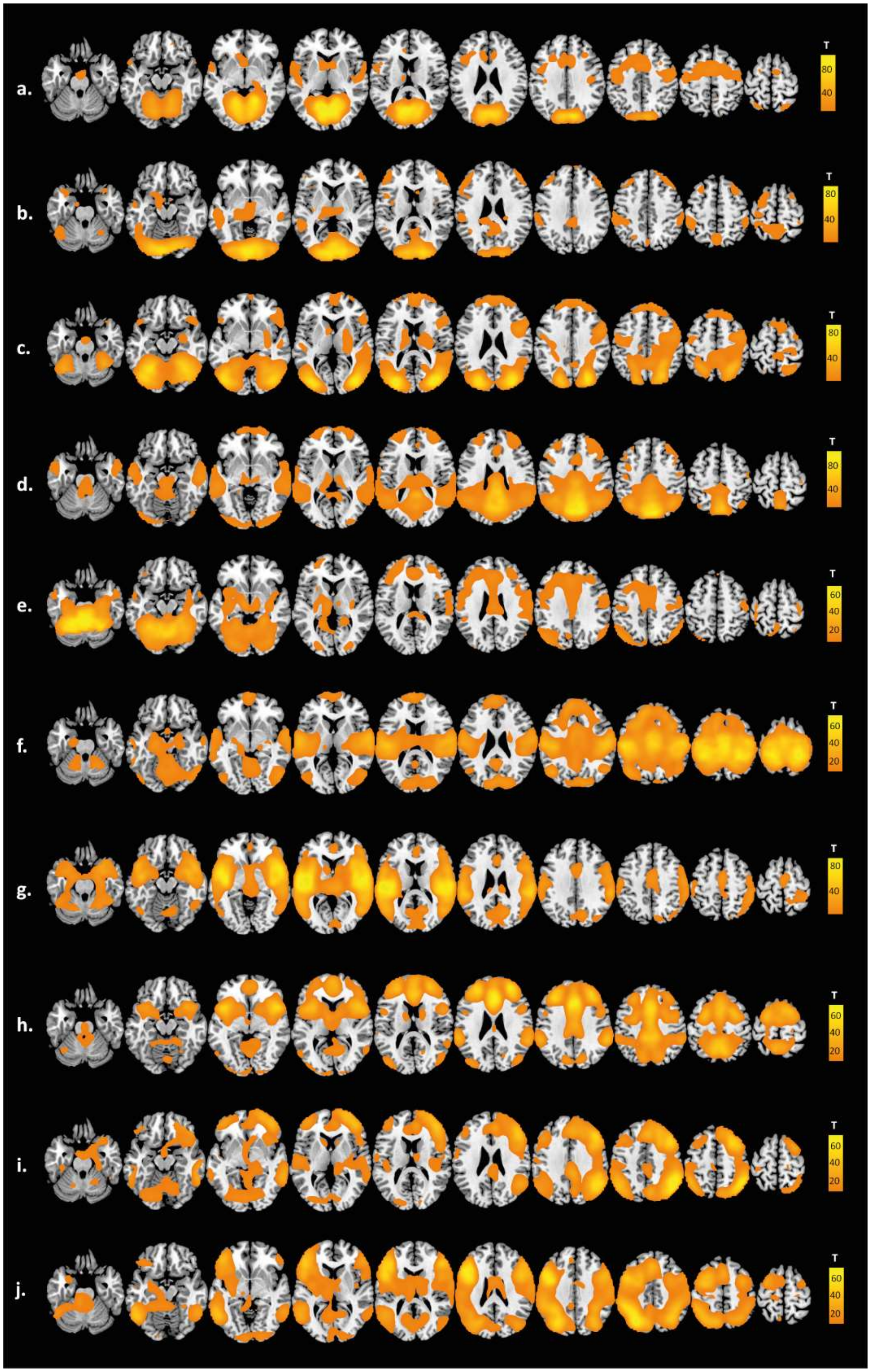

Supplementary Figure 13. Illustration of resting state neural networks. The depicted networks represent the visual network 1 (a), visual network 2 (b), visual network 3 (c), default mode network (d), cerebellar network (e), sensorimotor network (f), auditory network (g), executive function network (h), frontoparietal network 1 (i), frontoparietal network 2 (j) based on the components of Smith et al.[3].

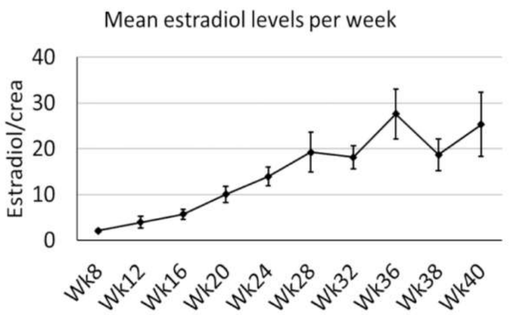

Supplementary Figure 14. Mean levels of estradiol across pregnancy. Estradiol levels (pg/ml) divided by creatinine (mg/dl) to correct for urine concentration are depicted (M±SEM). Levels were extracted from first-morning urine samples available from 30 women in week 8 (N=30), 31 women in week 12 (N=31), 33 women in week 16 (N=33), 34 women in week 20 (N=34), 36 women in week 24 (N=36), 37 women in week 28 (N=37), 33 women in week 32 (N=33), 34 women in week 36 (N=34), 26 women in week 38 (N=26) and 8 women in week 40 of pregnancy (N=8) by means of a high throughput liquid chromatography–tandem mass spectrometry assay. Wk=week

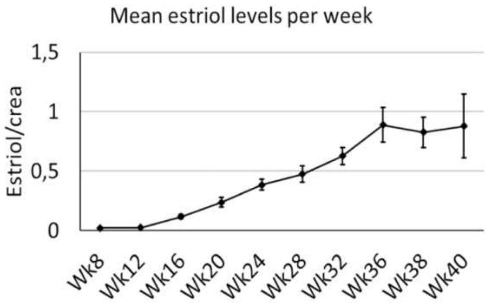

Supplementary Figure 15. Mean levels of estriol across pregnancy. Estriol levels (pg/ml) divided by creatinine (mg/dl) to correct for urine concentration are depicted (M±SEM). Levels were extracted from first-morning urine samples available from 29 women in week 8 (N=29), 32 women in week 12 (N=32), 34 women in week 16 (N=34), 35 women in week 20 (N=35), 36 women in week 24 (N=36), 37 women in week 28 (N=37), 33 women in week 32 (N=33), 34 women in week 36 (N=34), 26 women in week 38 (N=26) and 9 women in week 40 of pregnancy (N=9) by means of a high throughput liquid chromatography–tandem mass spectrometry assay. Wk=week.

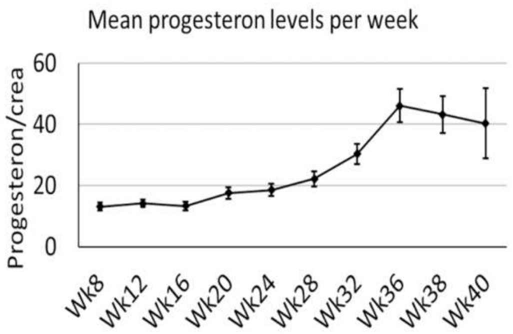

Supplementary Figure 16. Mean levels of progesterone across pregnancy. Progesterone levels (pg/ml) divided by creatinine (mg/dl) to correct for urine concentration are depicted (M±SEM). Levels were extracted from first-morning urine samples available from 30 women in week 8 (N=30), 33 women in week 12 (N=33), 34 women in week 16 (N=34), 35 women in week 20 (N=35), 36 women in week 24 (N=36), 37 women in week 28 (N=37), 32 women in week 32 (N=32), 34 women in week 36 (N=34), 26 women in week 38 (N=26) and 9 women in week 40

of pregnancy (N=9) by means of a high throughput liquid chromatography–tandem mass spectrometry assay. Wk=week.

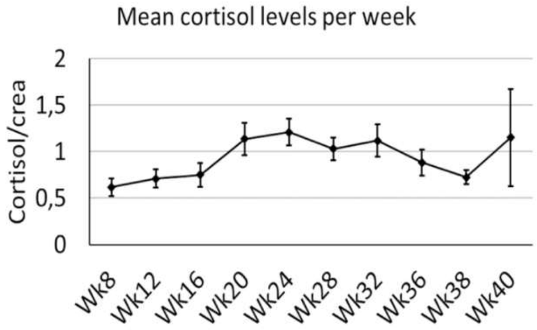

Supplementary Figure 17. Mean levels of cortisol across pregnancy. Cortisol levels (nmol/l) divided by creatinine (mg/dl) to correct for urine concentration are depicted (M±SEM). Levels were extracted from first-morning urine samples available from 30 women in week 8 (N=30), 33 women in week 12 (N=33), 34 women in week 16 (N=34), 35 women in week 20 (N=35), 36 women in week 24 (N=36), 37 women in week 28 (N=37), 33 women in week 32 (N=33), 34 women in week 36 (N=34), 26 women in week 38 (N=26) and 9 women in week 40 of pregnancy (N=9) by means of a high throughput liquid chromatography–tandem mass spectrometry assay. Wk=week.

Supplementary References

1. Yeo, B. T. T. *et al.* Functional Specialization and Flexibility in Human Association Cortex. *Cereb. Cortex* **25**, 3654–3672 (2015).

2. Thomas Yeo, B. T. *et al.* The organization of the human cerebral cortex estimated by intrinsic functional connectivity. *J. Neurophysiol.* **106**, 1125 (2011).

3. Smith, S. M. *et al.* Correspondence of the brain's functional architecture during activation and rest. *Proc. Natl. Acad. Sci. U. S. A.* **106**, 13040–13045 (2009).
