# Supplementary material for: Mapping the effects of pregnancy on resting state brain activity, white matter microstructure, neural metabolite concentrations and grey matter architecture
Source: Nat Commun. 2022 Nov 22;13:6931. doi: 10.1038/s41467-022-33884-8 (PMC9681770; doi:10.1038/s41467-022-33884-8)
Supplement: Supplementary file 2 — Reporting Summary [file 41467_2022_33884_MOESM2_ESM.pdf]

Corresponding author(s): E.A. Hoekzema

Last updated by author(s): Sep 27, 2022

Reporting Summary

Nature Portfolio wishes to improve the reproducibility of the work that we publish. This form provides structure for consistency and transparency in reporting. For further information on Nature Portfolio policies, see our Editorial Policies and the Editorial Policy Checklist.

Statistics

For all statistical analyses, confirm that the following items are present in the figure legend, table legend, main text, or Methods section.

n/a	Confirmed	
☐	☒	The exact sample size (n) for each experimental group/condition, given as a discrete number and unit of measurement
☐	☒	A statement on whether measurements were taken from distinct samples or whether the same sample was measured repeatedly
☐	☒	The statistical test(s) used AND whether they are one- or two-sided<br>*Only common tests should be described solely by name; describe more complex techniques in the Methods section.*
☐	☒	A description of all covariates tested
☐	☒	A description of any assumptions or corrections, such as tests of normality and adjustment for multiple comparisons
☐	☒	A full description of the statistical parameters including central tendency (e.g. means) or other basic estimates (e.g. regression coefficient) AND variation (e.g. standard deviation) or associated estimates of uncertainty (e.g. confidence intervals)
☐	☒	For null hypothesis testing, the test statistic (e.g. F, t, r) with confidence intervals, effect sizes, degrees of freedom and P value noted<br>*Give P values as exact values whenever suitable.*
☒	☐	For Bayesian analysis, information on the choice of priors and Markov chain Monte Carlo settings
☒	☐	For hierarchical and complex designs, identification of the appropriate level for tests and full reporting of outcomes
☐	☒	Estimates of effect sizes (e.g. Cohen's d, Pearson's r), indicating how they were calculated

Our web collection on statistics for biologists contains articles on many of the points above.

Software and code

Policy information about availability of computer code

Data collection	E-prime version 2.0; Acqknowledge software (Acqknowledge 5.0, BIOPAC Systems Inc)
Data analysis	Anatomical MRI images: processing: SPM (version SPM12), implemented in Matlab 7.8, DARTEL tools implemented in SPM (version SPM12), MRIcron (version 7 juli 2012); statistical analyses: SPM (version SPM12), VBM (VBM8 Toolbox);<br>Diffusion-weighted MRI: processing: FMRIB Software Library v6.0 (FSL 6.0), DTI-TK software version 2.3.3; statistical analyses: FSL (6.0);<br>Proton Magnetic Resonance Spectroscopy: processing: LCModel version 6.3-1M; Statistical analyses: SPSS version 25;<br>Resting-state fMRI: processing: DPARSF V4.5; DARTEL tools implemented in SPM (version SPM12), GIFT v4.0b & FNC Toolbox version 2.3 in Matlab; Statistical analyses: SPM (version SPM12), SPSS version 25, VBM (VBM8 Toolbox), Caret software (caret 5), MRIcron (version 7 juli 2012).<br>Physiological data: processing: Brainvision Analyzer (version 2.2); Statistical analyses: Rstudio (1.4.1717).<br>Correlation analyses: Marsbar 0.44, from SPM models in SPSS version 25; PRoNTo version 2.1.3; SPM (version SPM 12).

For manuscripts utilizing custom algorithms or software that are central to the research but not yet described in published literature, software must be made available to editors and reviewers. We strongly encourage code deposition in a community repository (e.g. GitHub). See the Nature Portfolio guidelines for submitting code & software for further information.

Data

Policy information about availability of data

All manuscripts must include a data availability statement. This statement should provide the following information, where applicable:

- Accession codes, unique identifiers, or web links for publicly available datasets
- A description of any restrictions on data availability
- For clinical datasets or third party data, please ensure that the statement adheres to our policy

Source files for the figures are provided in FigShare (http://dx.doi.org/10.6084/m9.figshare.21187240). The raw MRI data along with the correlation variables and group/demographic information for the participants who have provided permission to share their data are provided in the Open Science Framework depository. These data have been deposited in the Open Science Framework depository under the following DOI: http://dx.doi.org/10.17605/OSF.IO/5MT8Z. The deposited data are available open access.

Field-specific reporting

Please select the one below that is the best fit for your research. If you are not sure, read the appropriate sections before making your selection.

☐ Life sciences ☒ Behavioural & social sciences ☐ Ecological, evolutionary & environmental sciences

For a reference copy of the document with all sections, see nature.com/documents/nr-reporting-summary-flat.pdf

Behavioural & social sciences study design

All studies must disclose on these points even when the disclosure is negative.

Study description

Quantitative longitudinal prospective cohort study. Nulliparous women were examined and followed throughout the next few years. Women with and without the intention to become pregnant in the near future took part in the initial session. The final group allocation depended on the transition from nulliparity to primiparity during the course of this study. The final groups are referred to as the pregnant and the control group.

Research sample

Eighty-nine nulliparous women between 20 and 45 years old, with or without the intention to become pregnant in the near future took part in the initial session (PRE session). Of these women, 40 continued in the pregnancy group, and 40 in the control group (POST session). Of these 40 pregnant women, 35 also took part in a late pregnancy session (PRG session). Lastly, 28 of the pregnant women took part in a late postpartum session (POST+1 session).
This sample was chosen to include the age range during which most pregnancies start. The study sample is representative of the Dutch pregnant population, although the included women were relatively highly educated.

Sampling strategy

Sampling of participants was done using different strategies, including snowball sampling, voluntary response sampling and convenience sampling. Final group allocation depended on the transition from nulliparity to primiparity during the course of the study.
This concerns a prospective study that involves following women who wanted to become pregnant from pre-conception, and final sample sizes depended on the number of participants who succeeded in becoming pregnant and delivered their first child during this study. Sample sizes of included participants at baseline were based on our previous experience with such a subject sample in terms of subject attrition and on fertility statistics (Dunson et al. 2004), aiming to reach a minimum of 30 subjects with completed pre-post MRI datasets per group (David et al. 2013; Desmond & Glover. 2002; Murphy & Garavan. 2004; Pajula & Tohka. 2016; Thirion et al. 2007 and Zandbelt et al. 2008).

References

David, S.P., Ware, J.J., Chu, I.M. et al. 2013. Potential reporting bias in fMRI studies of the brain. PLoS One, 8(7), e70104.
Desmond, J.E. & Glover, G.H. 2002. Estimating Sample size in functional MRI (fMRI) neuroimaging studies: Statistical power analyses. Journal of Neuroscience Methods, 118(2), 115-128.
Dunson, D.B., Baird, D.D. & Colombo, B. 2004. Increased infertility with age in men and women. Obstetrics & Gynaecology, 103(1), 51-56.
Murphy, K. & Garavan, H. 2004. An empirical investigation into the number of subjects required for an event-related fMRI study. Neuroimage, 22(2), 879-885.
Pajula, J. & Tohka, J. 2016. How many is enough? Effect of sample size in inter-subject correlation analysis of fMRI. Computational Intelligence and Neuroscience, 2094601.
Thirion, B., Pinel, P., Meriaux, S et al. 2007. Analysis of a large fMRI cohort: Statistical and methodological issues for group analyses. NeuroImage, 35(1), 105-120.
Zandbelt, B.B., Gladwin, T.E., Raemaekers, M. et al. 2008. Within-subject variation in BOLD-fMRI signal changes across repeated measurements: quantification and implications for sample size. NeuroImage, 42(1), 196-206.

Data collection

MRI data: Philips 3T MRI scanner
Physiological data: Biopac MP150 system combined with EMG2-R BioNomadix receiver
Hormone sampling: Liquid chromatography-tandem mass spectrometry; automated cryoscopy
Questionnaires: Qualtrics

	During data collection only the participants and researcher(s) were present. The researcher could not be blinded to the group condition, since pregnancy is clearly visible, and some tasks/samples were only administered to the pregnant group.
Timing	Data collection started 30-06-2016 and ended 14-03-2020.
Data exclusions	All collected anatomical and DTI scans could be used.<br>One subject had to be excluded from the MRS analyses due to a displacement of the VOI at the PRE session. The STS VOI had to be excluded from analysis due to quality issues.<br>For the resting-state analyses, 4 participants from the CTR group had to be removed from the analyses involving the PRE and POST sessions, and 2 PRG participants from the analyses involving the POST + 1 session due to motion (Subjects with frame-wise displacement (FD) exceeding 2mm (for translations) or 2 degrees (rotations) or with a mean FD exceeding 0.2 in any of the sessions were excluded).
Non-participation	A total of 9 women dropped out, for several reasons: illness, loss of interest, no pregnancy or no response. For the late postpartum session, 12 participants dropped out due to COVID-19 restrictions and loss of interest.
Randomization	Randomization is not applicable to this study, since group allocation was decided upon by the participants themselves.

Reporting for specific materials, systems and methods

We require information from authors about some types of materials, experimental systems and methods used in many studies. Here, indicate whether each material, system or method listed is relevant to your study. If you are not sure if a list item applies to your research, read the appropriate section before selecting a response.

Materials & experimental systems

n/a	Involved in the study
☒	☐ Antibodies
☒	☐ Eukaryotic cell lines
☒	☐ Palaeontology and archaeology
☒	☐ Animals and other organisms
☐	☒ Human research participants
☒	☐ Clinical data
☒	☐ Dual use research of concern

Methods

n/a	Involved in the study
☒	☐ ChIP-seq
☒	☐ Flow cytometry
☐	☒ MRI-based neuroimaging

Human research participants

Policy information about studies involving human research participants

Population characteristics	Eighty-nine nulliparous women between 20 and 45 years old, with or without the intention to become pregnant in the near future took part in the initial session (PRE session). Of these women, 40 continued in the pregnancy group, and 40 in the control group (POST session). Of these 40 pregnant women, 35 also took part in a late pregnancy session (PRG session). Lastly, 28 of the pregnant women took part in a late postpartum session (POST+1 session).<br><br>Eight women (3 PRG and 5 CTR) experienced symptoms of depression or anxiety. One woman in the PRG group had previously suffered from an eating disorder, one had contracted meningitis as a baby, one had been diagnosed with ADHD and one suffered from an autonomic disorder (Harlequin syndrome), while one woman in the CTR group had suffered from facial pain and one had experienced a burn-out.<br><br>The participants did not have any contraindications for fMRI, which include metal implants, heart arrhythmia, claustrophia and possible early pregnancy and were all native Dutch speaking.
Recruitment	Participants were recruited using advertisements, flyers (at local GPs and pharmacies) and by word of mouth. This resulted in a relatively highly educated sample. Although level of education did not correlate with the structural or functional outcomes of the study, it remains to be investigated whether the results generalize across educational, cultural and social-economical backgrounds.
Ethics oversight	Ethics Review Board of the Leiden University Medical Center.

Note that full information on the approval of the study protocol must also be provided in the manuscript.

Magnetic resonance imaging

Experimental design

Design type	resting-state
Design specifications	n/a (resting-state, fixation cross)

Behavioral performance measures | None

Acquisition

Imaging type(s): structural, diffusion, spectroscopy, functional

Field strength: 3T

Sequence & imaging parameters:
Structural: 3D T1-weighted images were acquired (Repetition Time (TR) = 9.8 ms; Echo Time (TE) = 4.6 ms; Flip Angle = 8°; voxel size = 0.875 × 0.875 × 1.20 mm3; Field of View= 224 x 178 x 168 mm);
Resting-state functional: T2*-weighted whole-brain echo-planar images (EPIs) with the following acquisition parameters: Repetition Time (TR) = 2.2 s, Echo Time (TE) = 30 ms, Flip Angle = 80°; Field of View = 220 x 220 x 111.65, 37 slices.
Diffusion: fast MRI (SENSE) factor = 3; Flip Angle 90°; 75 slices of 2 mm; no slice gap; reconstruction matrix 128 × 128; Field of View= 240 × 240 mm; Echo Time (TR) = 69 ms; Repetiton Time (TR) = 7315 ms;
Spectroscopy: single-voxel point-resolved spectroscopy (PRESS) localization (Repetition Time (TR) = 2000 ms; Echo Time (TE) = 37 ms; 128 averages, 2 dummy scans, and 16 reference scans without water suppression).

Area of acquisition: Whole-brain, except for spectroscopy which included 2 volumes of interest (Precuneus/posterior cingulate cortex (PCC) & Right superior temporal gyrus (STG)).

Diffusion MRI: [x] Used [ ] Not used

Parameters: 30 Diffusion weighted volumes (b=1000s/mm2) and 5 Diffusion-unweighted volumes. Single shell data without cardiac gating.

Preprocessing

Preprocessing software:
Structural: SPM12 in Matlab 7.8: longitudinal symmetric diffeomorphic modeling pipeline: longitudinal registration tool (rigid-body registration, intensity inhomogeneity correction and nonlinear diffeomorphic registration), tissue segmentation, 10 mm FWHM smoothing kernel.
Diffusion: FSL 6.0: Topop (susceptibility correction) and eddy (current-induced distortions & movement correction); DTIfit.
Resting-state functional: DPARSF V4.5: slice time correction, realignment, co-registration with anatomical, frame-wise displacement (motion)
Spectroscopy: LCModel version 6.3-1M.

Normalization:
Structural: DARTEL (non-linear), smoothed with 10-mm FWHM smoothing kernel;
Diffusion: DTI-TK version 2.3.3 (non-linear)
Functional: DARTEL (non-linear), smoothed with 10 mm3 FWHM Gaussian kernel
Spectroscopy: no normalization, quantification within individual volume of interest.

Normalization template:
Structural: MNI space
Diffusion: Group specific template
Functional: MNI space
Spectroscopy: no normalization.

Noise and artifact removal:
Diffusion: susceptibility distortion correction (FSL topup) and eddy current-induced distortion and movement correction (FSL eddy)
Functional: slice-timing correction and realignment. Subjects with frame-wise displacement (FD) exceeding 2mm (for translations) or 2 degrees (rotations) or with a mean FD exceeding 0.2 in any of the sessions were excluded.

Volume censoring: We did not use volume censoring in the functional analyses since subjects with frame-wise displacement (FD) exceeding 2mm (for translations) or 2 degrees (rotations) or with a mean FD exceeding 0.2 in any of the sessions were excluded.

Statistical modeling & inference

Model type and settings: Group spatial independent component analysis (ICA).

Effect(s) tested: We did not use any task during fMRI.

Specify type of analysis: [ ] Whole brain [ ] ROI-based [x] Both

Anatomical location(s):
Structural: Spatial correspondance with cognitive components and resting-state neural networks extracted from Yeo et al., 2011 & Yeo et al., 2015
Diffusion: voxels in white matter skeleton (TBSS)
Resting-state: Comparison to resting-state networks from Smith et al., 2009.
Spectroscopy: Volumes of interest are acquisition-dependent.

Statistic type for inference (See Eklund et al. 2016):
Structural: voxel-wise
Diffusion: voxel-wise (TBSS)
Resting-state functional: correlations between resting-state networks (clusters) based on ICA.

	Spectroscopy: -.
Correction	Structural: FWE-corrected across the whole-brain<br>Diffusion: FWE-correction across the whole-brain<br>Resting-state functional: FWE-corrected<br>Spectroscopy: Bonferroni correction, adjusted according to the mean correlation between the examined variables.

Models & analysis

n/a	Involved in the study
☐	☒ Functional and/or effective connectivity
☒	☐ Graph analysis
☐	☒ Multivariate modeling or predictive analysis

Functional and/or effective connectivity	Between-network connectivity (Pearson's correlation)
Multivariate modeling and predictive analysis	Multivariate kernel regression analyses, kernel ridge regression, leave-one-out cross-validation.
